# The 1,5-Benzodiazepin-2-Ones: A Review on Synthetic Strategies, Reactivity and Functional Applications

**DOI:** 10.3390/molecules31122004

**Published:** 2026-06-08

**Authors:** Hasan Mtiraou, Ameni Ghabi, Hanan Al-Ghulikah, Mezna Saleh Altowyan

**Affiliations:** 1 Laboratory of Heterocyclic Chemistry Natural Products and Reactivity/CHPNR, Department of Chemistry, Faculty of Science of Monastir, University of Monastir, Monastir 5000, Tunisia; mtiraoui1hasan@gmail.com (H.M.); ame.ghab1992@gmail.com (A.G.); 2 Department of Chemistry, College of Science, Princess Nourah bint Abdulrahman University, Riyadh 11671, Saudi Arabia; msaltowyan@pnu.edu.sa

**Keywords:** 1,5-benzodiazepin-2-one, benzodiazepines, *N*-heterocycles, synthesis, medicinal chemistry, materials chemistry

## Abstract

1,5-Benzodiazepin-2-one is a significant class of heterocyclic compounds that have attracted considerable attention due to these versatile scaffolds and their multifunctional properties. Owing to the presence of a fused benzene–diazepine core, these compounds display rich chemical reactivity and various physicochemical characteristics, making them valuable structures in both fundamental and applied research. This review provides a comprehensive and critical overview of the advances reported to date on 1,5-benzodiazepin-2-ones, with particular emphasis on their diverse synthetic methodologies, reaction mechanisms, and structure–property relationships. Classical and modern synthetic approaches are discussed, including condensation reactions, multicomponent processes, catalytic and green chemistry approaches, as well as recent developments toward more sustainable and efficient protocols. The intrinsic reactivity of the 1,5-benzodiazepin-2-one framework enables access to structurally diverse derivatives. Furthermore, the review summarizes the broad range of applications of these heterocycles, especially in medicinal chemistry, where they have demonstrated significant biological activities, as well as in corrosion inhibition and photoluminescent materials. Finally, current challenges, limitations, and future perspectives are outlined to provide guidance for further research and development of 1,5-benzodiazepin-2-one-based systems.

## 1. Introduction

Heterocyclic compounds represent one of the most versatile and widely studied classes of organic molecules, a status owed to their ubiquitous presence in natural products and their crucial roles in biological processes [1]. Among them, nitrogen-containing heterocycles hold a particularly prominent place in medicinal chemistry, prized for their remarkable structural diversity, tuneable reactivity, and broad spectrum of biological activities [2]. The past few decades have witnessed significant advancements in the synthesis and functionalization of these compounds, driven by their proven therapeutic value and potential in rational drug design.

Benzodiazepines constitute a fundamental class of fused bicyclic *N*-heterocycles within this landscape. The field was inaugurated with the discovery of chlordiazepoxide (Librium) by Leo Sternbach at Hoffmann-La Roche in 1955, which was subsequently marketed in 1960. This breakthrough was followed by the introduction of diazepam (Valium) in 1963, which became one of the most widely prescribed psychotropic drugs globally [3]. Despite their discovery over half a century ago, research into benzodiazepines remains highly active, with continuous efforts focused on developing analogues with enhanced pharmacological profiles [4].

**Figure 1:**
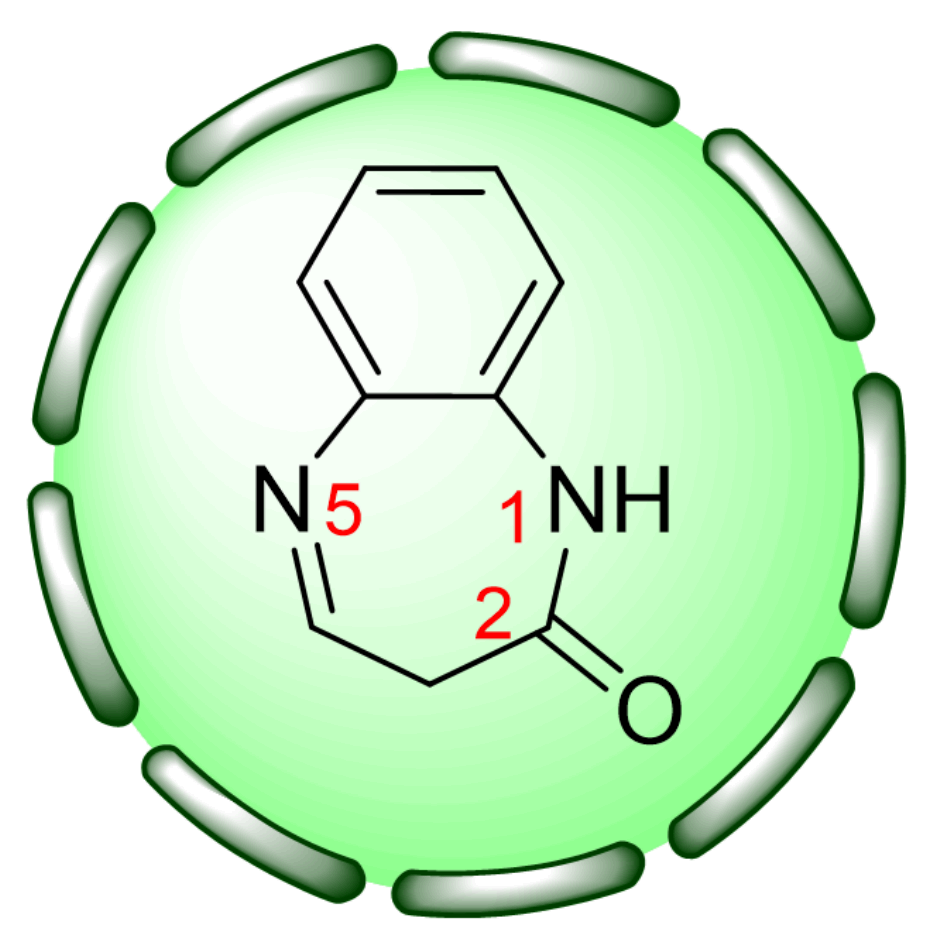
Core structure of the 1,5-benzodiazepin-2-one scaffold.

While 1,4-benzodiazepines are extensively documented, their 1,5-isomers have garnered increasing interest due to a distinct and expanding biological relevance. Historically associated with neuropsychiatric disorders, the scope of 1,5-benzodiazepines has broadened considerably over the past decade, now encompassing potential applications in anticancer, antiviral, and cardiovascular therapies [5,6,7]. This contribution focuses specifically on the 1,5-benzodiazepin-2-one subclass, a scaffold characterized by a seven-membered diazepine ring fused to a benzene ring, with nitrogen atoms at the 1 and 5 positions and a carbonyl group at position 2 (Figure 1).

**Figure 2:**
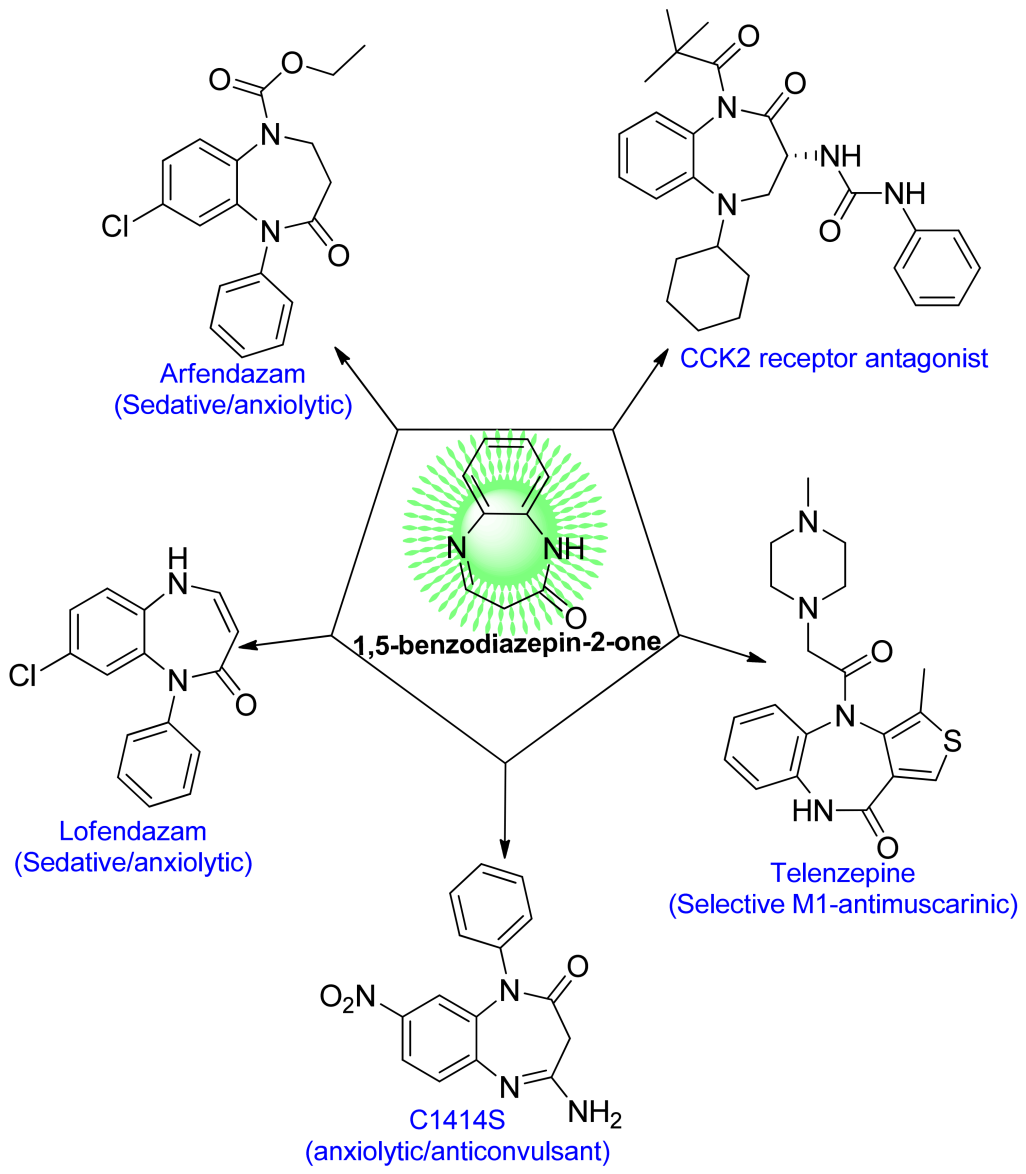
Clinically relevant and bioactive 1,5-benzodiazepin-2-one derivatives.

The 1,5-benzodiazepin-2-one framework is recognized as a privileged structure in drug discovery. Several of its derivatives have advanced clinical use, including the anxiolytic agents arfendazam and lofendazam, and the antisecretory drug telenzepine. Furthermore, compounds like C1414S have demonstrated promising anxiolytic and anticonvulsant properties in preclinical studies (Figure 2) [8,9,10]. This significant biological potential is complemented by a rich and versatile chemical reactivity.

**Figure 3:**
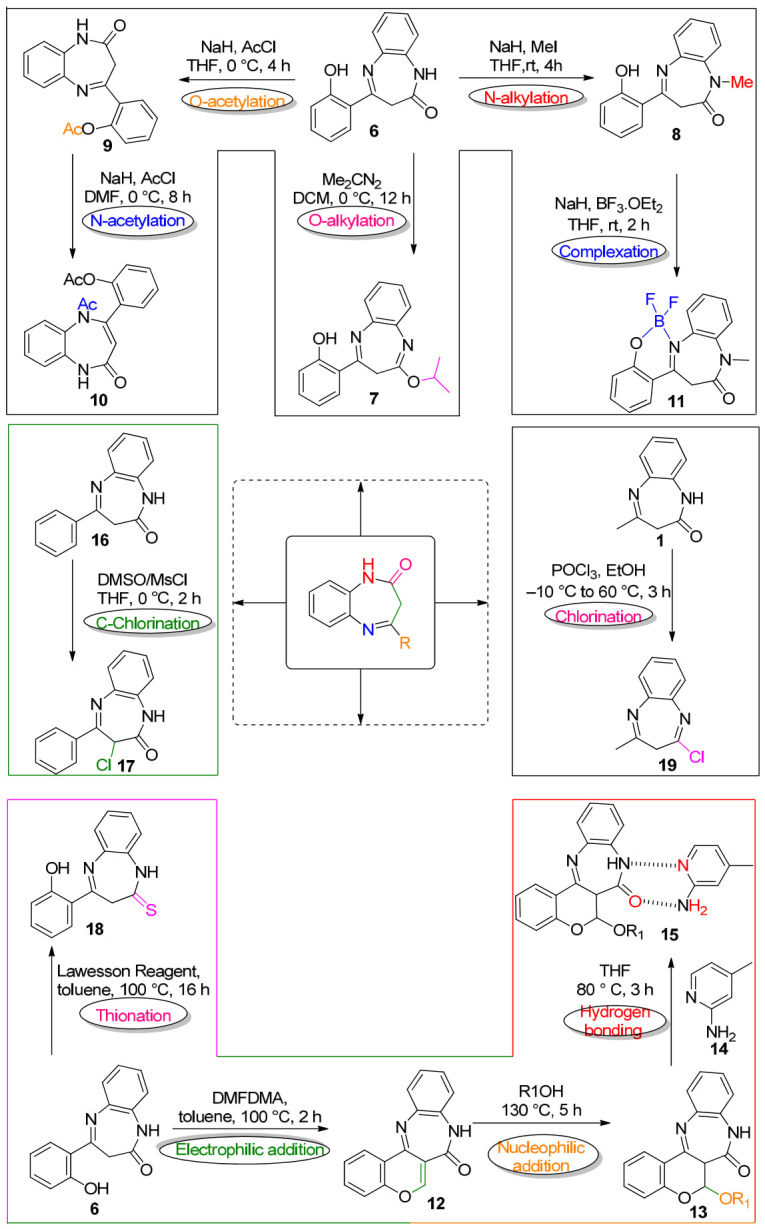
Variety of organic transformations.

The 1,5-benzodiazepin-2-one scaffold exhibits a remarkable propensity to undergo a wide range of organic transformations—such as *N*-alkylation, chlorination, *N*-acetylation, and thionation—at its various reactive sites (Figure 3) [11,12,13,14,15,16,17,18,19]. For instance, the adjacent carbonyl groups activate the methylene protons at position 3, facilitating the abstraction of the acidic hydrogen by a base. The resulting carbanion is a key intermediate that can participate in diverse nucleophilic addition reactions.

In view of its synthetic accessibility, functional versatility, and broad applicability, a consolidated review of the 1,5-benzodiazepin-2-one scaffold is highly pertinent. This article provides a comprehensive and systematic overview of the recent work accomplished over the past decade. It details the diverse synthetic routes, catalytic systems, and solvents employed, as well as the wide range of pharmacological activities and material applications exhibited by these derivatives. This work aims to serve as a valuable resource for researchers navigating the development of this promising class of molecules.

## 2. Chemical Properties of 1,5-Benzodiazepin-2-Ones

### 2.1. Tautomerism

**Figure 4:**
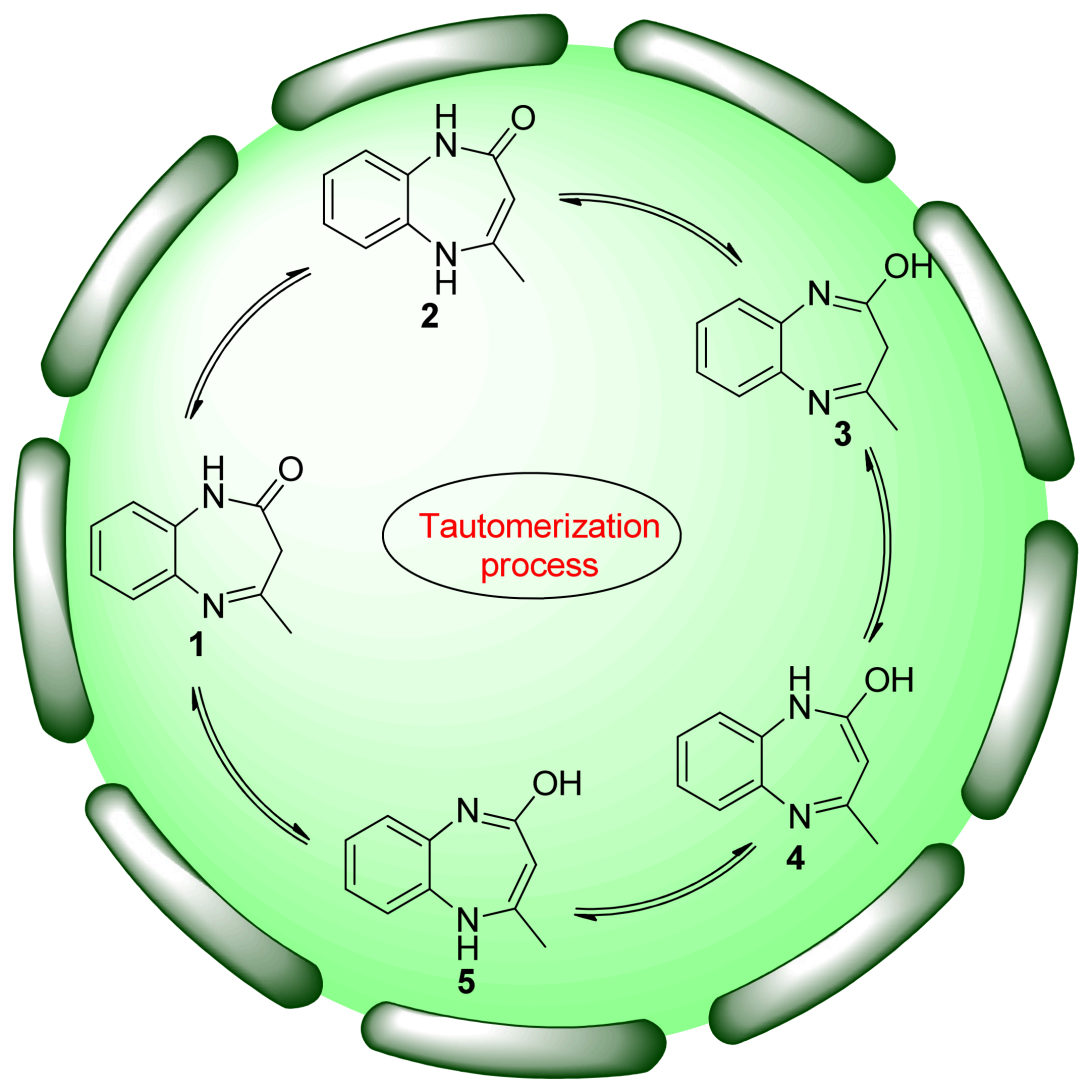
General tautomerization process of 4-methyl-1,5-benzodiazepin-2-one.

1,5-Benzodiazepin-2-ones generally exist as two stable keto tautomers, designated as 3*H*-2-oxo (**1**) and 1,5-di*H*-2-oxo (**2**) [20]. The tautomerization process involves proton migration and is highly solvent-dependent. Density functional theory (DFT) calculations on 4-methyl-1,3-dihydro-2*H*-1,5-benzodiazepin-2-one have confirmed that the keto forms (**1** and **2**) are more stable than the enol tautomers in the gas phase, which is attributed to stronger intramolecular hydrogen bonding [21]. However, the tautomeric equilibrium is significantly influenced by protic solvents such as ethanol, where intermolecular hydrogen bonding facilitates proton transfer and lowers the free energy barriers. Notably, the enol tautomer **4** (μ = 6.77 D) becomes more stable in ethanol due to its larger dipole moment and stronger solvent interactions (Figure 4).

### 2.2. Conformational and Stereochemical Aspects

**Figure 5:**
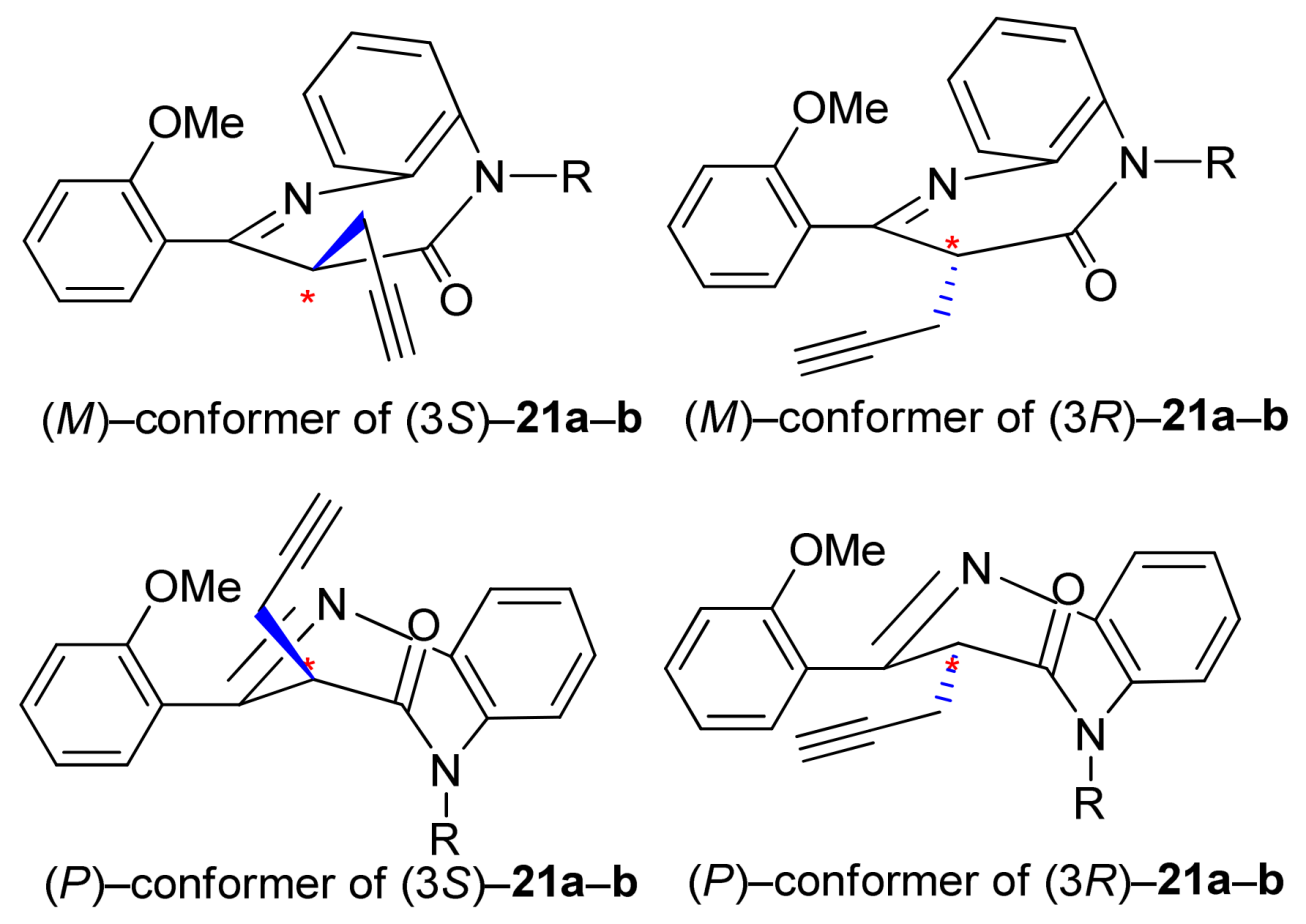
Representation of stereoisomeric forms of **21a** and **21b**.

**Scheme 1:**
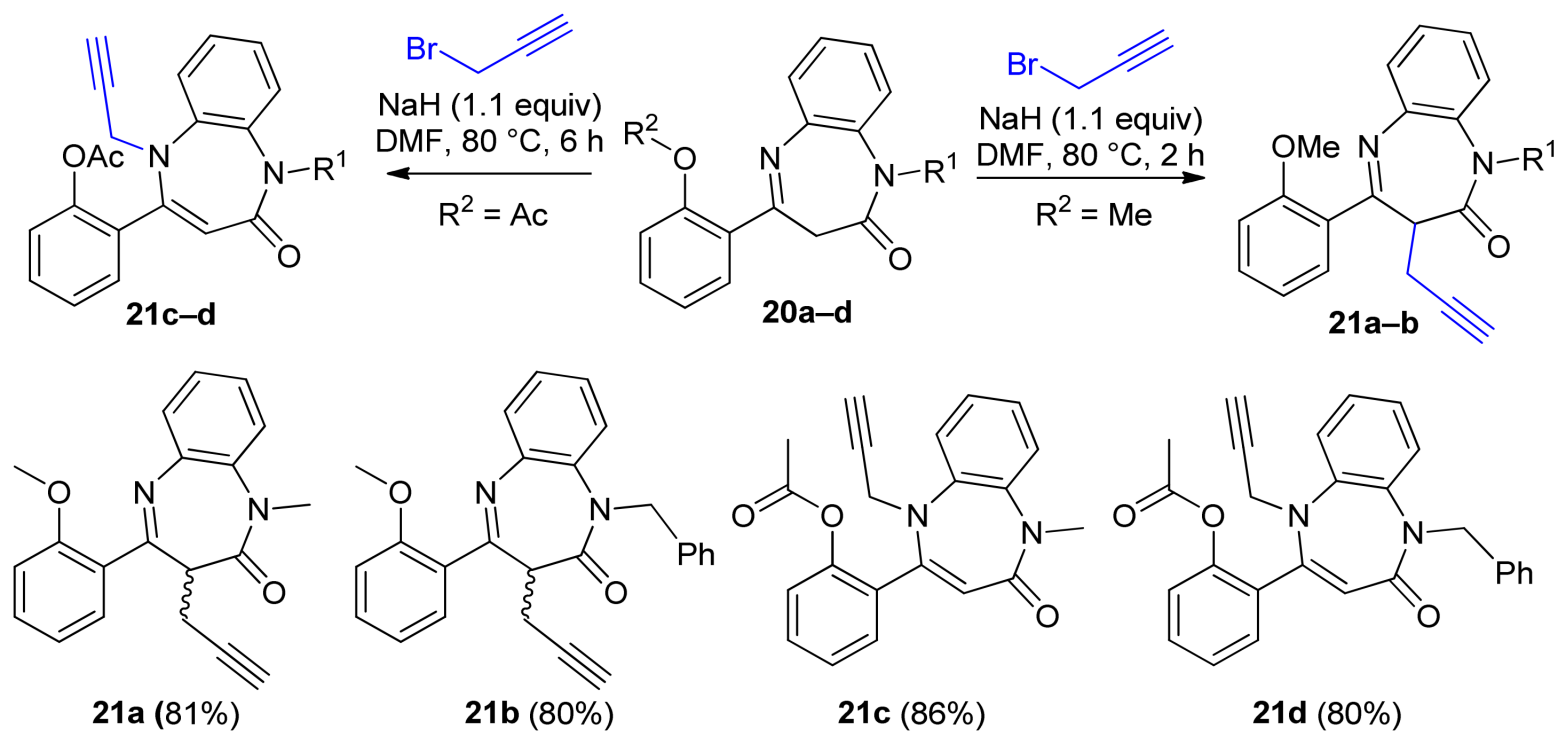
Synthesis of propargylated 1,5-benzodiazepin-2-ones.

To establish a convenient divergent synthesis of functionalized 1,5-benzodiazepin-2-ones, Amel and co-workers [22] investigated the regioselective introduction of a propargyl group at either the 3- or 5-position of the central ring (Scheme 1). Separately, Danny et al. demonstrated that 1,5-benzodiazepin-2-ones can adopt either (*M*)- or (*P*)-conformations [15,23], a property arising from the well-documented nonplanarity of the diazepinone ring, which is traditionally described using these helical descriptors [24]. For compounds **21a** and **21b**, the presence of an asymmetric centre at C3 creates the possibility of both R and S configurations. In principle, this could lead to four stereoisomers: (*P*, *R*), (*P*, *S*), (*M*, *R*), and (*M*, *S*) (Figure 5). However, NMR analysis revealed the presence of only two stereoisomers.

## 3. Synthetic Strategies

Various synthetic strategies have been developed for the preparation of 1,5-benzodiazepin-2-ones. These methodologies differ in their choice of starting materials, reaction conditions, and the obtained yields.

### 3.1. From o-Phenylenediamine

#### 3.1.1. From Diamines and β-Ketoesters

**Scheme 2:**
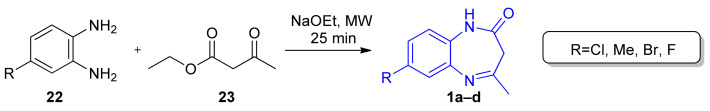
Condensation of diamines and *β*–ketoester under MW.

**Scheme 3:**
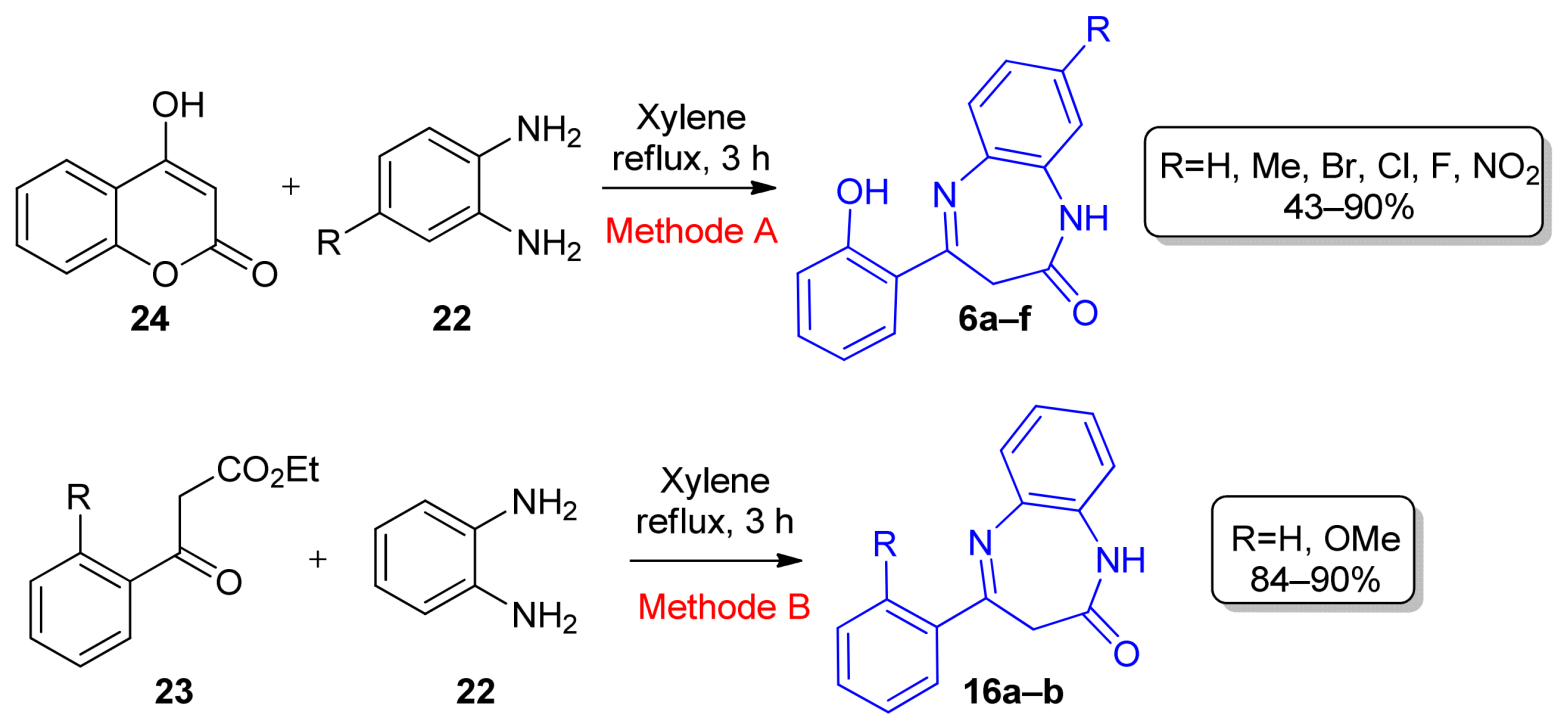
Classical methods for the synthesis of 1,5-benzodiazepin-2-one moieties.

**Scheme 4:**
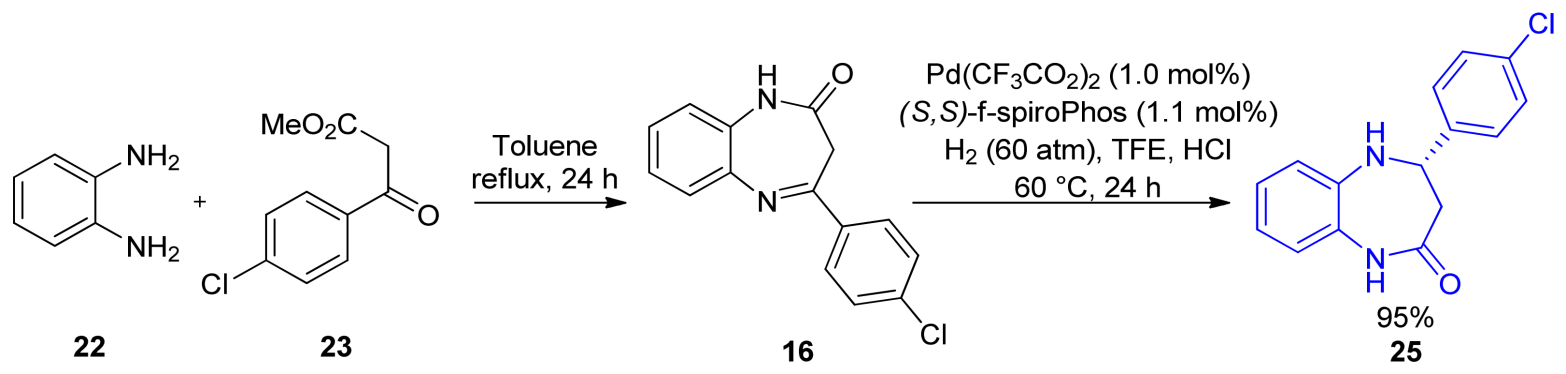
Synthesis of chiral 1,5-benzodiazepin-2-one via **25**.

**Scheme 5:**
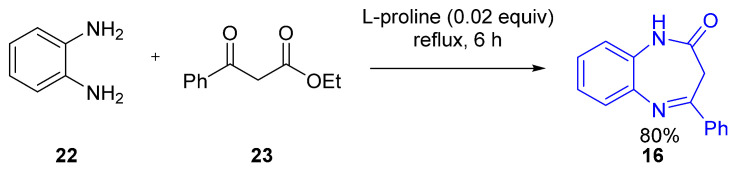
Synthesis of 1,5-benzodiazepin-2-one catalyzed by *L*-proline.

Rishipathak and co-workers [7] synthesized 1,5-benzodiazepine derivatives **1a–d** by reacting an equimolar mixture of ethyl acetoacetate and a 4-substituted *o*-phenylenediamine in a sodium ethoxide solution, followed by microwave irradiation at 420 W for approximately 25 min (Scheme 2). Hasan et al. [11] reported the synthesis of a series of 4-phenyl-1,3-di*H*-1,5-benzodiazepin-2-ones **6a–f** with various aromatic substitutions, following known procedures. These compounds were obtained by reacting 4-hydroxycoumarin with unsubstituted or 4-substituted *o*-phenylenediamines in refluxing *p*-xylene for 3 h. Additionally, 1,5-benzodiazepin-2-ones **16a–b** were synthesized via the reaction of *o*-phenylenediamine with benzoylacetates (Scheme 3). Xie’s group [25] reported the first Pd-catalyzed asymmetric hydrogenation of seven-membered cyclic imines using a novel Pd–f-spiroPhos complex, affording a series of chiral 1,5-benzodiazepin-2-ones in high yields with excellent enantioselectivities (up to >99% ee) (Scheme 4). Furthermore, Papori and co-workers [26] developed a novel method using *L*-proline (20 mol%) as an efficient catalyst for the synthesis of 1,5-benzodiazepin-2-one **16**. They proposed a mechanism involving initial *L*-proline–mediated enamine formation, followed by intramolecular condensation with the second amino group, ultimately affording the target compound **16** (Scheme 5).

#### 3.1.2. From Diamines and 1,3-Acetonedicarboxylate

**Scheme 6:**
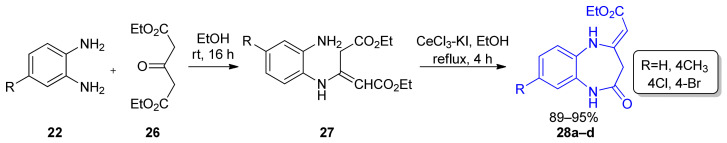
Coupling of diamines and 1,3-acetonedicarboxylate.

Wu and Wang [27] developed an eco-friendly method for the synthesis of 1,5-benzodiazepine-2-one derivatives **28a–d** from *o*-phenylenediamine derivatives (OPDs) and 1,3-acetonedicarboxylate using CeCl_3_–KI or γ-Fe_2_O_3_@SiO_2_/CeCl_3_ as catalysts in ethanol. Using 4-substituted OPDs, four derivatives were obtained in 35–45 min with yields of 89–95%. The protocol proceeds via an enamine ester intermediate at room temperature, followed by cyclization under reflux, offering mild conditions, non-toxic solvents, simple operation, and excellent efficiency (Scheme 6).

#### 3.1.3. From Diamines and Ethyl Cyanoacetate

**Scheme 7:**
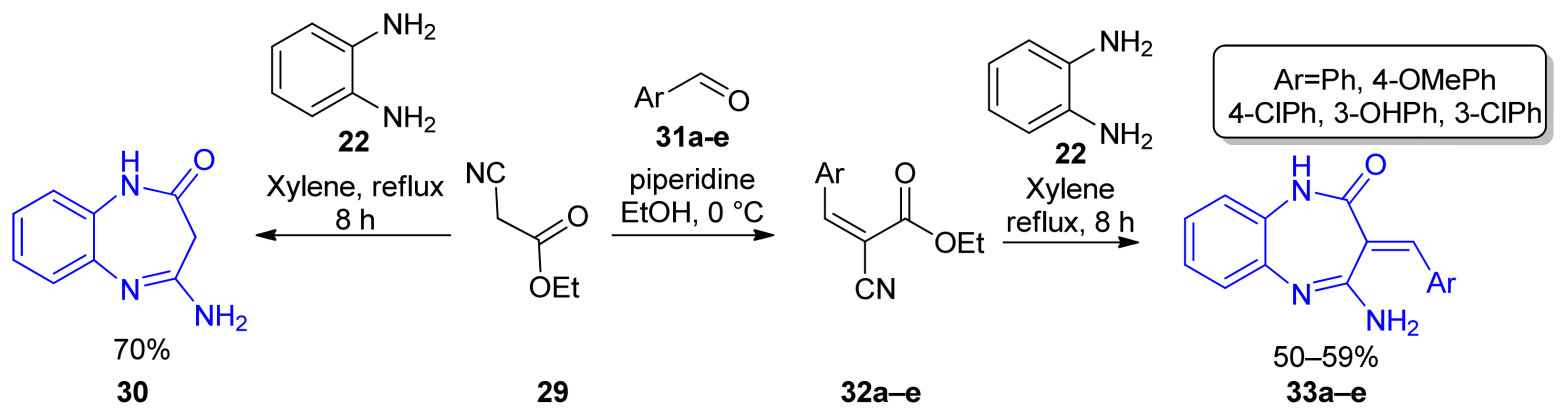
Coupling of *o*-phenylenediamine and ethyl cyanoacetate.

As outlined in Scheme 7, Hanaa and co-workers [28] developed a stepwise synthesis of 1,5-benzodiazepin-2-ones **33a–e** under simple conditions. *α*-Ethylcyanocinnamate derivatives **32a–e** were first synthesized via a Knoevenagel condensation of ethyl cyanoacetate with various aldehydes in ethanol, using piperidine as a catalyst at 0 °C. The subsequent reaction of **32a–e** with *o*-phenylenediamine in xylene afforded the target 1,5-benzodiazepin-2-ones **33a–e**. An alternative one-pot route, reacting ethyl cyanoacetate directly with OPD in xylene, yielded compound **30**.

#### 3.1.4. From Diamines and Acrylamide

**Scheme 8:**
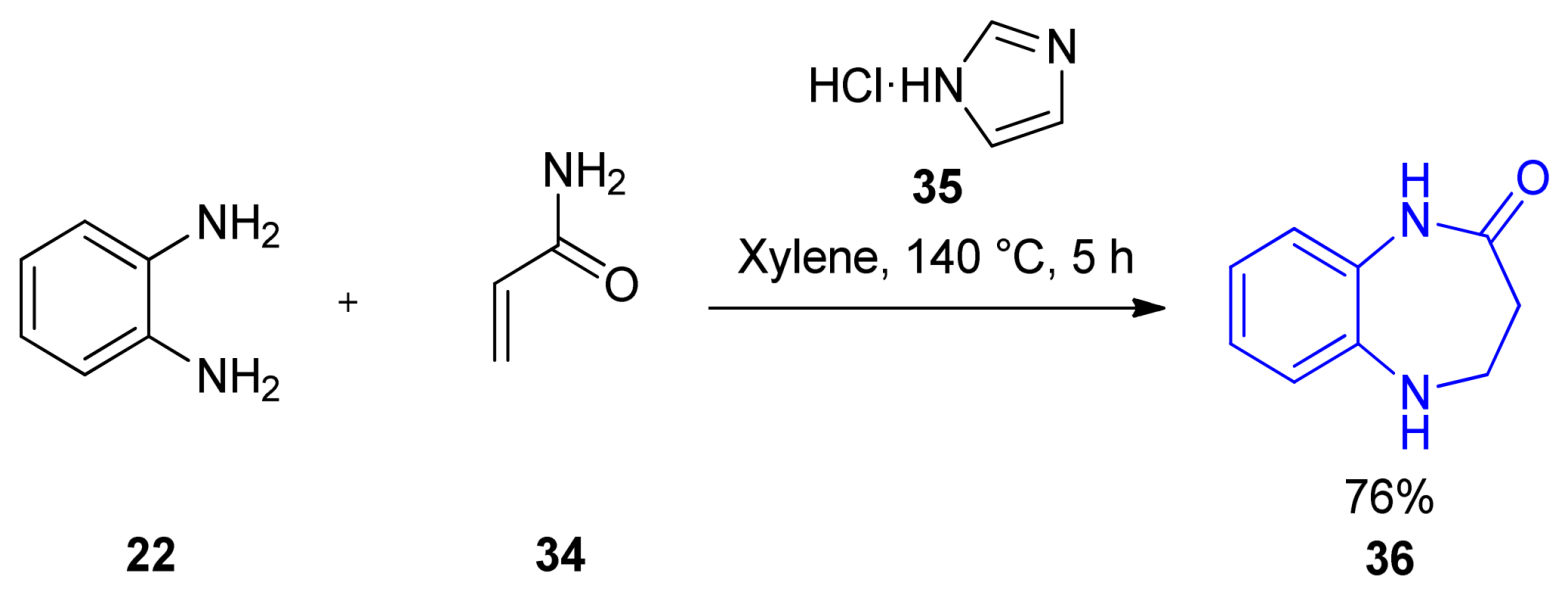
Coupling of *o*-phenylenediamine with acrylamide.

Dai’s group [29] reported that the reaction of *o*-phenylenediamine with acrylamide at 140 °C for 5 h afforded 1,3,4,5-tetrahydro-1,5-benzodiazepin-2-one **36** in very good yield. Building on this finding, they developed an environmentally friendly procedure for the efficient synthesis of Aza-Michael adducts, employing imidazolium chloride as a green catalyst for amino group protection en route to benzodiazepine derivatives (Scheme 8).

#### 3.1.5. From Diamines and Diketene

**Scheme 9:**
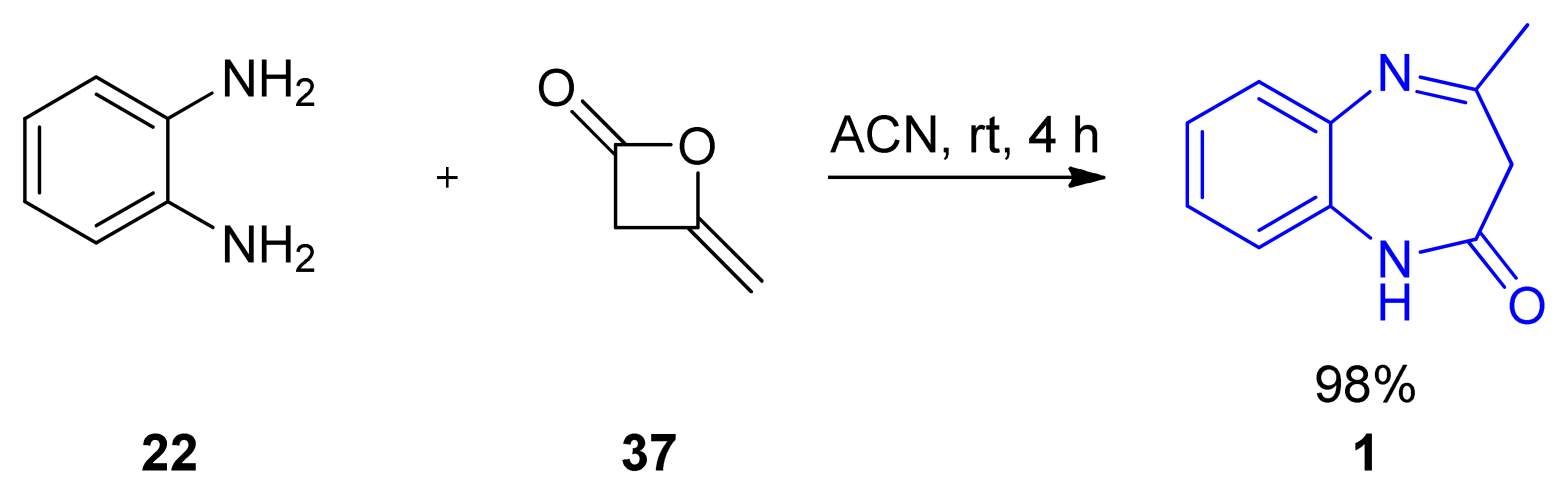
Synthesis of 1,5-benzodiazepin-2-one via condensation of o-phenylene diamine and diketene.

Ahmad and co-workers [30] developed a novel and efficient method for the preparation of 1,5-benzodiazepin-2-one **1** in good yield, via the condensation of *o*-phenylenediamine with diketene at ambient temperature (Scheme 9).

#### 3.1.6. From Diamines and Acetylated Baylis–Hillman Adduct

**Scheme 10:**
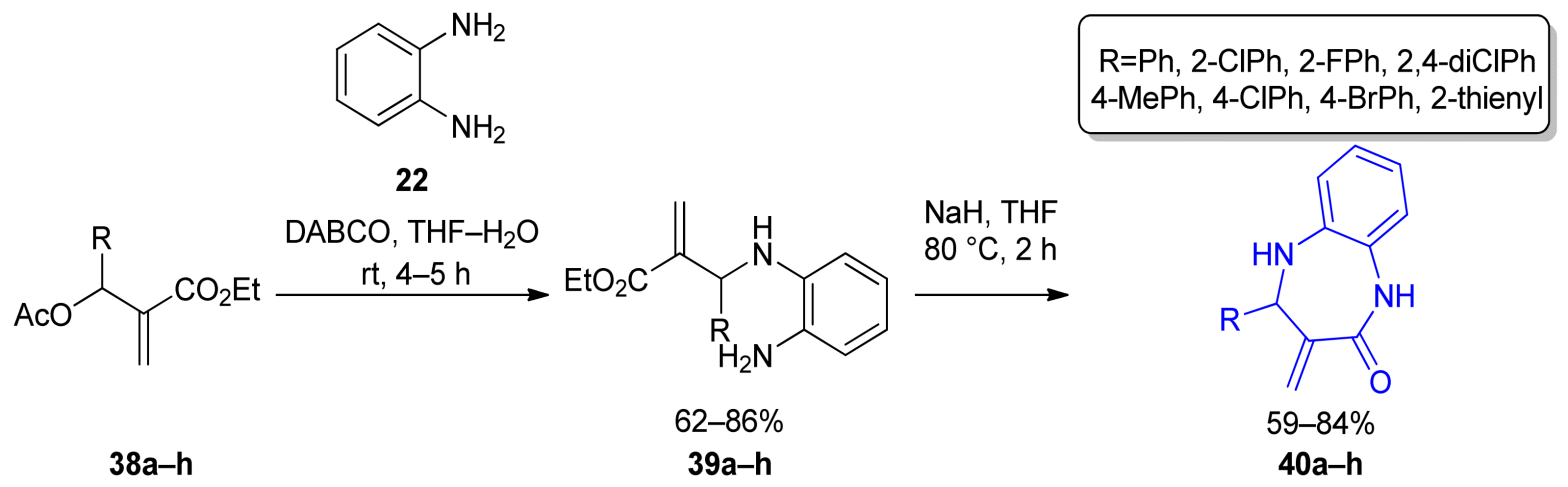
Synthesis of 1,5-benzodiazepin-2-ones from an acetylated Baylis–Hillman adduct and *o*-phenylenediamine.

Pathack et al. reported the synthesis of acetyl derivatives **38a–h** of the Baylis–Hillman adduct following literature procedures [31]. An SN2 nucleophilic substitution of *o*-phenylenediamine with **38a–h** in the presence of 1,4-Diazabicyclo[2.2.2]octane (DABCO) in a THF–water system afforded diamino esters **39a–h** in excellent yields. Subsequent treatment with sodium hydride in toluene at 80 °C provided the corresponding 1,5-benzodiazepin-2-ones **40a–h** (Scheme 10).

#### 3.1.7. From Diamines and Spiroepoxy Lactone

**Scheme 11:**
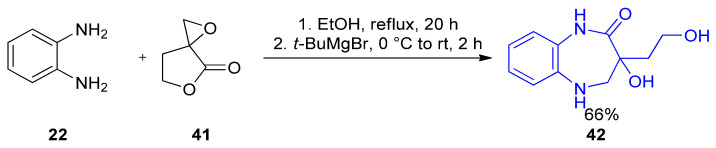
Synthesis of 1,5-benzodiazepin-2-one from a spiroepoxy lactone and phenylenediamine.

Otto and co-workers [32] demonstrated that the reaction of a spiroepoxy lactone with *o*-phenylenediamine in refluxing ethanol, mediated by *t*-BuMgBr, affords 1,5-benzodiazepin-2-one **42** via amide formation. However, the scope of this approach appears limited, as the only studied spiroepoxy lactone bearing a substituent on the epoxide ring proved to be an ineffective substrate (Scheme 11).

#### 3.1.8. From Diamines and Ethyl 3-Phenylglycidate

**Scheme 12:**
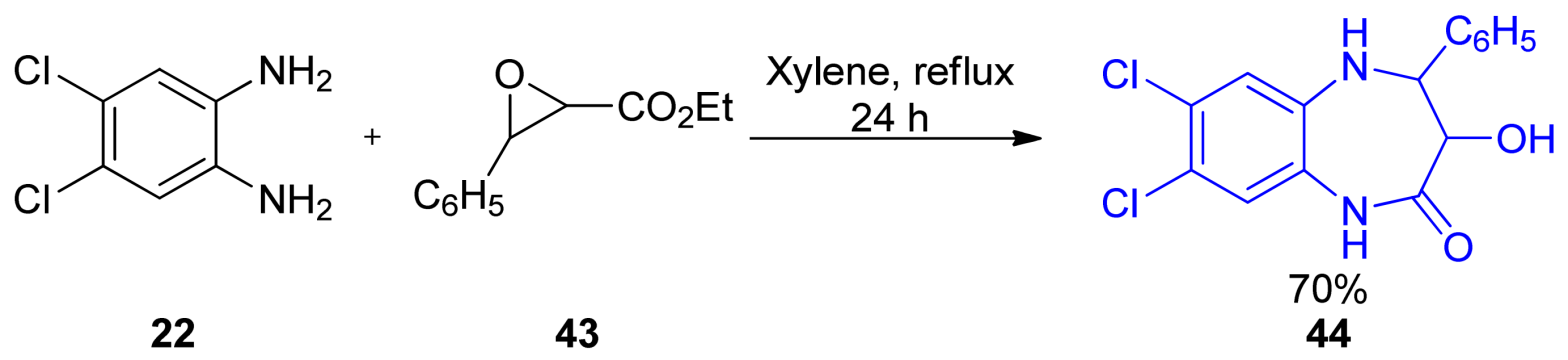
Synthesis of a 3-hydroxy-1,5-benzodiazepin-2-one from a substituted *o*-phenylenediamine and ethyl 3-phenylglycidate.

Rida et al. [33] developed a new method for preparing 3-hydroxy-1,5-benzodiazepin-2-one **44**. Refluxing 4,5-dichloro-*o*-phenylenediamine with a cis/trans mixture of ethyl 3-phenylglycidate in xylene for 24 h afforded the corresponding amino ester. Subsequent reflux in xylene for 48 h yielded 7,8-dichloro-3-hydroxy-4-phenyl-1,3,4,5-tetrahydro-1,5-benzodiazepin-2-one **44** (Scheme 12).

#### 3.1.9. From Diamines and α,β-Unsaturated Acylazoliums

**Scheme 13:**
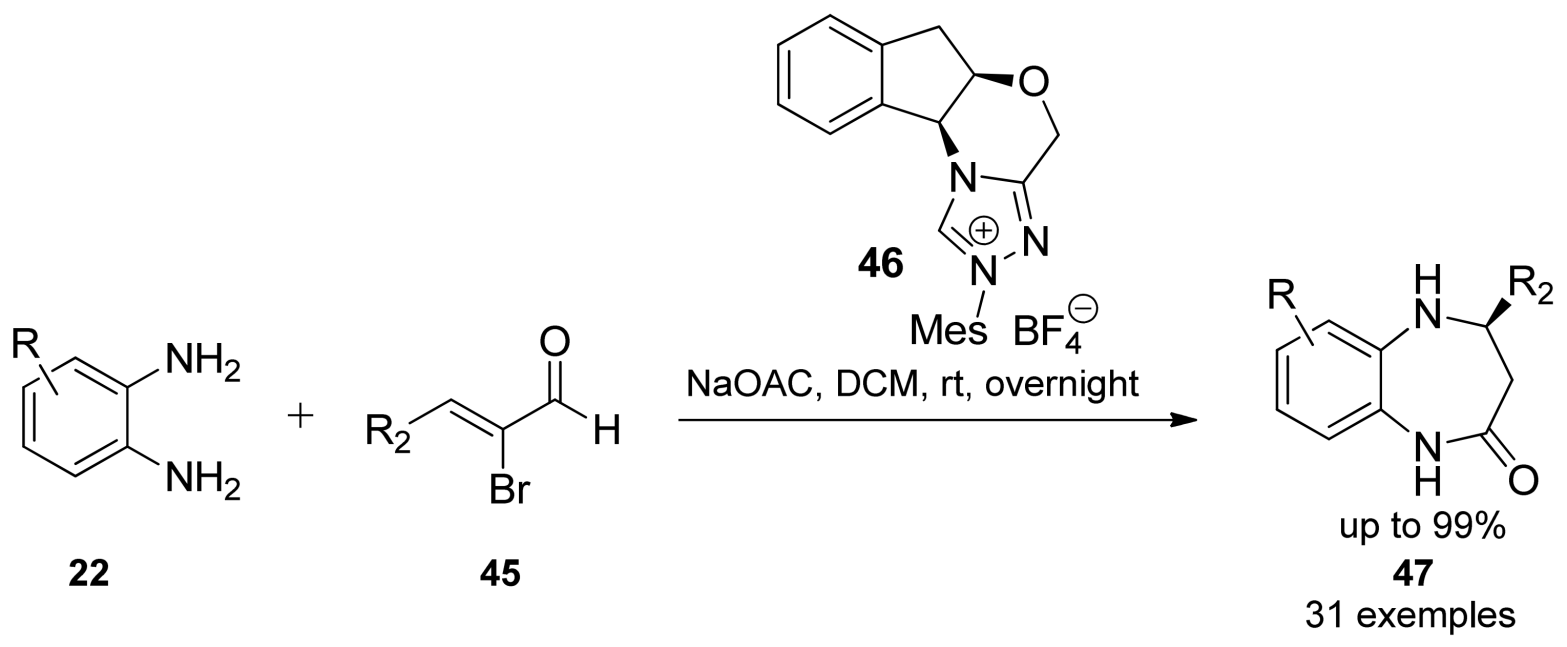
Enantioselective synthesis of *N*−*H*-free 1,5-benzodiazepin-2-ones via heterocyclic carbene-catalyzed [3+4] annulation.

Fang et al. [34] reported the development of an *N*-heterocyclic carbene-catalyzed formal [3+4] annulation between *α*,*β*-unsaturated acylazoliums and unprotected aryl 1,2-diamines (Scheme 13), enabling a direct and highly enantioselective synthesis of 4-aryl *N*−*H*-free 1,5-benzodiazepin-2-ones **47**. This approach provides rapid and efficient access to a broad range of enantioenriched target compounds from readily available starting materials.

#### 3.1.10. From Diamines and 2-Diazo-1,3-Diones

**Scheme 14:**
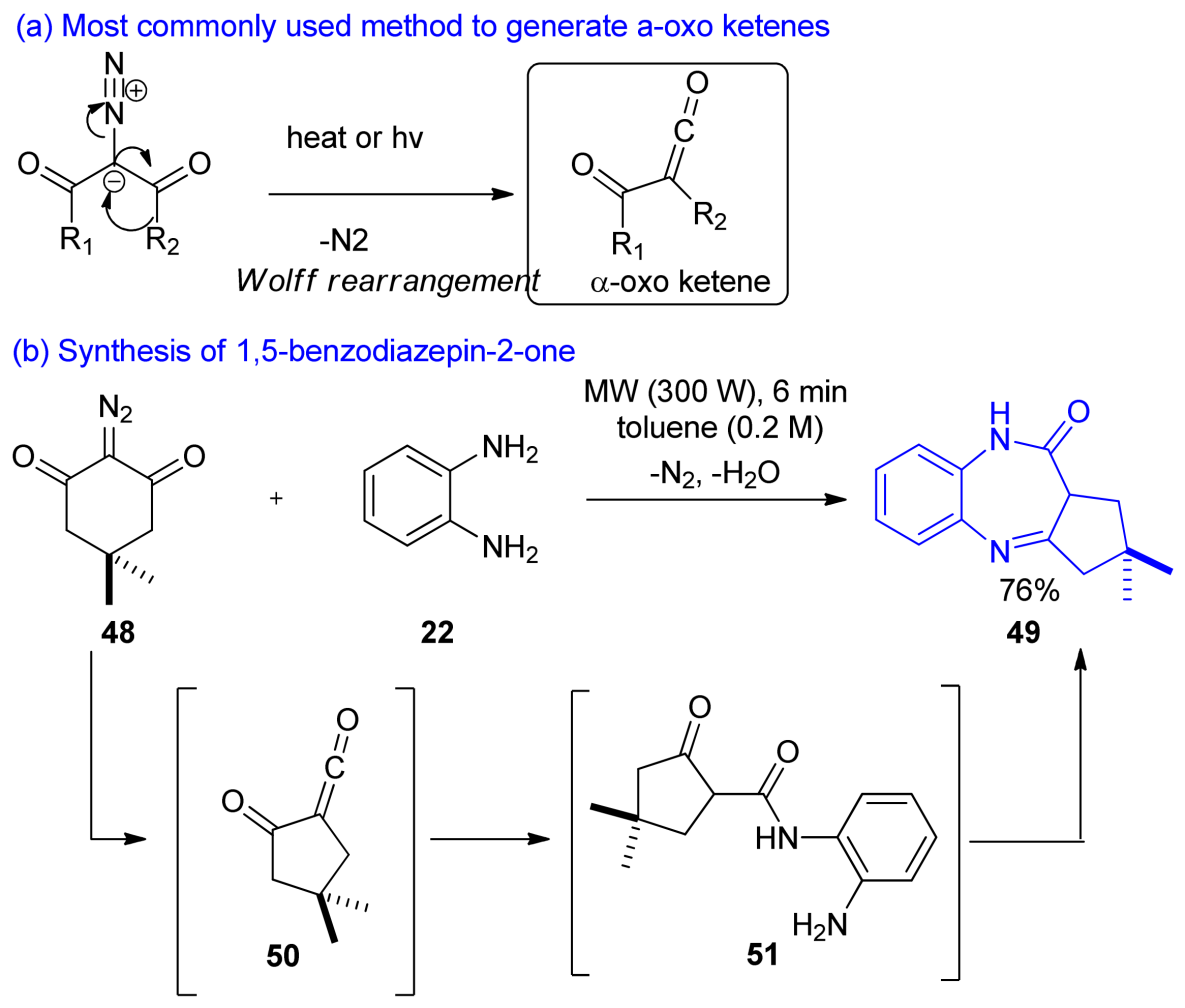
Microwave-assisted synthesis of cyclic 1,3-dihydro-2*H*-1,5-benzo-diazepin-2-ones via a domino reaction.

Juan-Carlos et al. [35] reported that the microwave-assisted reaction of 2-diazo-1,3-diones with *o*-phenylenediamines, conducted without any additives, furnished a series of bi- and tricyclic 1,3-dihydro-2*H*-1,5-benzodiazepin-2-ones **49** (Scheme 14) via a domino sequence comprising Wolff rearrangement, nucleophilic addition, and intramolecular imination. This methodology offers several advantages and represents a novel, complementary, and efficient synthetic approach to a valuable class of drug-like compounds.

#### 3.1.11. From Diamines and Münchnone

**Scheme 15:**
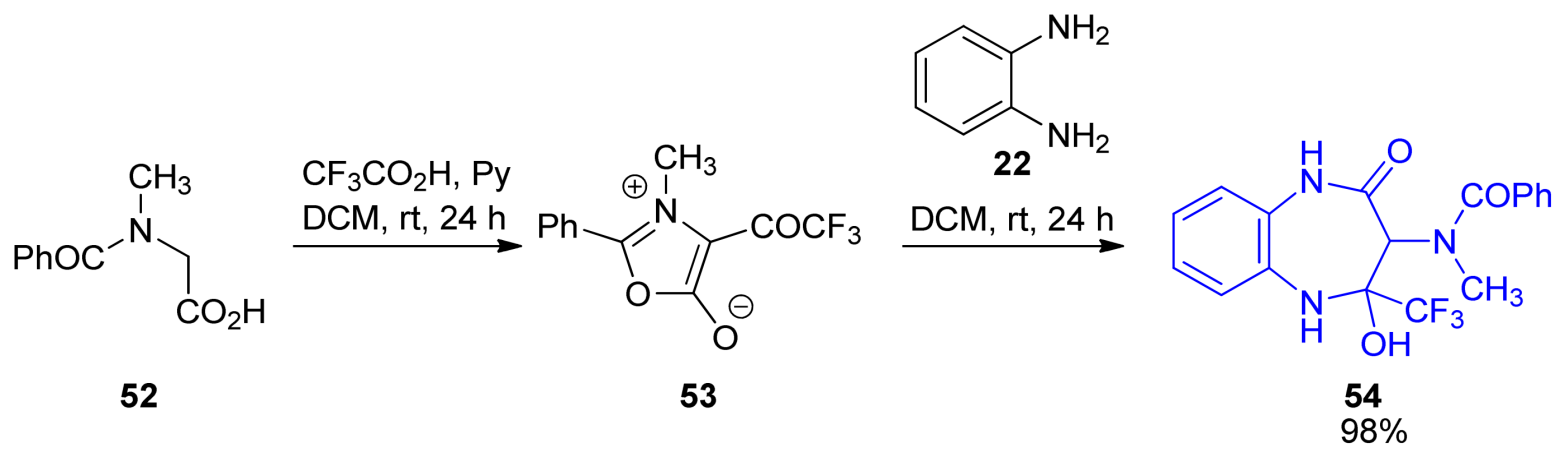
Synthesis of a 3-amino-1,5-benzodiazepin-2-one from a münchnone and phenylenediamine.

Kawase and co-workers [36] reported that mesoionic 4-trifluoroacetyl-1,3-oxazolium-5-olates (münchnones), readily prepared from glycine, condense with phenylenediamine in CH_2_Cl_2_ at 25 °C to afford 3-amino-1,5-benzodiazepin-2-one **54** in excellent yield (Scheme 15).

#### 3.1.12. From Amino Acids

**Scheme 16:**
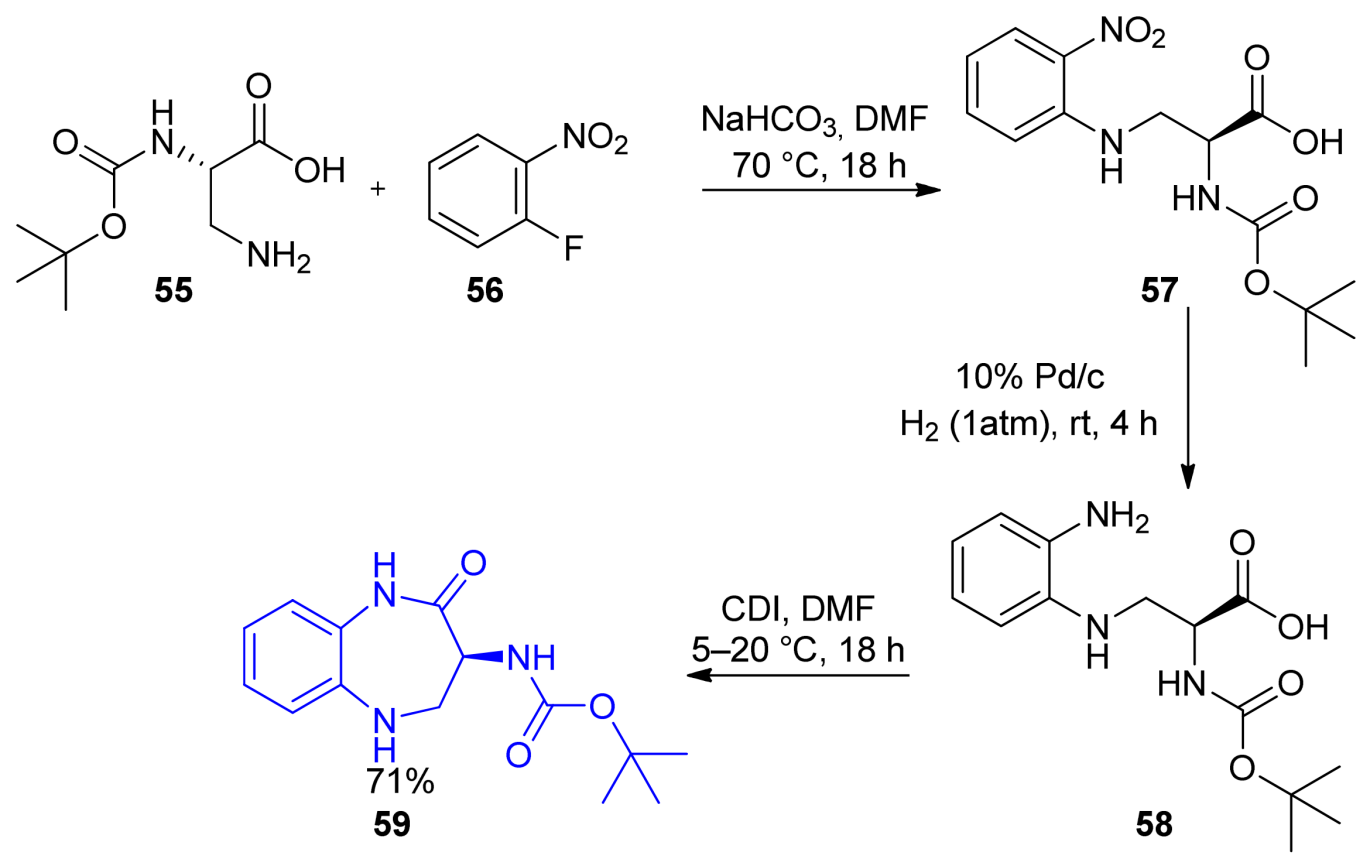
Synthesis of a 1,5-benzodiazepin-2-one derivative from an amino acid precursor.

Lauffer et al. [37] described an efficient three-step reaction sequence for the synthesis of 3-(tert-butoxycarbonylamino)-2-oxo-2,3,4,5-tetrahydro-1,5-benzodiazepine **59** from (2*S*)-3-amino-2-[(tert-butoxycarbonyl)amino]propanoic acid **55**, achieving an overall yield of 71% (Scheme 16).

### 3.2. From Other Ortho-Substituted Anilines

#### 3.2.1. From 2-Azetidinone and 2-Iodoaniline

**Scheme 17:**
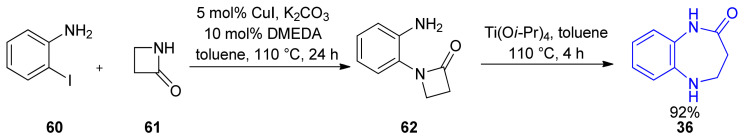
Synthesis of 1,5-benzodiazepin-2-one via a tandem Cu-catalyzed arylation/transamidation sequence.

In an interesting tandem process, Klapars and co-workers [38] described a Cu-catalyzed arylation of 2-azetidinone with 2-iodoaniline, followed by a Ti(OiPr)_4_-promoted transamidation (50 mol%) in toluene at 110 °C, which afforded the 1,5-benzodiazepin-2-one in 92% yield (Scheme 17).

#### 3.2.2. Cu-Catalyzed Ullmann-Type Intramolecular Arylation of Protected Amines

**Scheme 18:**
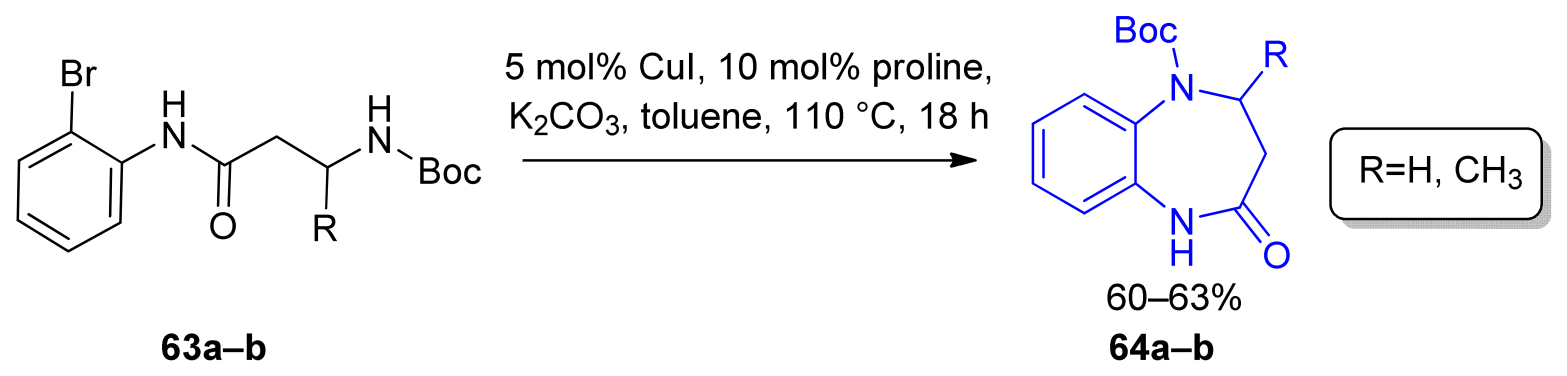
Synthesis of 1,5-benzodiazepine-2-ones via Cu-catalyzed intramolecular arylation.

Yang and co-workers [39] developed an efficient method for the preparation of 1,5-benzodiazepine-2-ones **64a–b** via a Cu-catalyzed Ullmann-type intramolecular arylation of protected amines. They found that tert-butoxycarbonyl (*N*-Boc) or *N*-2,6-diisopropylphenyl (*N*-Dipp) protection was essential for the cyclization (Scheme 18). Unprotected amines failed to react under these conditions. In contrast, aniline nucleophiles can undergo cyclization under Pd catalysis (Pd(OAc)_2_, BINAP, Cs_2_CO_3_, hot toluene) without protecting groups when using aryl iodides [40].

#### 3.2.3. Via Intramolecular Amidation for Ring Formation Using P(tBu)_3_ as the Ligand

**Scheme 19:**
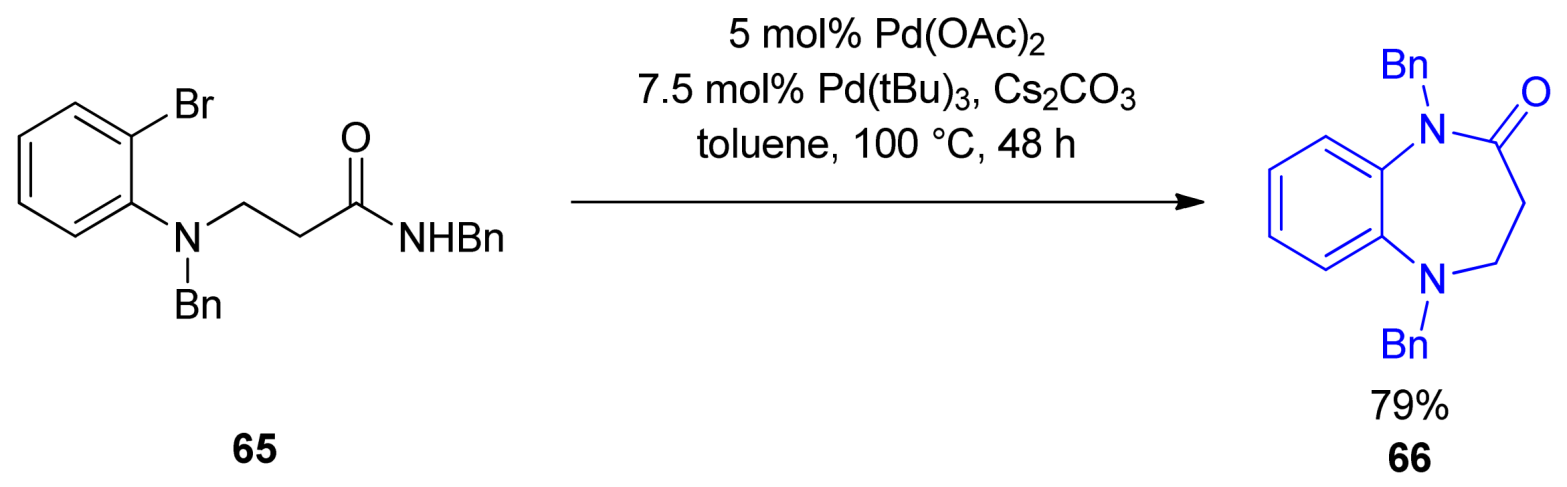
Synthesis of 1,5-dibenzyl-1,3,4,5-tetrahydro-1,5-benzodiazepin-2-one using P(tBu)_3_ as the ligand.

Cropper et al. [41] applied the Buchwald–Hartwig strategy to study the effect of Pd–ligands on intramolecular amidation for medium-sized ring formation. Using P(tBu)_3_ as the ligand, 1,5-dibenzyl-tetrahydro-1,5-benzodiazepin-2-one **66** was obtained from the corresponding benzylamide **65** in 79% yield. Compared with other ligands, the steric hindrance of P(tBu)_3_ was crucial for stabilizing the palladacycle and promoting product formation (Scheme 19).

#### 3.2.4. Via Intramolecular Cyclization of Aniline-Derived Intermediates Catalysed by Pd

**Scheme 20:**
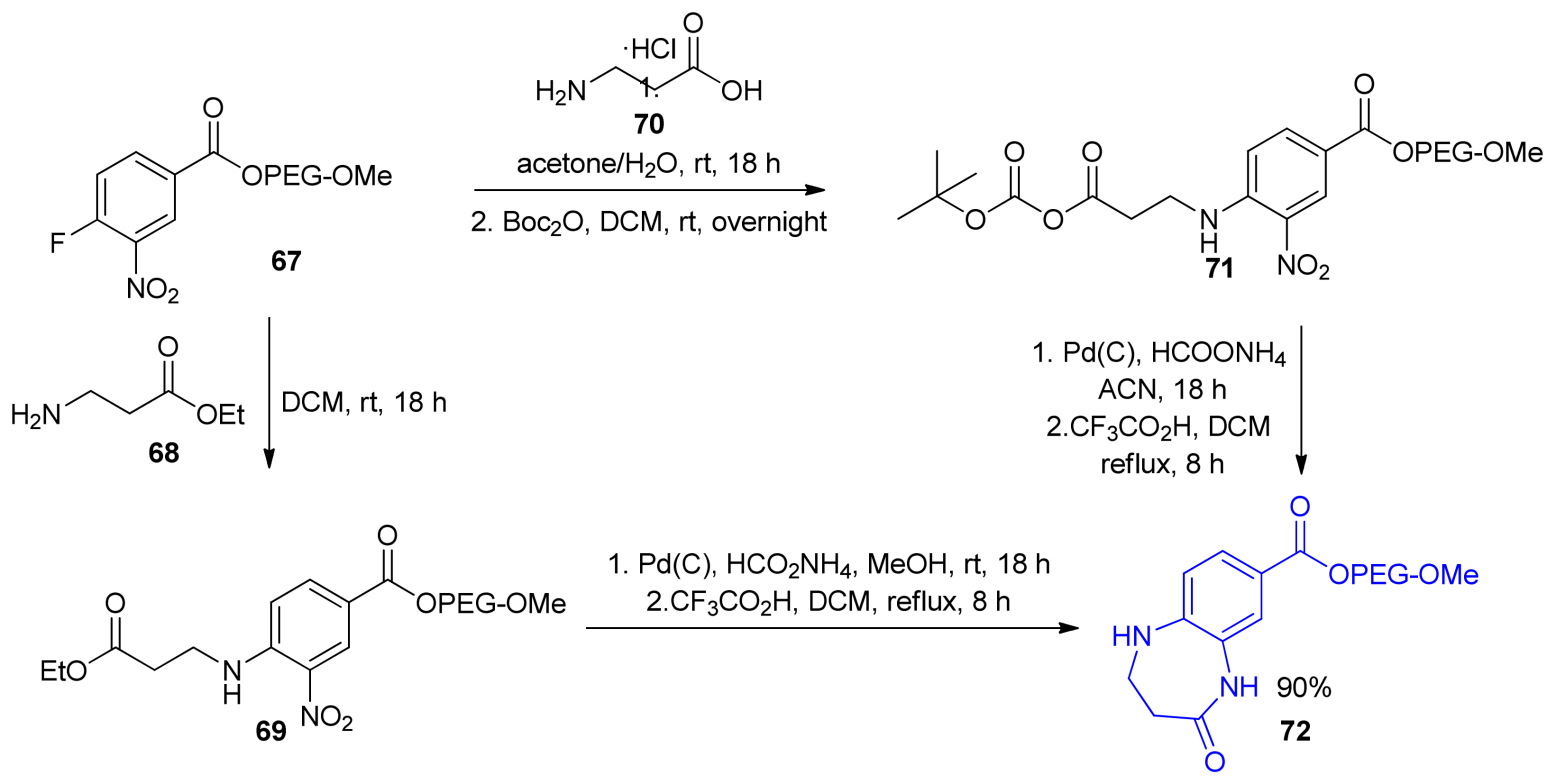
Synthesis of 1,5-benzodiazepin-2-one by intramolecular cyclization catalysed by Pd.

Wu’s group [42] reported that a soluble polymer-supported synthetic method offers a highly efficient route for the construction of biologically important 1,5-benzodiazepin-2-one **72**. Using this strategy, a library of *N*-substituted benzodiazepinones can be readily assembled through SNAr reaction, nitro group reduction, and one-pot cyclization (Scheme 20).

## 4. Heterocyclic Systems Derived from 1,5-Benzodiazepin-2-Ones

### 4.1. From 4-(2-Hydroxyphenyl) Derivatives

**Figure 6:**
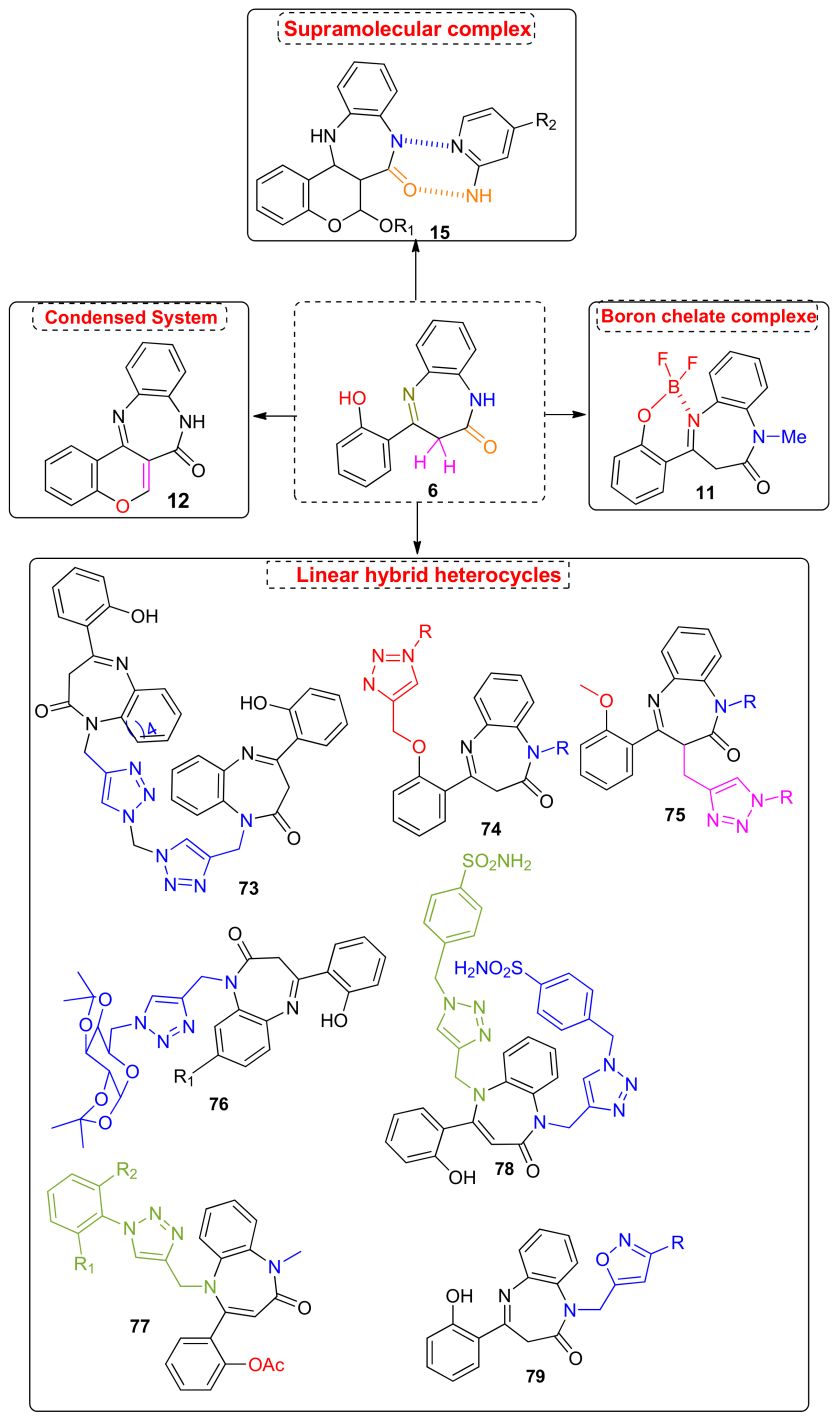
Representative diverse heterocyclic systems derived from 4-(2-hydroxyphenyl)-1,5-benzodiazepin-2-one.

The construction of polyheterocycles that integrate multiple privileged substructures within a single framework is a central strategy in modern medicinal chemistry [43]. In this context, 1,5-benzodiazepin-2-ones are particularly attractive due to their four reactive sites. Consequently, drug discovery efforts have focused on incorporating a wide variety of linear hybrid heterocycles such as 1,2,3-triazoles and isoxazoles—from 4-(2-hydroxyphenyl)-1,5-benzodiazepin-2-one **6** (Figure 6). For instance, a straightforward synthesis of 1,4-disubstituted 1,2,3-triazoles linked to 1,5-benzodiazepinones (compounds **73–78**) has been reported [11,22,44,45,46,47,48], achieved via a Cu(I)-catalyzed 1,3-dipolar alkyne–azide cycloaddition reaction (CuAAC) under either reflux or microwave (MW) conditions. Likewise, Sana’s group [49] developed a novel series of benzodiazepine-2-ones linked to 3,5-disubstituted isoxazoles **79**, which were synthesized by reacting *N*-propargylbenzodiazepines with various arylnitrile oxides through a 1,3-dipolar cycloaddition catalyzed by Cu(I) under both conventional and microwave irradiation conditions.

In the pursuit of new polycyclic heterocycles of biological interest derived from 4-hydroxycoumarin, Rafik and co-workers [18] prepared benzopyrano-1,5-benzodiazepin-2-one **12** via the reaction of *N,N*-dimethylformamide dimethylacetal (DMFDMA) with 4-(2-hydroxyphenyl)-1,5-benzodiazepin-2-one. Additionally, Hasan et al. achieved boron complexation using BF_3_·Et_2_O, affording the corresponding boron chelate complex **11** [12]. Further advances include the design of self-assembled systems, such as *o*-alkylbenzochromeno-1,5-benzodiazepinones interacting with 2-aminopyridine through hydrogen bonding (supramolecular complex **15**) [13], as described by Asma’s group [16]. Collectively, these strategies expand the structural diversity of the scaffold and enhance its therapeutic potential.

### 4.2. Condensed Systems from 4-Phenyl Derivatives

**Scheme 21:**
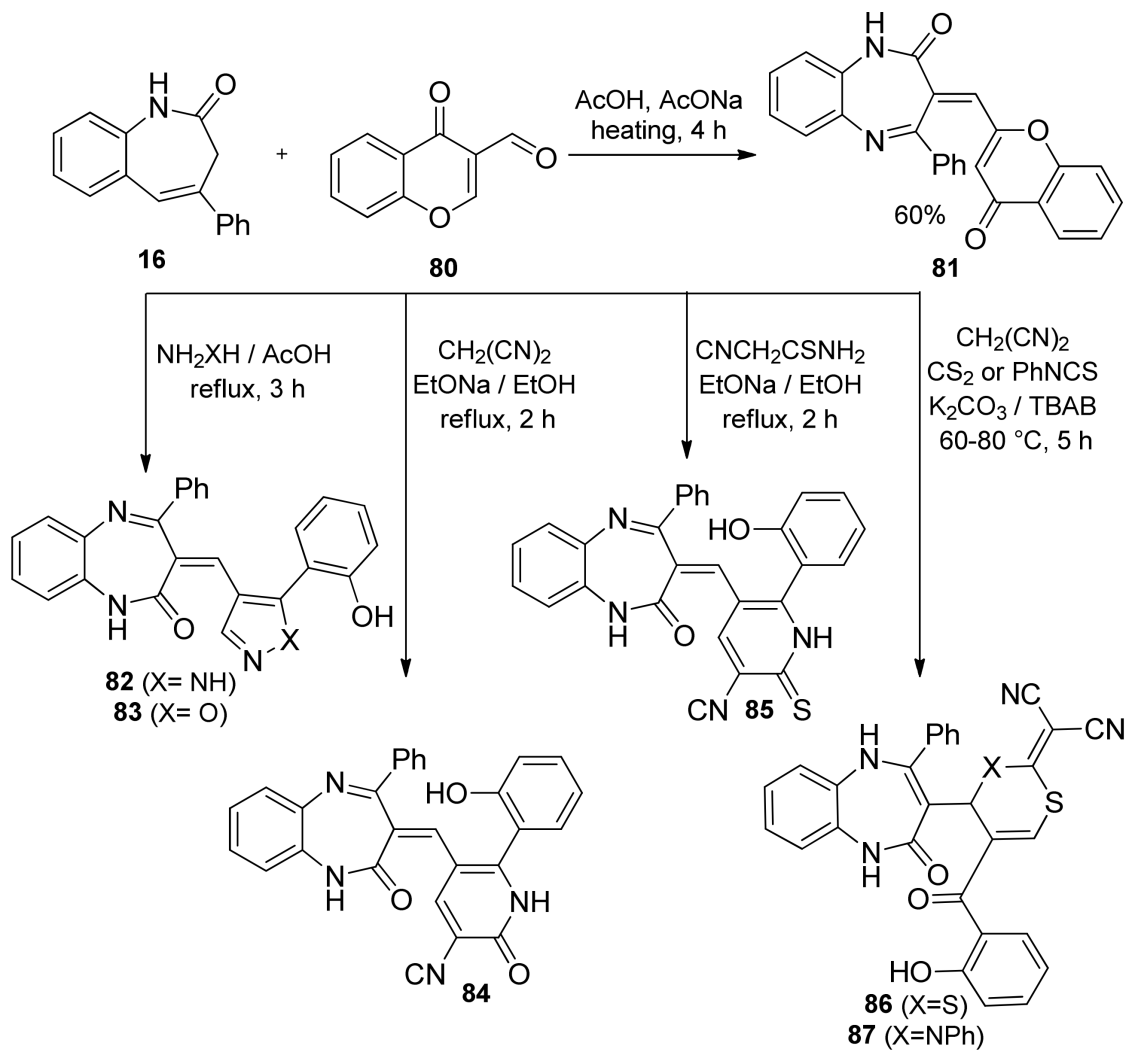
Some condensed heterocycles synthesized from 4-Phenyl-1,5-benzodiazepin-2-one.

The introduction of a 3-chromenylmethylene moiety into active methylene-containing heterocycles generates a reactive enone system capable of nucleophilic attack, enabling ring-opening/closure to yield novel heterocycles. Hanan and co-workers [50,51] used this approach to synthesize 1,5-benzodiazepin-2-one derivatives (Scheme 21) from 3-((4-oxo-4*H*-chromen-3-yl)methylene)-4-phenyl-1*H*-1,5-benzodiazepin-2(3*H*)-one **81**. Reaction with hydrazine hydrate or hydroxylamine hydrochloride in acetic acid afforded pyrazolyl and isoxazolyl derivatives (**82**, **83**). With malononitrile and sodium ethoxide, compound **81** gave pyridinyl derivative **84**. Reaction with cyanothioacetamide led to chromone ring-opening and pyridine ring-closure, yielding compound **83**. Under phase-transfer conditions (K_2_CO_3_/TBAB), compound **81** reacted with CS_2_/malononitrile or Ph-NCS/malononitrile to give 1,3-dithiinylidene (**86**) or 1,3-thiazinylidene (**87**) malononitrile derivatives.

**Scheme 22:**
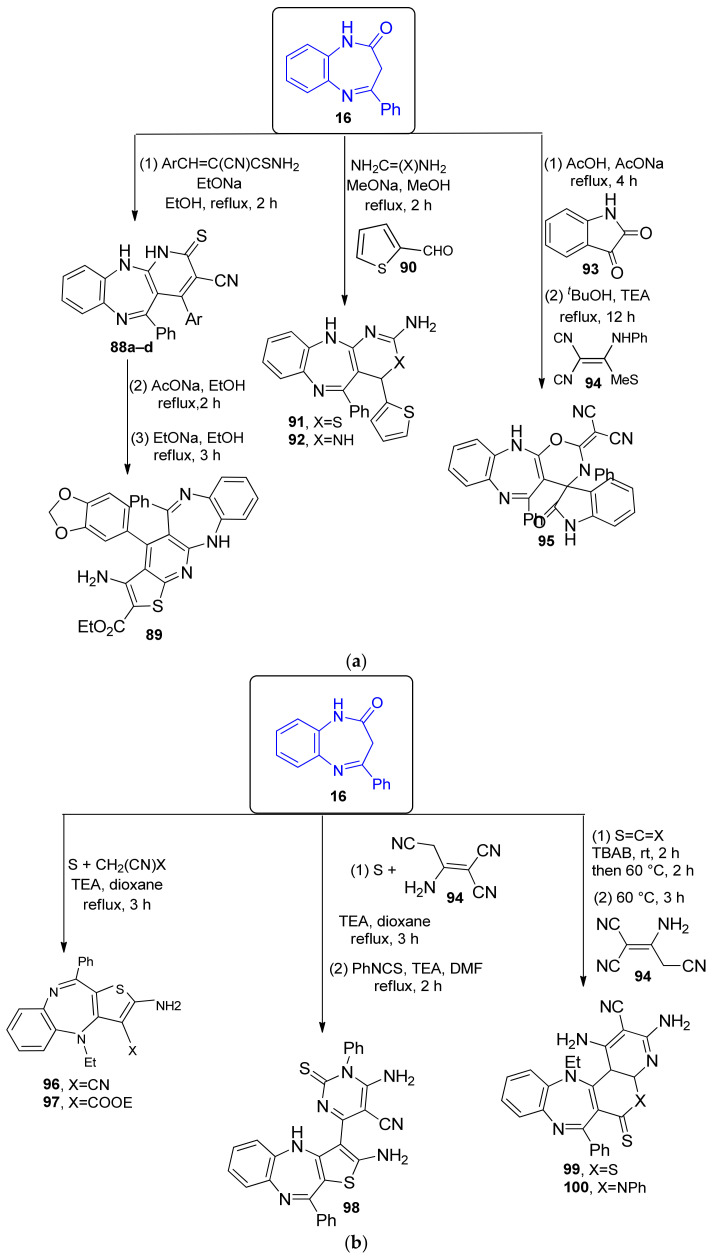
(**a**) Fused heterocycles derived directly from 1,5-benzodiazepin-2-one. (**b**) Fused heterocycles derived directly from 1,5-benzodiazepin-2-one.

In the same context, Khodairy and co-workers [52] investigated several synthetic routes (Scheme 22a,b) for the direct preparation of fused heterocycles from the 1,5-benzodiazepin-2-one scaffold. For example, 1,3-di*H*-4-phenyl-1,5-benzodiazepin-2-one **16** reacts with various arylidenecyanothioacetamides to give 3-substituted derivatives, which upon cyclization with sodium ethoxide furnish 3-cyano-pyrido-1,5-benzodiazepine-2-thiones (**88a–d**). Subsequent reactions with halogenated compounds afford S-alkylated intermediates, which can be further cyclized to thieno[3′,2′:5,6]pyrido-1,5-benzodiazepine **89**.

Compound **16** also reacts with thiophen-2-carbaldehyde and thiourea or guanidine to yield thiazino-1,5-benzodiazepines **91** and pyrimido[4,5-*b*]-1,5-benzodiazepine **92**. With isatin, followed by condensation with malononitrile derivatives, it affords oxazine-fused benzodiazepine **95**. Reaction with sulfur and malononitrile or ethyl cyanoacetate gives thieno[3,2-*b*]benzodiazepines (**96**, **97**), while treatment with 1,1,3-tricyano-2-aminoprop-1-ene and isothiocyanates provides pyrimidinylthieno[3,2-*b*]benzodiazepine **98**. Finally, under phase-transfer catalysis (PTC) conditions, compound **16** undergoes condensation with CS_2_ or PhNCS to yield pyridothiopyrano[4,3-*b*]-1,5-benzodiazepine **99** and benzodiazepino[6,5-*c*][1,8]naphthyridines **100**.

**Scheme 23:**
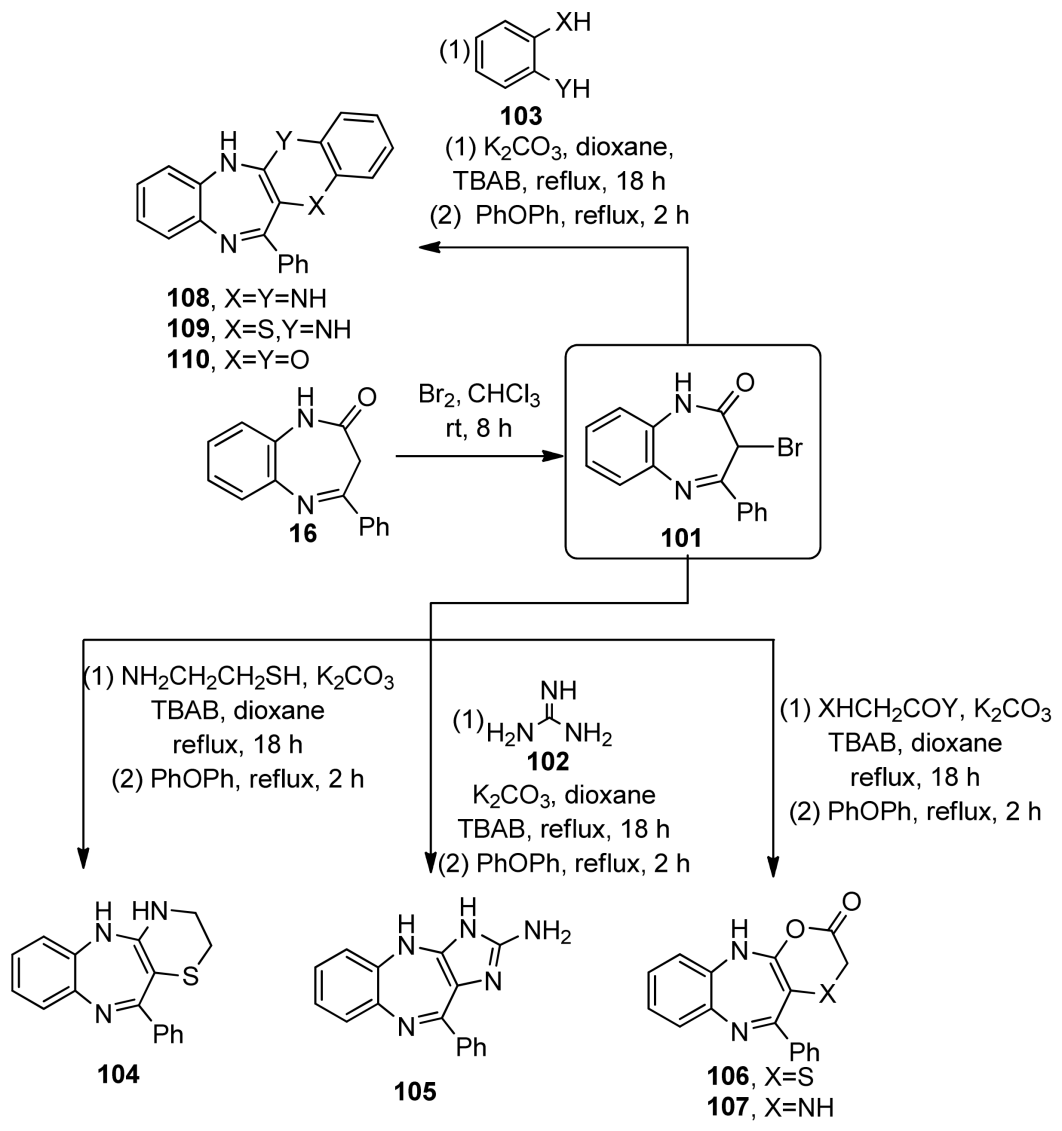
Fused heterocycles from brominated 4-phenyl-1,5-benzodiazepine-2-one.

The notable pharmacological activities of 1,5-benzodiazepin-2-ones [53,54] motivated AbdelGhany and co-workers [55] to explore the synthesis of fused heterocycles derived from the 1,5-benzodiazepin-2-one framework (Scheme 23). In their study, 3-bromo-1,3-di*H*-1,5-benzodiazepin-2-one **101** was obtained by bromination of 1,3-di*H*-4-phenyl-1,5-benzodiazepin-2-one **16** with an equimolar amount of bromine in chloroform. This brominated intermediate served as a versatile precursor for 4-phenyl-3-substituted-1*H*-1,5-benzodiazepin-2-ones. Subsequent reactions of compound **101** with cystamine hydrochloride, guanidine hydrochloride, ethyl mercaptoacetate, ethyl glycinate hydrochloride, *o*-phenylenediamine, *o*-aminothio-phenol, and catechol afforded the corresponding 1,5-benzodiazepin-2-one derivatives (compounds **104–110**) under phase-transfer catalytic conditions. The system employed dioxane as the organic phase, potassium carbonate as the base, and tetrabutylammonium bromide (TBAB) as the catalyst. The mechanism involves proton abstraction at the solid carbonate surface to form an anion, followed by its transfer as an ion pair into the organic phase, where nucleophilic substitution occurs in the order of reactivity: –S > –N > –O.

**Scheme 24:**
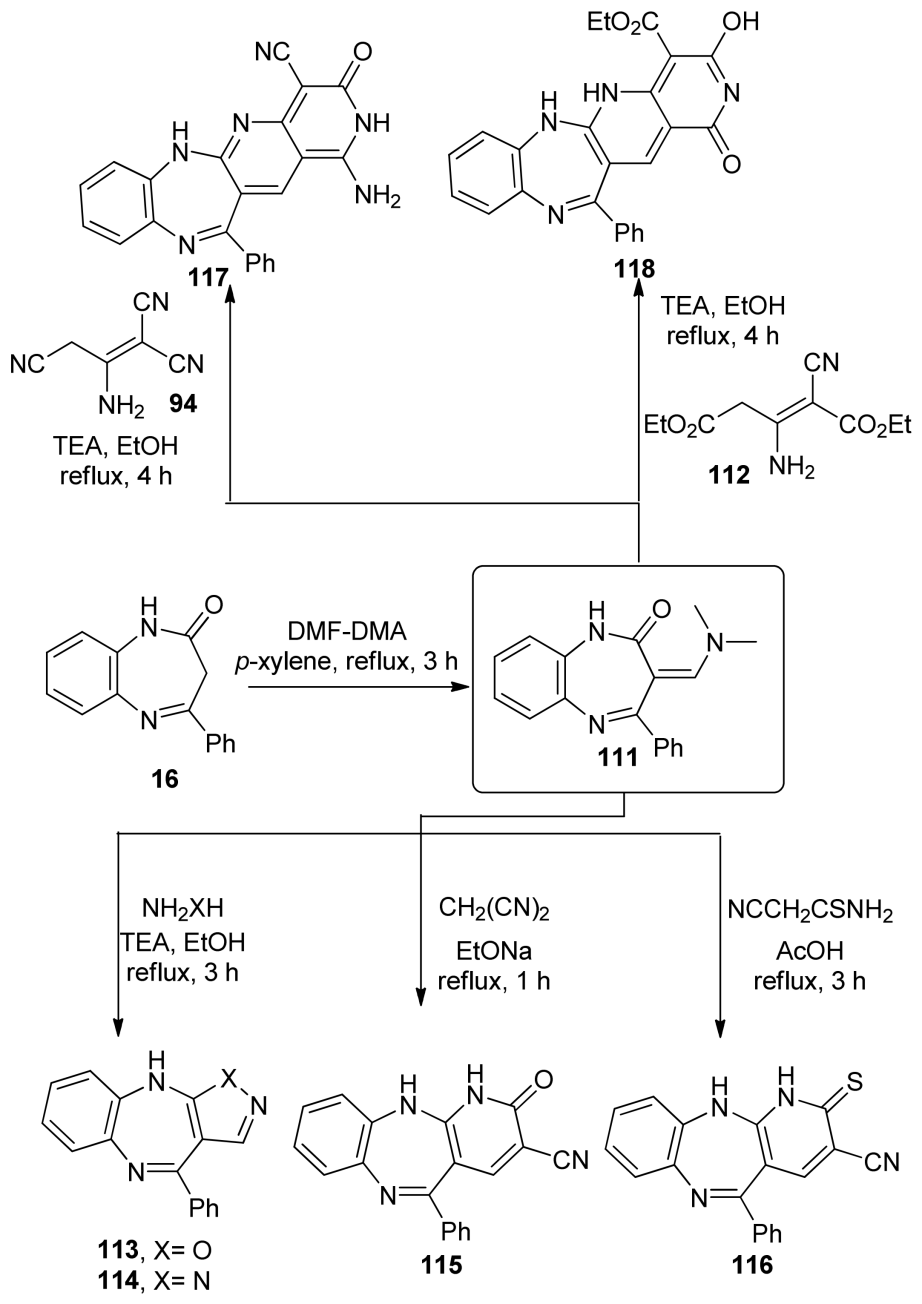
Fused heterocycles from an enaminone precursor.

Enaminones have been widely employed as versatile building blocks in the organic synthesis of polyfunctionally substituted heterocycles [56,57,58,59]. In this context, Khodairy and co-workers [60] developed the use of 3-dimethylaminomethylene-4-phenyl-1*H*-1,5-benzodiazepin-2-one **111** as a versatile precursor for the synthesis of pyrazole, isoxazole, and pyridine derivatives with potential medicinal interest (Scheme 24). Compound **111** was obtained by reacting 1,3-dihydro-4-phenyl-1,5-benzodiazepin-2-one with DMF-DMA under reflux in p-xylene. Treatment of enaminone **111** with hydrazine or hydroxylamine afforded pyrazolo[3,4-*b*]- and isoxazolo[5,4-*b*]-1,5-benzodiazepines (**113**, **114**). Reactions with malononitrile (NaOEt) or cyanothioacetamide (AcOH) yielded pyrido[2,3-b]-1,5-benzo-diazepin-2-one **115** and its thione analogue **116**. The formation of compounds **117** and **118** likely proceeds via nucleophilic attack of the active methylene on the olefinic bond of **111**, elimination of dimethylamine, intramolecular condensation, partial cyano hydrolysis, and subsequent cyclization.

**Scheme 25:**
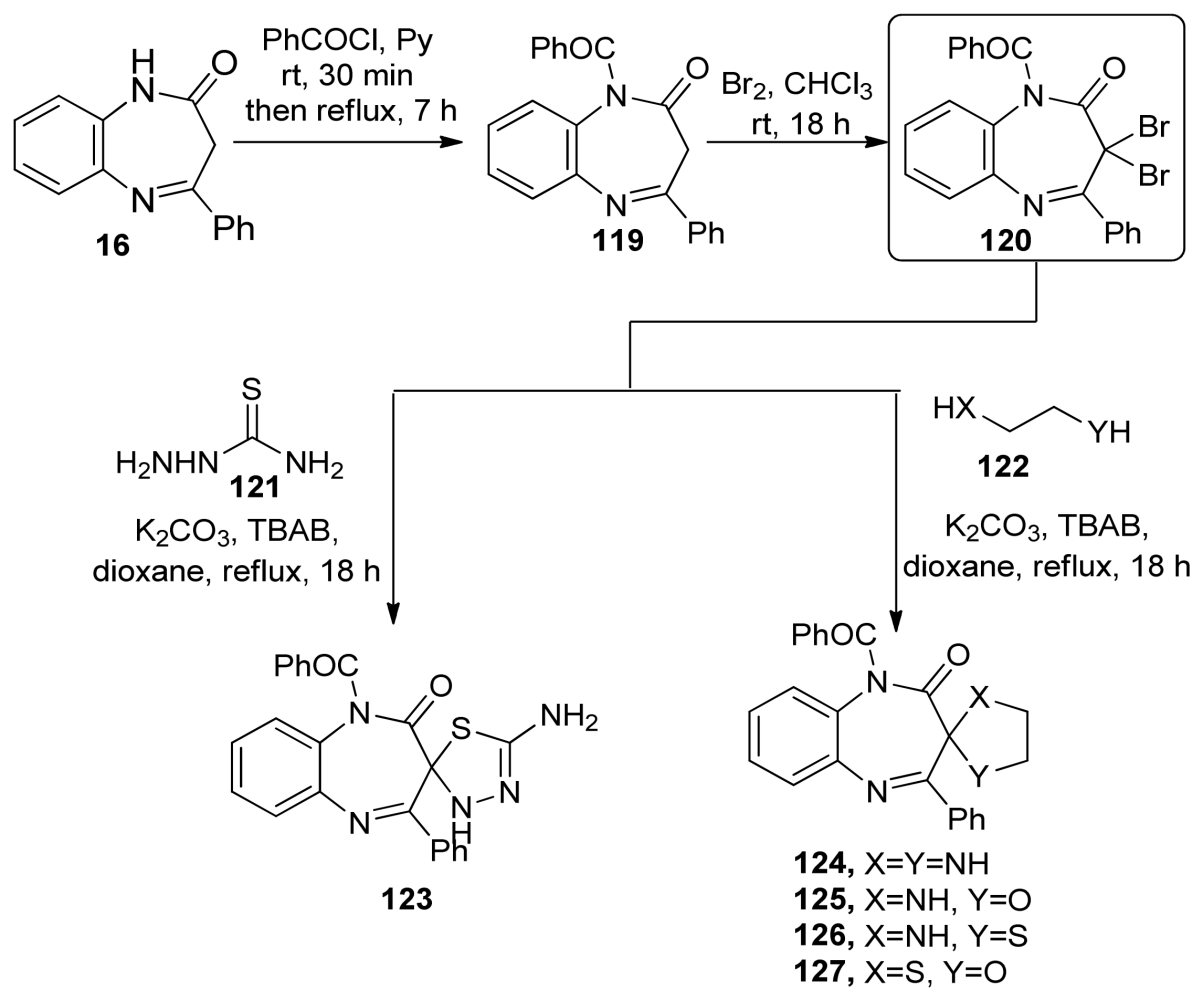
Spiro-heterocyclic systems from a dibrominated 1,5-benzodiazepin-2-one.

On the other hand, Abdel-Ghany et al. [55] reported the synthesis of spiro-heterocyclic systems from the 1,5-benzodiazepin-2-one scaffold (Scheme 25). Compound **16** was first treated with benzoyl chloride in pyridine, which acted as both solvent and catalyst, to give 1-benzoyl-4-phenyl-3*H*-1,5-benzodiazepin-2-one **119**. Subsequent bromination of compound **119** with bromine (1:2 molar ratio) in chloroform at room temperature afforded 1-benzoyl-3,3-dibromo-4-phenyl-1,5-benzodiazepin-2-one **120**, a key intermediate for spiro-heterocycle construction. Under phase-transfer catalytic conditions [dioxane/K_2_CO_3_/TBAB], reaction of compound **120** with ethylenediamine, ethanolamine, cystamine hydrochloride, 2-mercaptoethanol, or thiosemicarbazide furnished the corresponding spiro(1,5)benzodiazepin-2-one derivatives (compounds **123–127**).

**Scheme 26:**
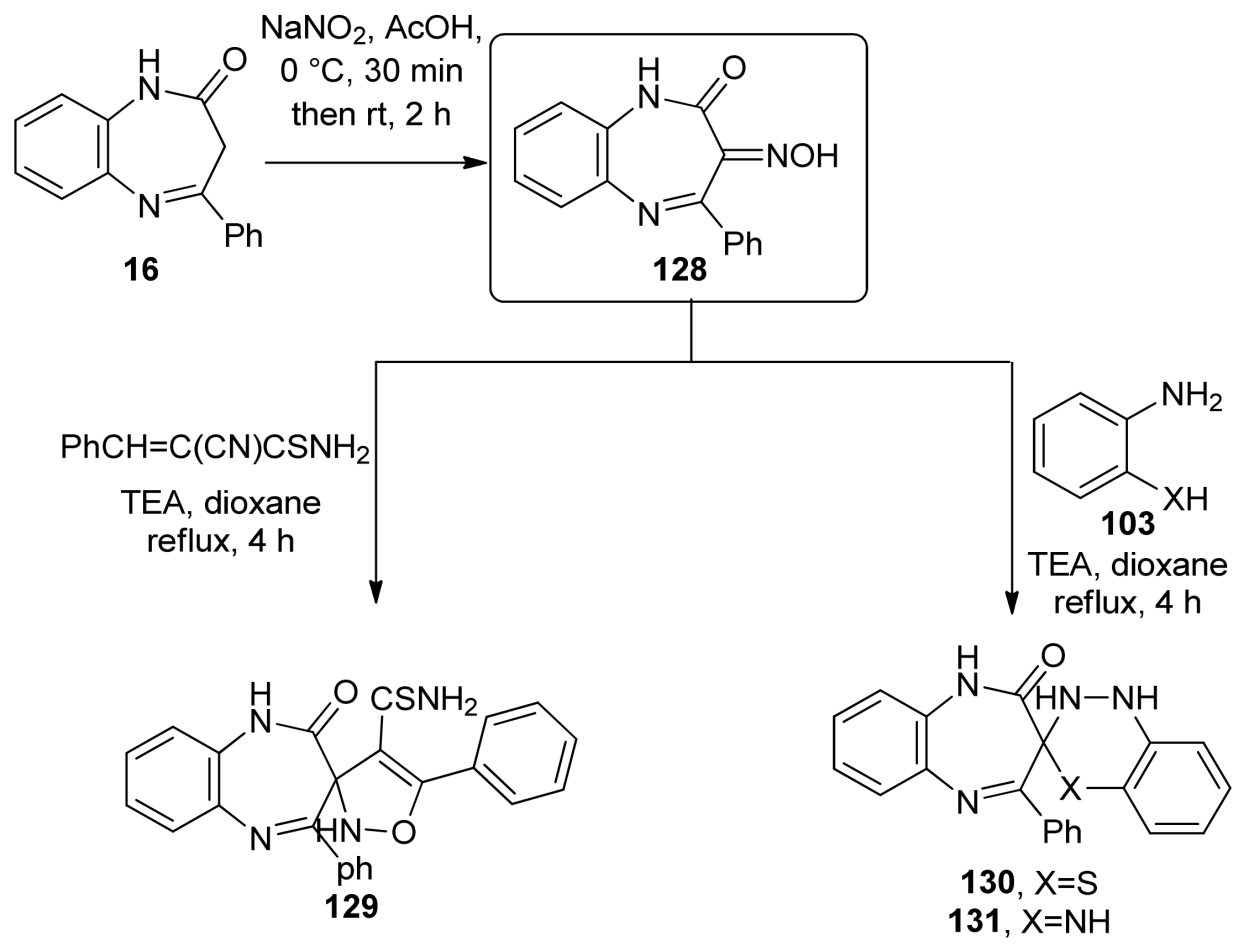
Spiro 1,5-benzodiazepine-2-ones starting by nitrosation of **16**.

**Scheme 27:**
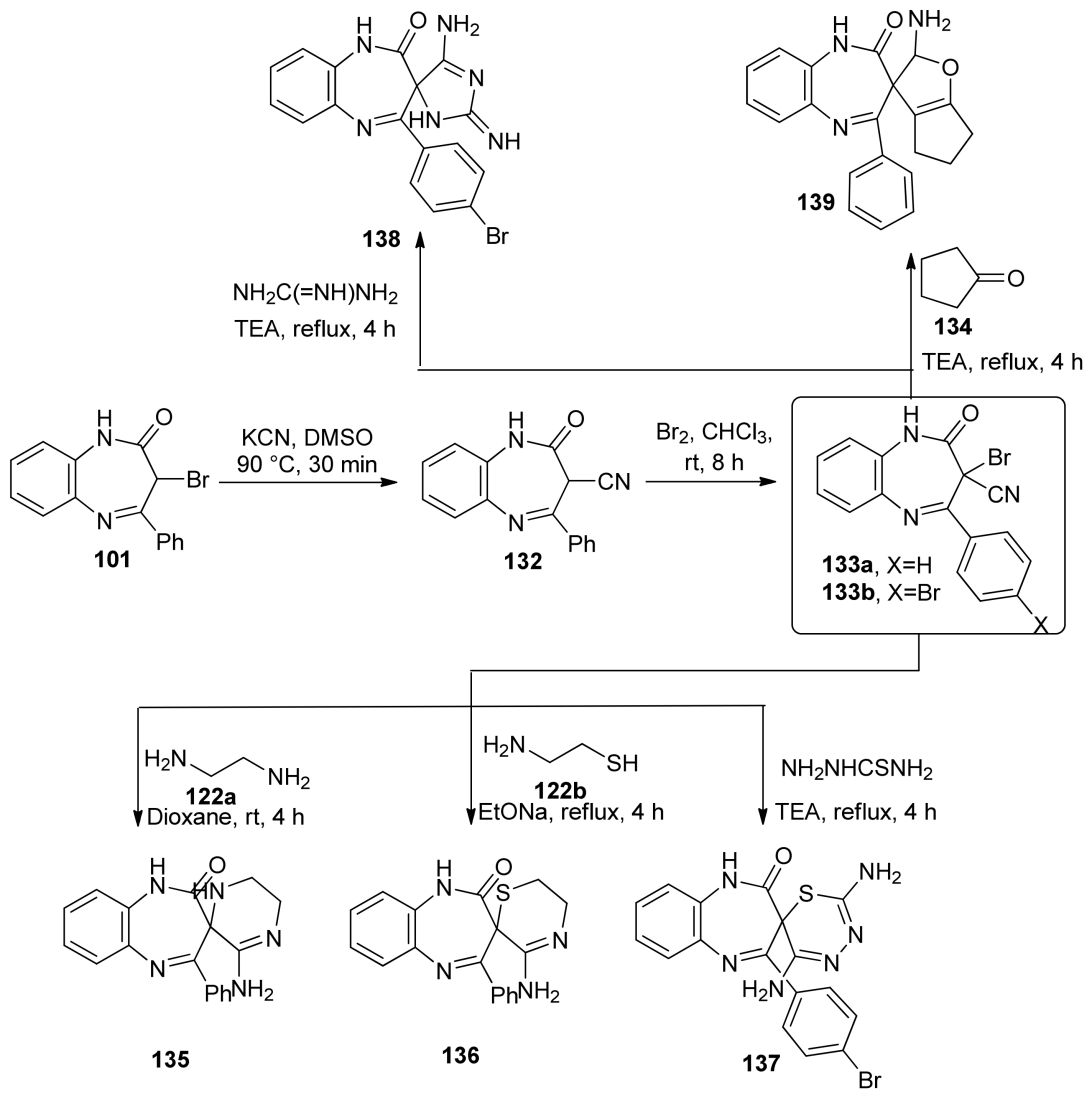
Spiro 1,5-benzodiazepine-2-ones starting by cyanation of **101**.

**Scheme 28:**
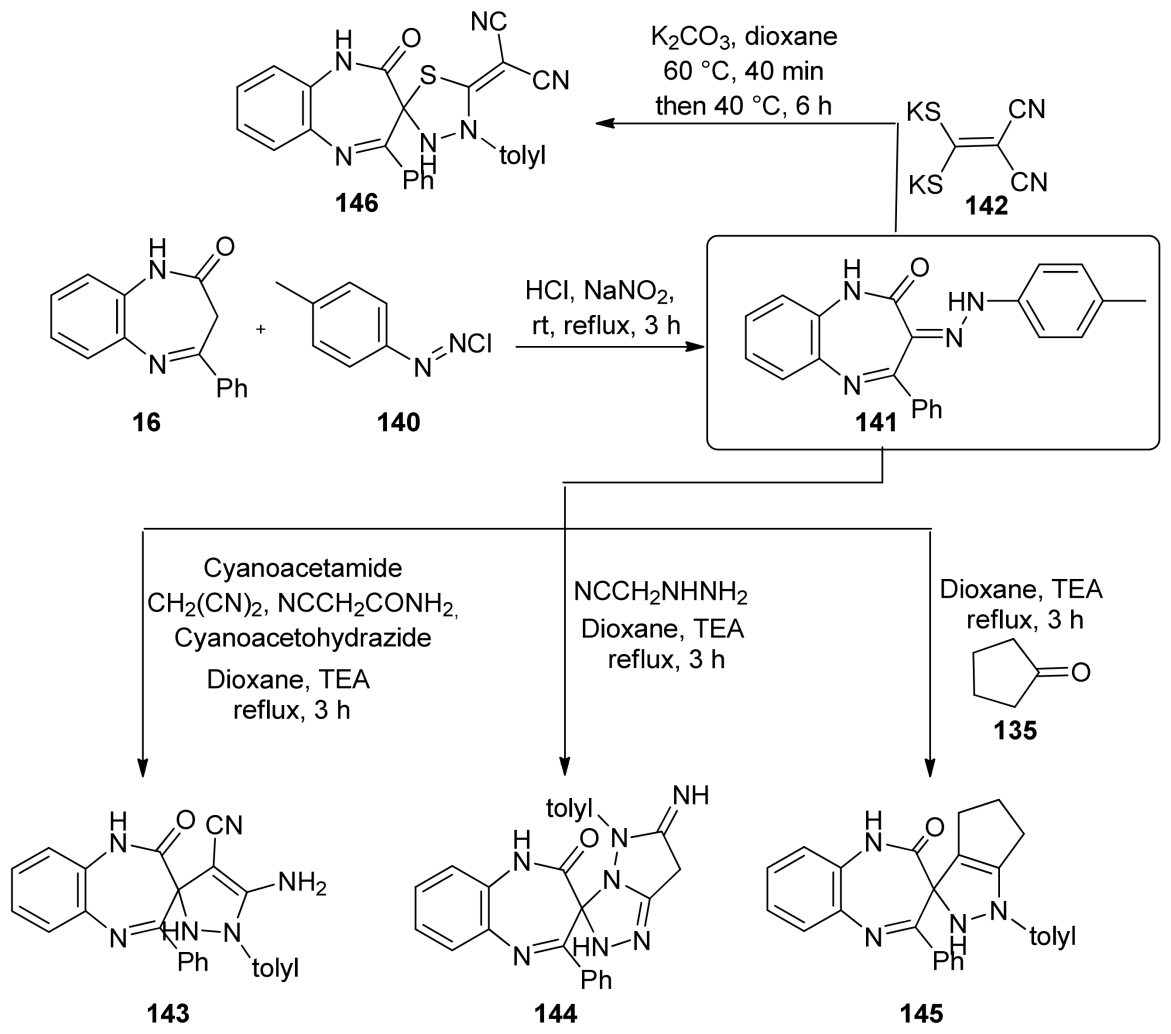
Spiro heterocycles starting with the formation of 3-(p-tolylazo) benzodiazepine-2-one **141**.

Furthermore, El-Sayed and co-workers [61] focused on the synthesis of fused and spiro-1,5-benzodiazepin-2-one derivatives. 3-Oximino-4-phenyl-1*H*-1,5-benzodiazepin-2-one (compound **128,** Scheme 26) was obtained and reacted with malononitrile, arylidenenitriles, and bidentate reagents to afford spiro-isoxazolo, thiadiazino, and triazino derivatives (compounds **129–131**). Bromination of 3-cyano-4-phenyl-1*H*-1,5-benzodiazepin-2-one **130** furnished compounds **133a–b** (Scheme 27), which served as key intermediates for the synthesis of spiro-piperazine, quinoxaline, thiazine, thiadiazine, imidazole, and cyclopentafuran analogues (compounds **135–139**). Furthermore, compound **16** (Scheme 28) was converted into 3-(*p*-tolylazo)-4-phenyl-1*H*-1,5-benzodiazepin-2-one **141**, which upon reaction with nitriles, cyclopentanone, or *S*,*S*-acetal derivatives afforded spiro-pyrazole, cyclopentapyrazole, and thiadiazole analogues (compounds **143–146**).

### 4.3. Hybrid Heterocycles from 4-Methyl Derivatives

**Scheme 29:**
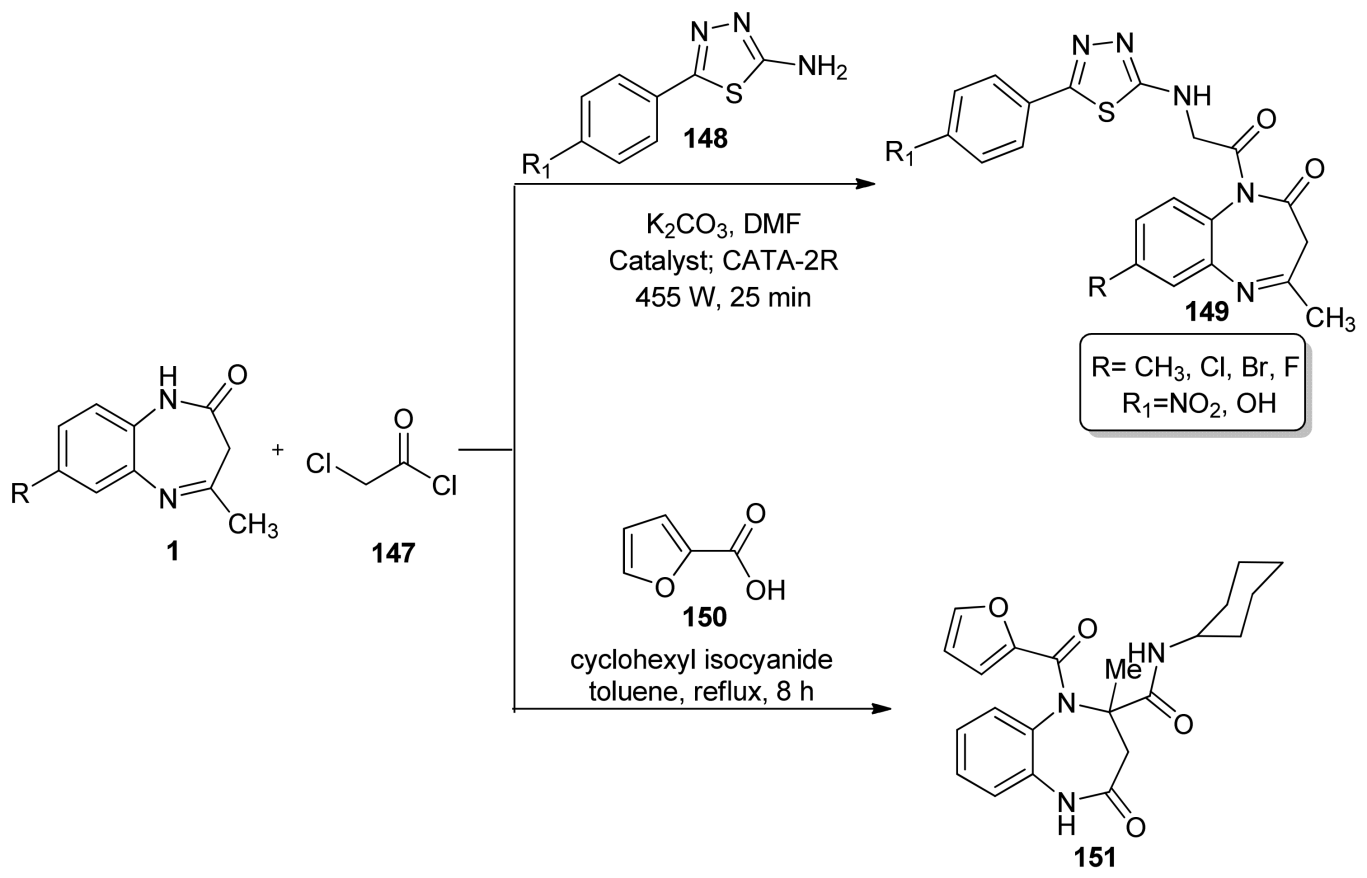
Examples of hybrid heterocycles synthesized from 4-methyl-1,5-benzodiazepin-2-one.

The pharmacological properties, potency, and pharmacokinetic behaviour of 1,5-benzodiazepin-2-one derivatives are influenced by their various substituents. Rishipathak et al. [7] described the synthesis of several 4-substituted-phenyl-1,3,4-thiadiazole-linked 1,5-benzodiazepin-2-one derivatives **149**. This was achieved through the initial formation of an N^1^-chloroacetyl-1,5-benzodiazepine-2-one derivative, followed by reaction with 2-amino-5-(4-substituted phenyl)-1,3,4-thiadiazole using anhydrous potassium carbonate in DMF under microwave irradiation (Scheme 29). In addition, Nasrin and co-workers [10] developed an efficient Ugi-based synthetic route to hybrid 1,5-benzodiazepin-2-ones **151** bearing a peptoid backbone. This method employs o-phenylenediamine and diketene in place of the conventional amine and aldehyde components, in combination with a carboxylic acid and cyclohexyl isocyanide. The reaction proceeds smoothly under neutral conditions without bases or catalysts, affords high yields, and allows straightforward product purification, highlighting its practicality and effectiveness.

## 5. Ring Transformations and Rearrangements

**Scheme 30:**
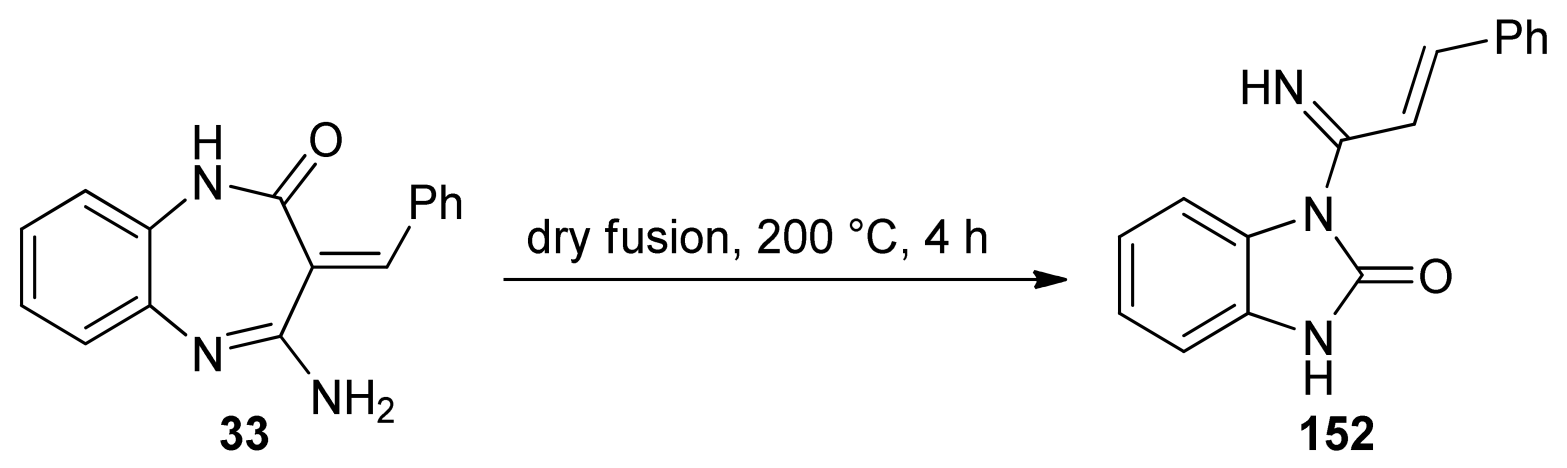
Thermal ring contraction of 1,5-benzodiazepin-2-one **33** into an *N*-alkenylimidazolone.

Hanaa’s group [28] reported the synthesis of a new family of 1,5-benzodiazepin-2-ones and demonstrated an intriguing finding (Scheme 30): compound **152** could be obtained exclusively by the dry fusion of **33**, via thermal contraction of the diazepinone ring into an imidazolone framework. The mechanism of this diazepinone-to-imidazolone transformation was proposed and validated through DFT calculations. The results revealed that a precise proton transfer from the primary amino hydrogen of **33** acts as the key driving force in this transformation.

**Scheme 31:**
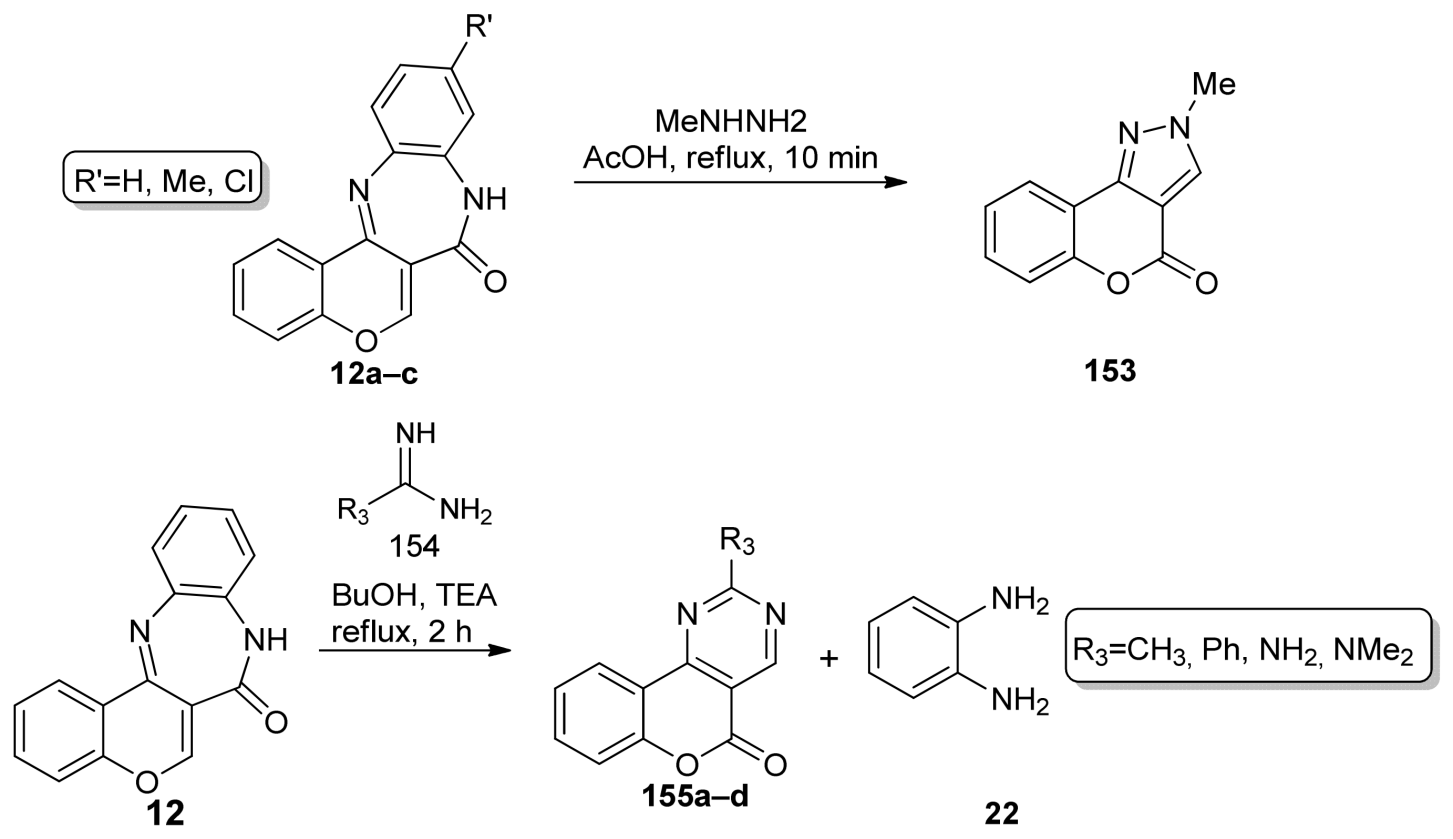
Reactivity of 1,5-benzodiazepin-2-ones towards *N*-binucleophiles.

Belsem and co-workers [62] investigated the reactivity of these compounds towards nitrogen-based binucleophiles. With non-aromatic hydrazines, the reaction carried out in acidic medium led exclusively to the unexpected [1]benzopyrano[4,3-*c*]pyrazol-4-ones **153**. To extend this strategy, a series of differently substituted amidines was employed. Their condensation with an equimolar amount of benzopyrano-benzodiazepinone **12** under mild conditions (refluxing butanol in the presence of triethylamine) exclusively afforded the expected 5-*H*-[1]benzopyrano[4,3-*d*]pyrimidin-5-ones (**155a–d**) (Scheme 31).

**Scheme 32:**
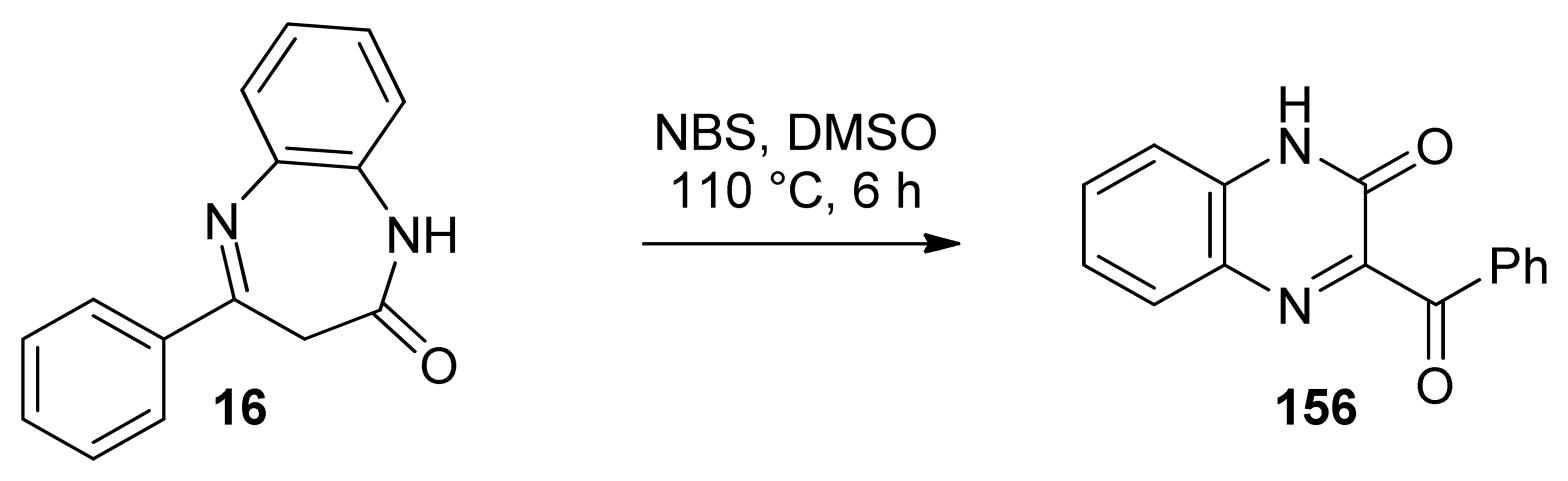
Oxidative ring contraction of a 1,5-benzodiazepin-2-one to 3-benzoylquinoxalin-2-one.

Hasan et al. [63] reported a novel and practical synthesis of 3-benzoylquinoxalin-2-one **156** starting from 4-phenyl-1,5-benzodiazepin-2-one **16** (Scheme 32), which can be obtained in only two steps from commercially available starting materials. The transformation was carried out in the presence of *N*-bromosuccinimide (NBS) in DMSO, which acted as both the solvent and the oxidant.

**Scheme 33:**
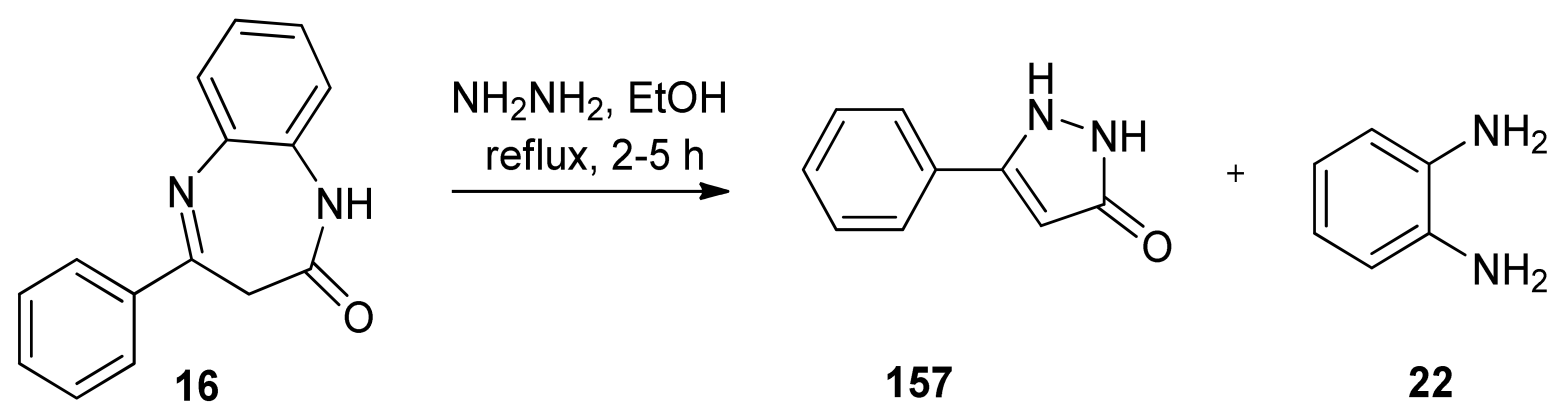
Hydrazinolysis of 4-phenyl-1,5-benzodiazepin-2-one.

Sergiy and co-workers [64] used density functional theory (DFT) to investigate the mechanism of hydrazinolysis of 4-phenyl-1,5-benzodiazepin-2-one (Scheme 33). The study showed that the reaction begins with the addition of a hydrazine molecule to the azomethine bond. This is followed by cyclization, leading to the formation of a pyrazole ring, and subsequent opening of the diazepine ring. Finally, the elimination of o-phenylenediamine yields the pyrazolone product **157** as the main product.

**Scheme 34:**
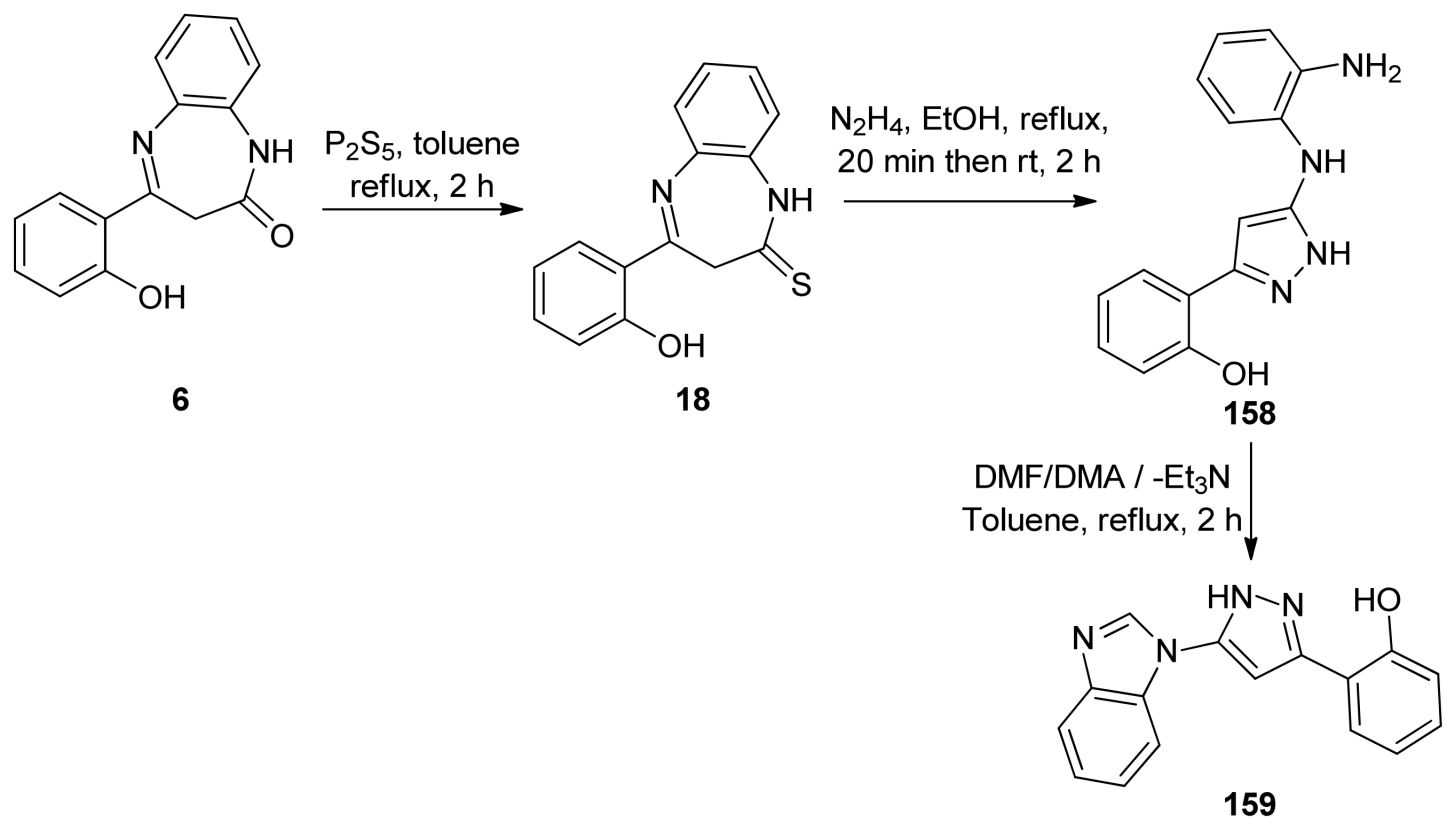
Reactivity of 1,5-benzodiazepin-2-one towards benzopyrano-pyrazole skeleton.

Rafik et al. [65] described the synthesis of the novel compound 4-(2-hydroxyphenyl)-1,3-di*H*-1,5-benzodiazepine-2-thione **18** from precursor 6 (Scheme 34). Subsequent hydrazinolysis of the prepared compound affords **158**, which, upon reaction with an excess of *N*,*N*-dimethylformamide dimethylacetal, furnished 5-(2-hydroxyphenyl-2*H*-pyrazol-3-yl)-1*H*-benzimidazole **159**.

**Scheme 35:**
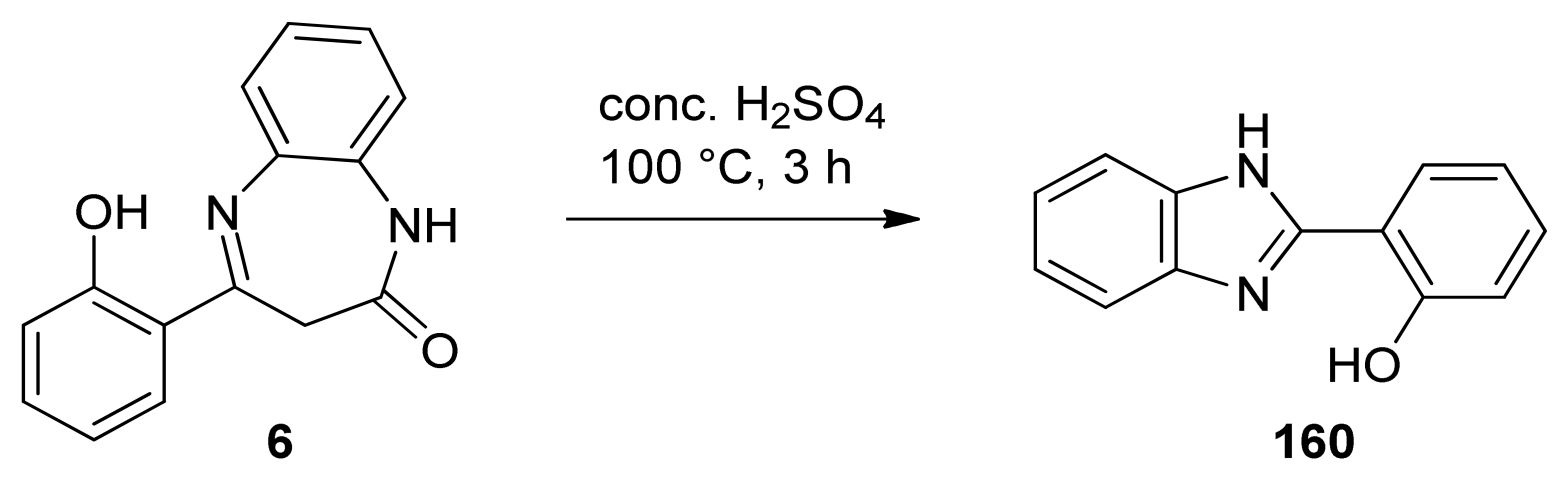
Synthesis of a benzimidazole from a 1,5-benzodiazepin-2-one via ring contraction.

Hamdi and co-workers [66] explored the reduction in the heterocyclic ring size. Refluxing 4-(2-hydroxyphenyl)-2,3-dihydro-1*H*-1,5-benzodiazepin-2-one **6** in concentrated sulfuric acid afforded 2-(2-hydroxyphenyl) benzimidazole **160** as the major product (Scheme 35).

**Scheme 36:**
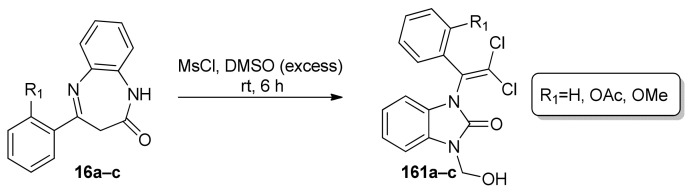
One-pot synthesis of benzimidazolidinones from 1,5-benzodiazepin-2-ones via a 1,3-sigmatropic rearrangement.

Ring contraction reactions have been used as an effective tool for preparing biologically active structures such as benzimidazolones. In this study, Ameni’s group [67] aimed to obtain a novel series of *N*-substituted benzimidazolidinone derivatives **161a–c**. These compounds were synthesized efficiently via a one-pot reaction of 1,5-benzodiazepin-2-one derivatives **16a–c** with DMSO/MsCl, which proceeds through a 1,3-sigmatropic rearrangement of the starting material (Scheme 36).

## 6. Applications

The 1,5-benzodiazepines [68,69,70] constitute a remarkable class of heterocyclic compounds with a broad spectrum of biological activities and therapeutic applications. Traditionally employed as anxiolytic and anticonvulsant agents, their therapeutic potential extends far beyond anxiety and stress-related disorders. Subtle structural modifications within the benzodiazepine scaffold can generate diverse pharmacological profiles. In recent years, increasing research has highlighted novel applications of 1,5-benzodiazepin-2-ones as corrosion inhibitors and has explored their potential in photoluminescence, alongside their established role in medicinal chemistry.

### 6.1. Medicinal Chemistry

**Figure 7:**
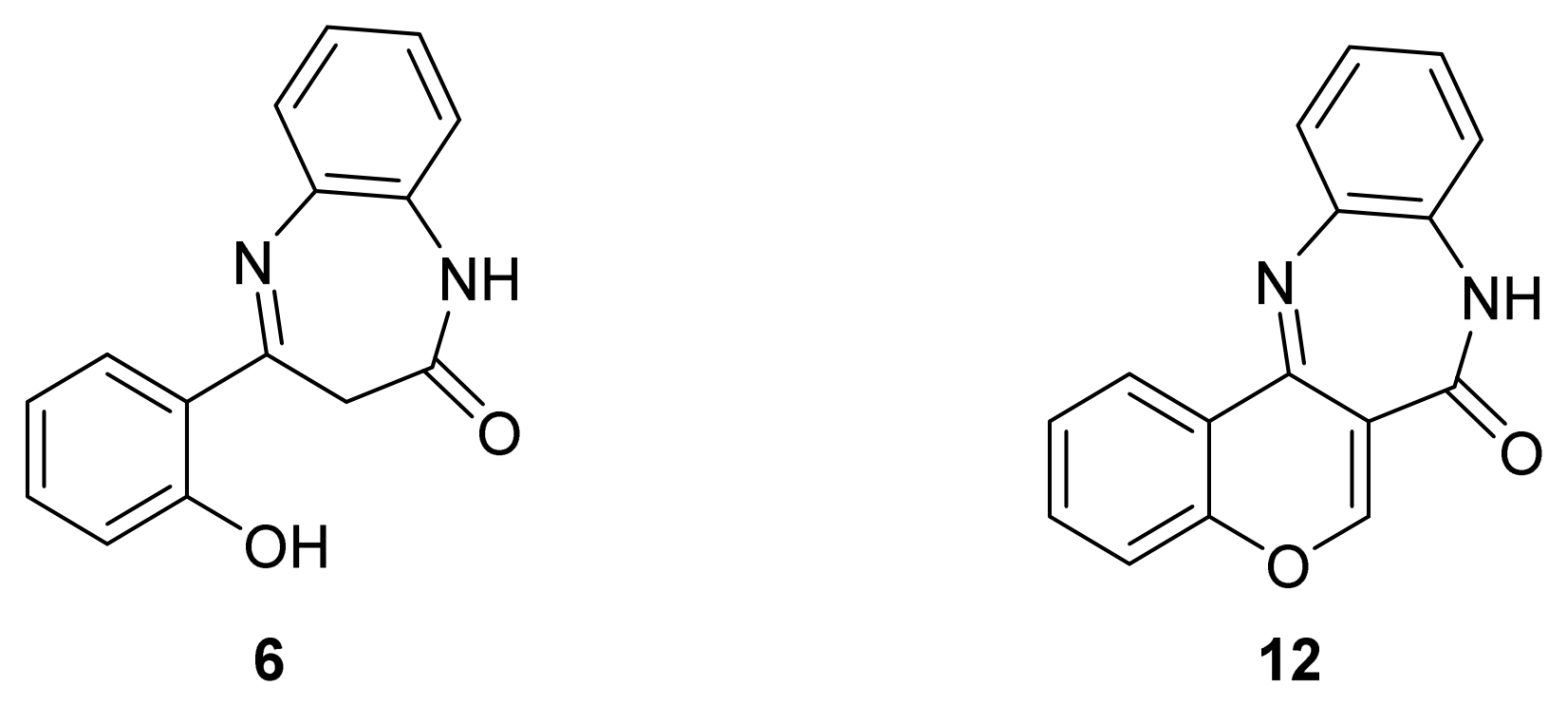
Chemical structures of 4-(2-hydroxyphenyl)-1,5-benzodiazepin-2-one **6** and benzopyrano-1,5-benzodiazepin-2-one **12**.

Wafa and co-workers [71] investigated the potential hypnotic and anticonvulsant activities of two newly synthesized polyheterocyclic compounds derived from the 1,5-benzodiazepin-2-one scaffold (Figure 7). Their findings demonstrated that both compounds exhibited statistically significant pharmacological effects. Notably, molecule **6** showed greater efficacy as a hypnotic agent, whereas compound **12** displayed a more pronounced anticonvulsant activity.

Quadir et al. [72] reported that compound **6** was evaluated for its cytotoxic activity against five human cancer cell lines, including lung (A549), breast (MCF), glioma (C-6), breast (T-47D), and colon (HCT-116), using the MTT assay. The results suggest that this compound exhibits promising cytotoxic properties and may represent a potential drug-like candidate for further development.

**Figure 8:**
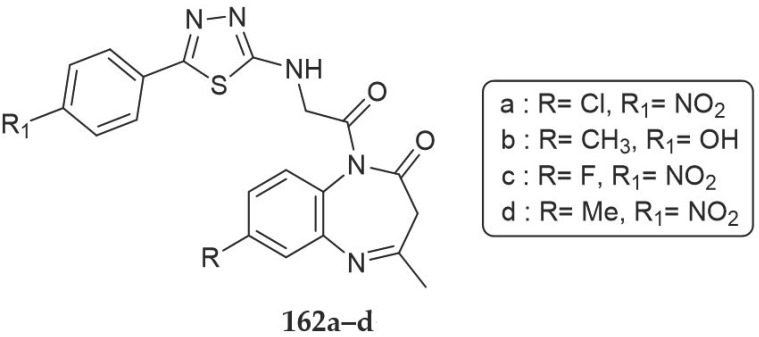
New 1-*H*-Benzo[*b*][1,5]diazepin-2(3*H*)-one derivatives as potential anticonvulsant agents.

Besides, Dinesh et al. [7] investigated the biological effects of 1,5-benzodiazepin-2-one derivatives **162a–d** linked to a 1,3,4-thiadiazole moiety (Figure 8). Structural analysis revealed that substituting a benzoyl group at *N*1 of the benzodiazepine ring and a modified 1,3,4-thiadiazole moiety enhanced the compounds’ anticonvulsant potential.

**Figure 9:**
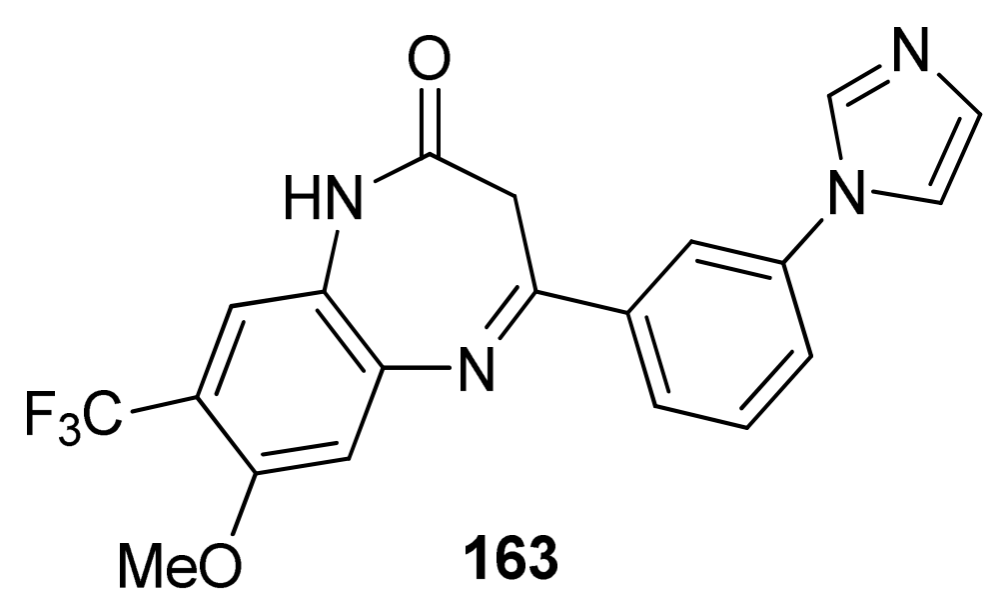
A 1,5-benzodiazepin-2-one derivative as a potential imaging agent for the metabotropic glutamate 2 receptor.

Lynne and co-workers [73] evaluated a series of 1,5-benzodiazepin-2-one derivatives on membranes expressing human mGluR2 receptors using a functional [^35^S] GTPγS assay, revealing high affinity as non-competitive mGluR2 antagonists. Assessment of key physicochemical parameters by HPLC identified compound **163** as a promising SPECT/PET tracer for mGluR2, showing excellent permeability, membrane partitioning, and plasma protein binding (Figure 9).

**Figure 10:**
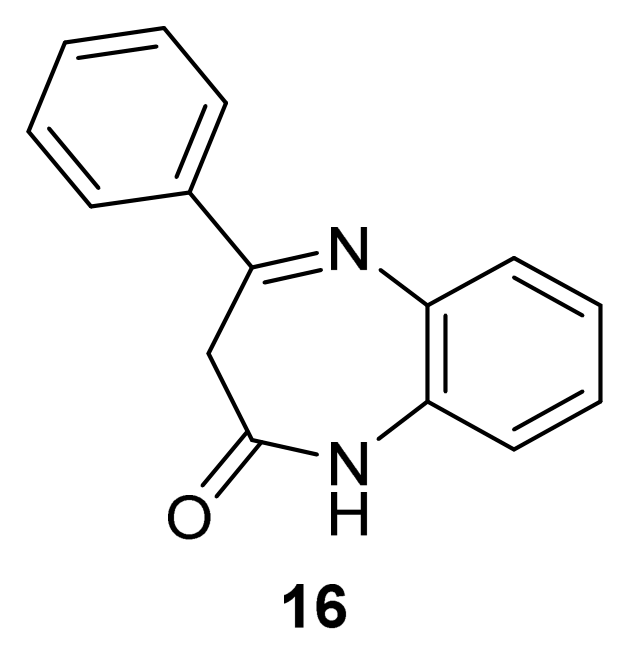
4-Phenyl-1,5-benzodiazepin-2-one as an antiparkinsonian agent.

Ana and co-workers [74] synthesized twenty-three novel 1,5-benzodiazepin-2-ones with promising antioxidant activity and favourable drug-like properties. Among them, compound **16** showed superior effects to curcumin by reducing lipid peroxidation (MDA levels) and restoring GSH levels in SH-SY5Y cells under oxidative stress. These findings highlight 1,5-benzodiazepin-2-ones, particularly compound **16**, as potential leads for the development of new therapeutic agents against Parkinson’s disease and other oxidative stress–related neurodegenerative disorders (Figure 10).

**Figure 11:**
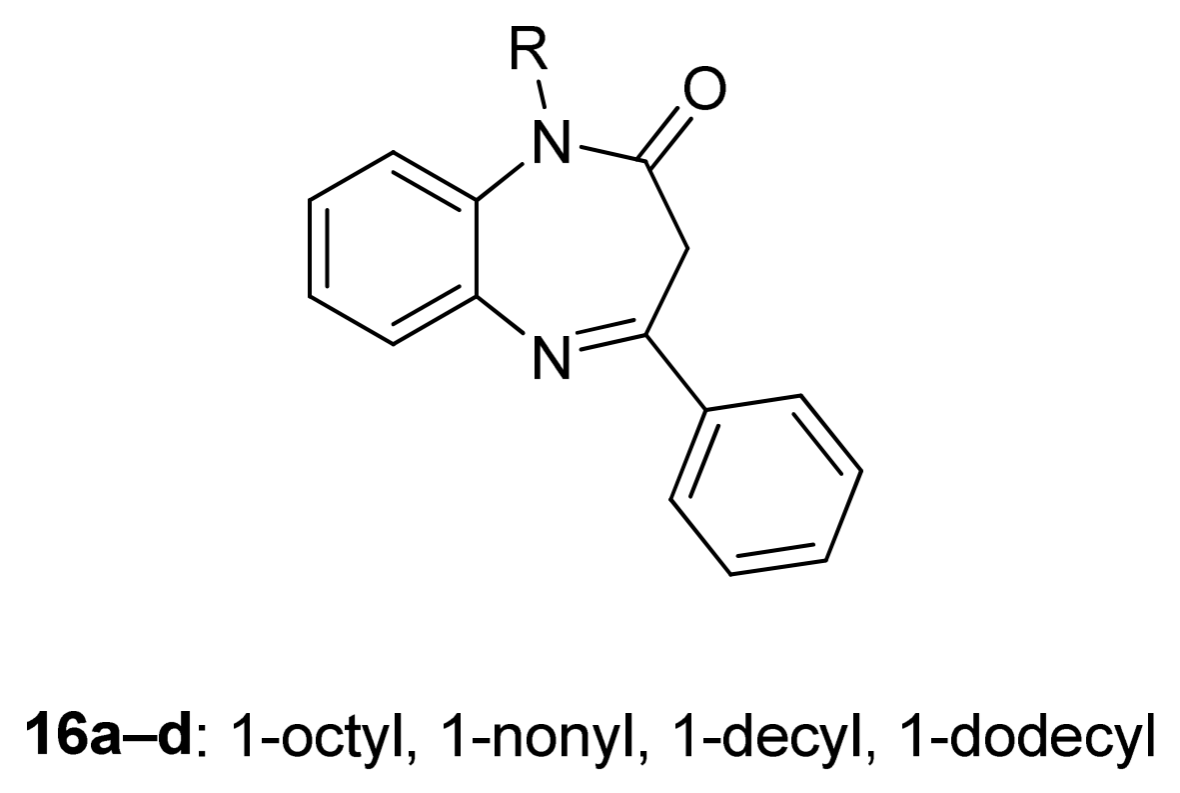
4-Phenyl-1,5-benzodiazepin-2-one derivatives as an analgesic agent.

Terence’s group [75] demonstrated that 4-phenyl-1,5-benzodiazepin-2-one and its *N*-alkyl derivatives (1-octyl, 1-nonyl, 1-decyl, 1-dodecyl) **16a–d** exhibit significant peripheral analgesic activity in mice, comparable to aspirin at the same dose (Figure 11). Additionally, these compounds showed slightly higher central analgesic activity than morphine. Notably, the analgesic efficacy decreased with increasing alkyl chain length, likely because smaller molecules bind more effectively to opioid receptors in the central nervous system.

**Figure 12:**
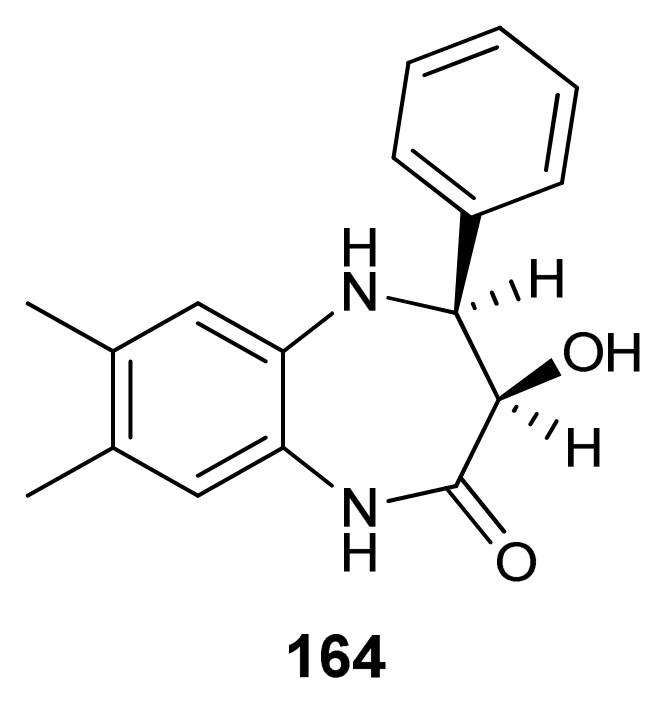
3-Hydroxy-7,8-dimethyl-4-phenyl-1,3,4,5-tetrahydro-2*H*-1,5-benzodiazepin-2-one **164** as an anticancer agent.

Nada and co-workers [76] investigated the anticancer potential of compound **164** through in silico target prediction followed by in vitro validation (Figure 12). Compound **164** was found to inhibit histone deacetylase 1 (HDAC1) and human epidermal growth factor receptor 2 (HER2), leading to cell cycle arrest and apoptosis, thereby demonstrating potent anticancer activity. However, further studies are required to fully elucidate its mechanisms and therapeutic potential.

**Figure 13:**
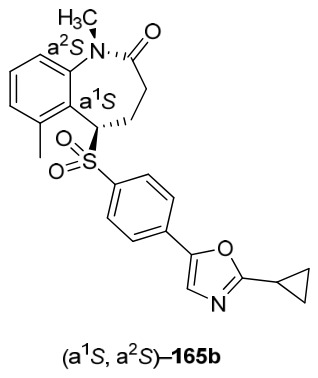
A 5*N*-arylsulfonyl-1,5-benzodiazepin-2-one **165b** as an antiproliferative agent.

Hidetsugu et al. [77] showed that axial chirality induced by the benzenesulfonamide moiety is rare, with only two series of compounds reported to link this feature to biological activity. Notably, 5*N*-arylsulfonyl-1,5-benzodiazepin-2-ones **165b** with antiproliferative activity were synthesized and separated into highly stable (a^1^*R*, a^2^*R*)- and (a^1^*S*, a^2^*S*)-atropisomers (ΔG⧧ ≈ 130 kJ/mol). The (a^1^*S*) configuration and the folded conformation of the molecule were found to be crucial for antiproliferative activity (Figure 13).

**Figure 14:**
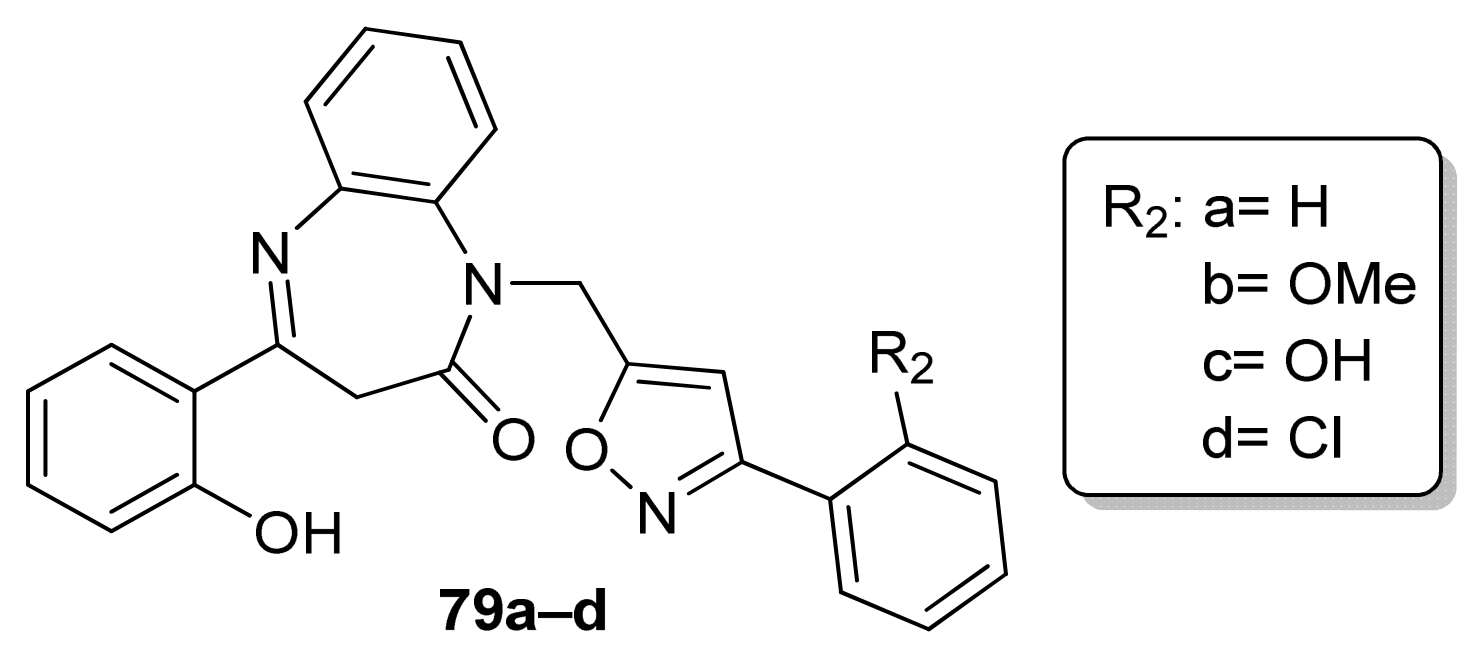
Structures of *N*^1^-isoxazolo-1,5-benzodiazepin-2-one derivatives.

Hanen and co-workers [49] reported that the newly synthesized compounds were evaluated in vitro for their potential antimicrobial activities (Figure 14). Among them, compound **79d** exhibited remarkable antibacterial activity against all tested bacterial strains. In particular, it showed strong inhibitory effects against Pseudomonas aeruginosa and Bacillus subtilis, a result further supported by significant binding interactions revealed through in silico molecular docking studies. Moreover, compound **79b** displayed the highest antifungal activity among the tested compounds.

**Figure 15:**
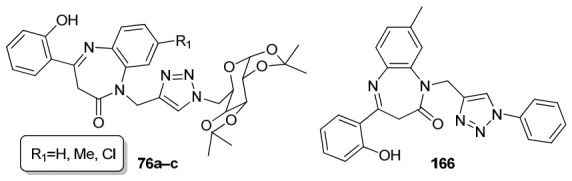
Structures of 1,5-benzodiazepin-2-one derivatives evaluated for antimicrobial activity.

Asma’s group [44] evaluated 1,5-benzodiazepin-2-one derivatives (Figure 15) for their antibacterial and antifungal activities. Compounds **76a–c** exhibited superior antibacterial potential compared to **166**, highlighting the role of methylene linkers, aromatic substituents, and galactopyranosyl–triazole fragments in enhancing activity. In antifungal assays, compound **166** showed the strongest inhibition against Candida albicans and Aspergillus flavus, with ketoconazole as the reference.

**Figure 16:**
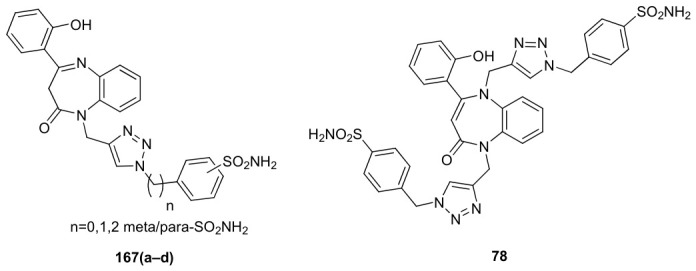
*N*-Triazolo-benzenesulfonamide-1,5-benzodiazepin-2-ones as carbonic anhydrase inhibitors.

Chiraz and co-workers [45] reported two regiospecific synthetic routes to *N*-triazolo-benzenesulfonamide-1,5-benzodiazepin-2-ones (**167a–d** and **78**) (Figure 16). These compounds were evaluated for their inhibitory activity against human carbonic anhydrase isoforms (hCA I, II, IV, VII, IX, XII). Remarkably, the divalent derivative **78** exhibited the strongest inhibition across all tested isoforms, outperforming both the monovalent analogues **167a–d** and the reference drug acetazolamide, and demonstrating potent multivalent effects superior to those of previously reported divalent CA inhibitors.

**Figure 17:**
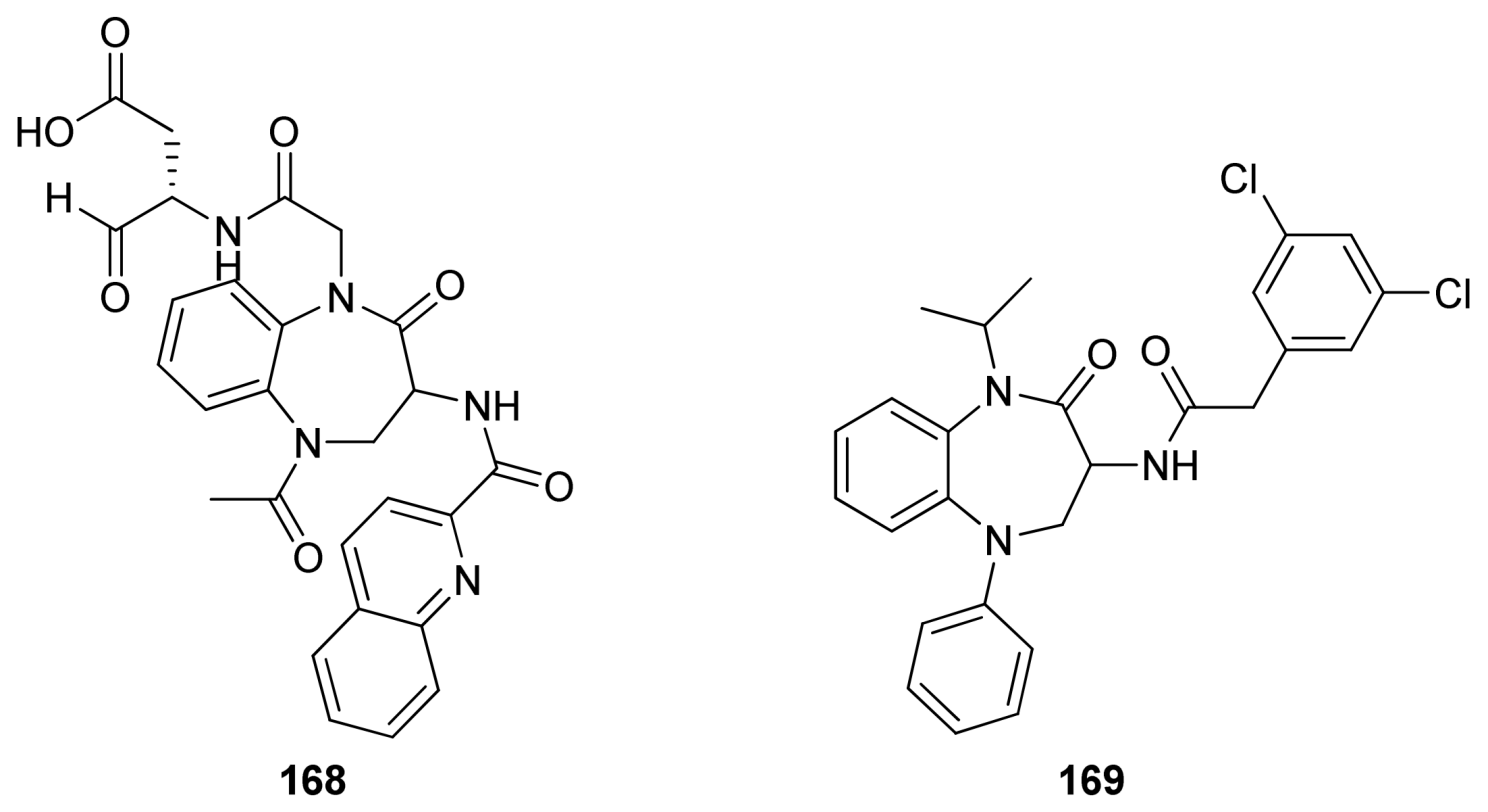
Bioactive 1,5-benzodiazepin-2-ones **168** and **169**.

Horton et al. [78] reported molecules incorporating the 1,5-benzodiazepin-2-one scaffold as privileged substructures (Figure 17), displaying diverse biological activities, including interleukin-1*β* converting enzyme (ICE) inhibition (compound **168**) and delayed rectifier potassium current (IK) blockade (compound **169**).

**Figure 18:**
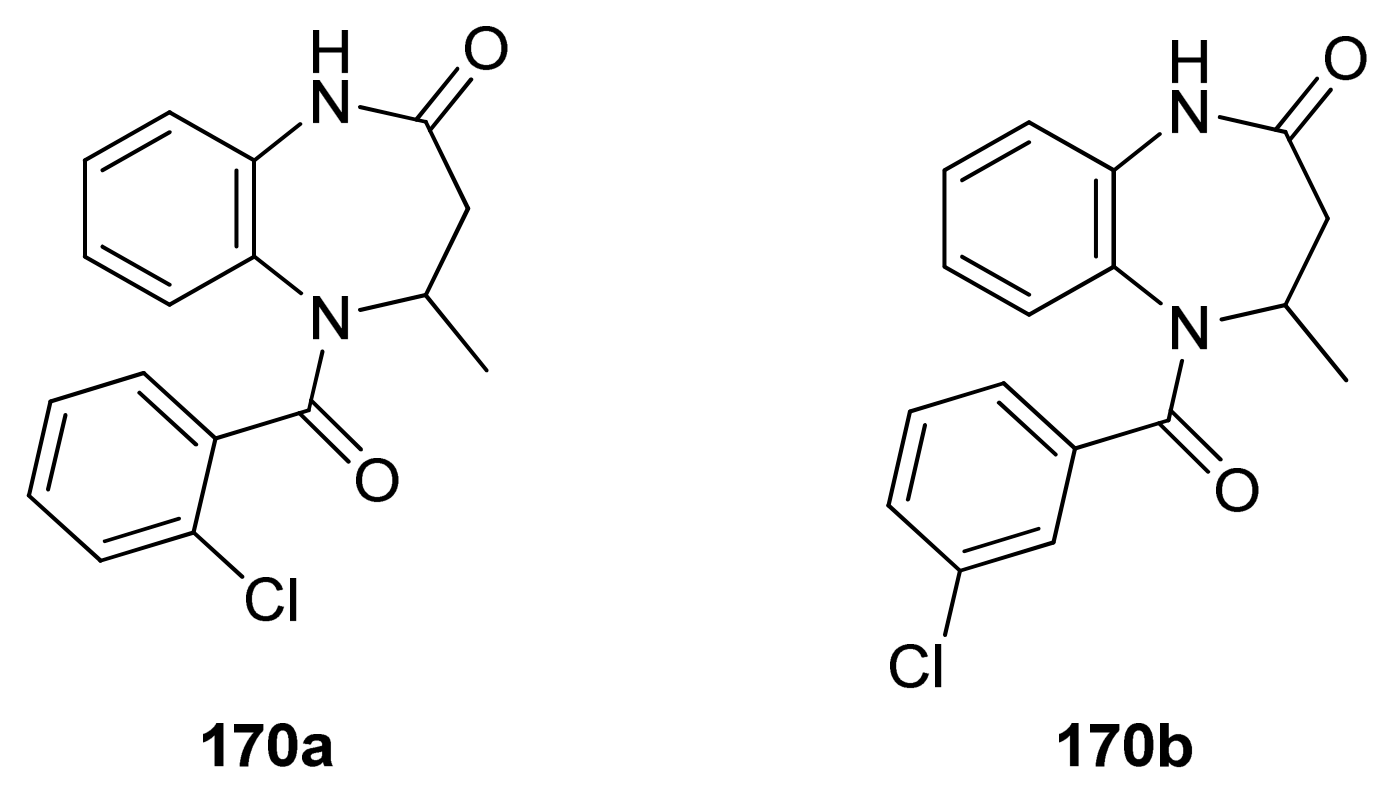
Structures of 1,5-benzodiazepin-2-ones **170a** and **170b** as HIV-1 inhibitors.

Chander et al. [79] described the synthesis of a series of fourteen 5-benzoyl-4-methyl-1,3,4,5-tetrahydro-2*H*-1,5-benzodiazepin-2-one derivatives, which were subsequently evaluated in vitro for their inhibitory activity against the human immunodeficiency virus type 1 (HIV-1) reverse transcriptase (RT) enzyme using an ELISA-based colorimetric assay. Among the tested compounds, **170a** and **170b** demonstrated the highest antiviral potency (Figure 18), exhibiting IC_50_ values of 8.62 µM and 6.87 µM, respectively.

**Figure 19:**
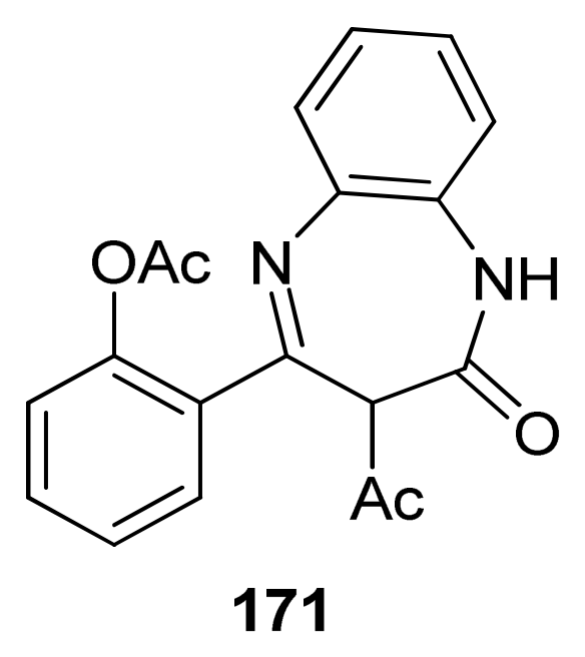
Acetylated 1,5-benzodiazepin-2-one **171** as an anti-inflammatory agent.

Ameni and co-workers [14] demonstrated through in silico molecular docking studies that the acetylated 1,5-benzodiazepin-2-one derivative **171** (Figure 19) exhibited promising binding affinity toward the COX-2 receptor as an anti-inflammatory agent, surpassing aspirin due to favorable hydrogen-bonding and hydrophobic interactions.

**Figure 20:**
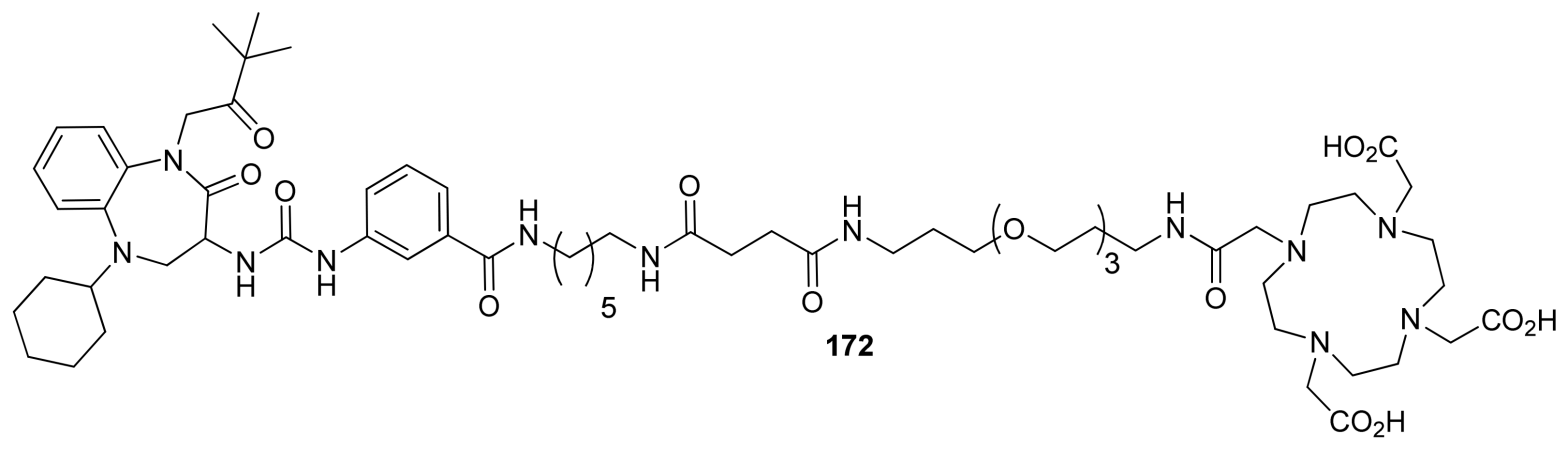
Synthesis of **172** for targeting the CCK-2 Receptor.

Verona et al. [80] demonstrated that a radiotracer based on the ligand **172** (Figure 20), targeting the CCK-2R receptor overexpressed in certain tumors, was synthesized and labeled with indium-111. It is stable in human blood and efficiently accumulates in A549 tumor cells in vitro. In mice bearing A549 xenografts, SPECT/CT imaging clearly visualized the tumors as early as 4 h post-injection, with tracer retention up to 24 h, demonstrating its potential for targeted tumor bioimaging.

**Figure 21:**
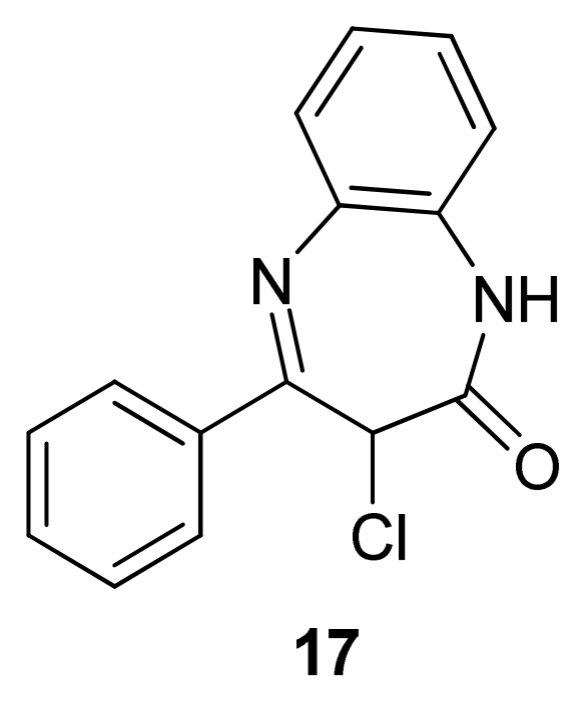
3-Chloro-1,5-Benzodiazepin-2-one **17** as promising leads for potent anxiolytics.

Ameni and co-workers [13] showed that the newly synthesized 3-chloro-1,5-benzodiazepin-2-one **17** (Figure 21) binds the GABA(A) receptor through hydrogen bonds and hydrophobic interactions, supporting their potential as lead compounds for anxiolytic agents. Additional physicochemical and bioactivity studies revealed diverse physiological effects via GPCR and nuclear receptor interactions, highlighting these derivatives as promising candidates for the development of anxiolytic and other therapeutic agents.

To facilitate comparison among the reported biological activities of 1,5-benzodiazepin-2-one derivatives, the most representative compounds along with their quantitative activity data are summarized in Table 1.

### 6.2. Photoluminescent Materials

**Figure 22:**
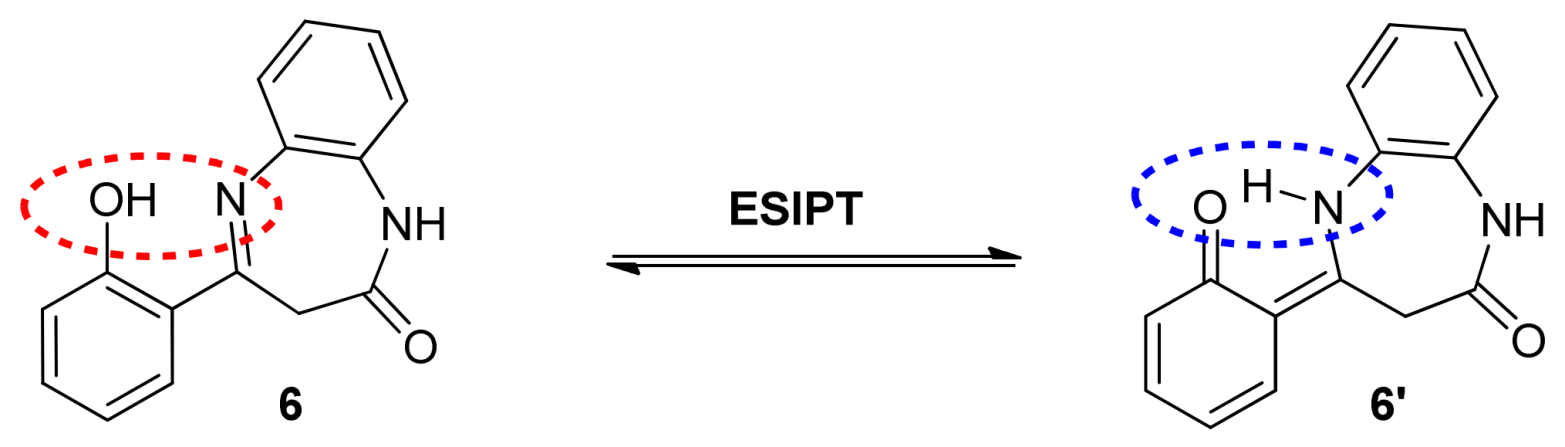
The ESIPT process of target **6**.

Due to their broad applications in bioimaging, sensing, and optoelectronics, photoluminescent materials have attracted increasing attention in recent years. However, only a limited number of molecular families are known to exhibit fluorescence in the aggregated or solid state via the excited-state intramolecular proton transfer (ESIPT) process. Xia et al. [81] used electronic structure calculations and nonadiabatic surface-hopping simulations to study 1,5-benzodiazepin-2-one. They identified three S_1_ → S_0_ decay pathways: an ESIPT-driven keto route and two enol channels via S_1_/S_0_ conical intersections, which account for the low fluorescence quantum yield and provide mechanistic insight for similar systems (Figure 22).

**Scheme 37:**
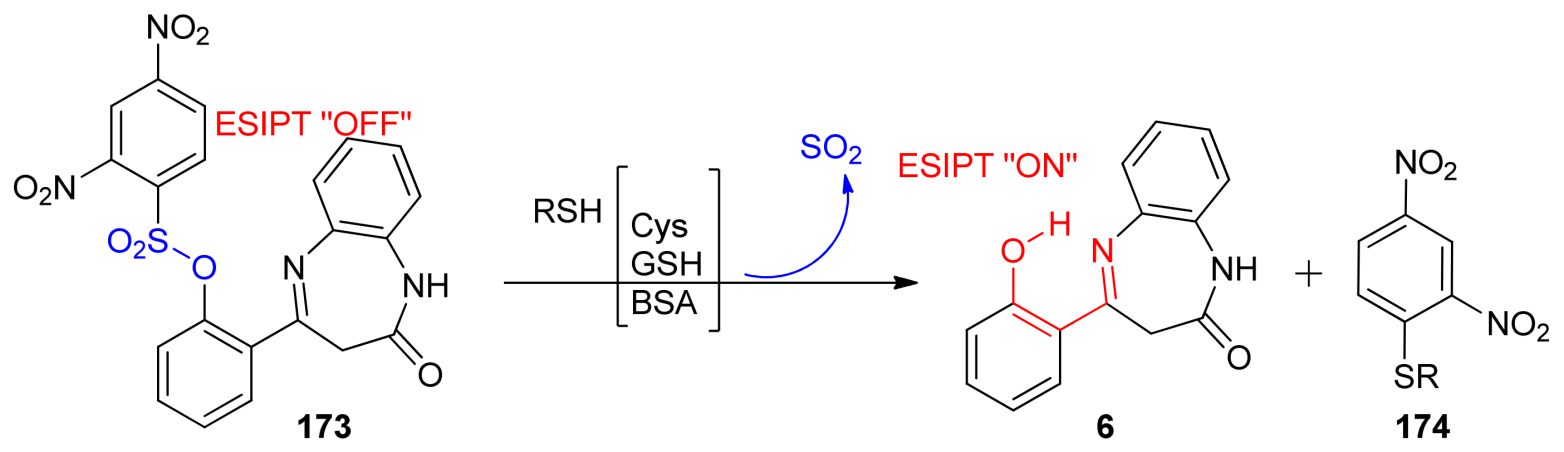
Mechanism of biothiol detection by the probe **173**.

Hasan and co-workers [11] reported the preparation and photochemical characterization of an underexplored family of 1,5-benzodiazepin-2-one derivatives, showing through photophysical studies and X-ray diffraction analysis that their photoluminescence (520–655 nm) is predominantly governed by ESIPT. The 1,5-benzodiazepin-2-one scaffold and its derivatives have been employed as fluorogenic probes for the selective detection of biothiols (Scheme 37).

**Scheme 38:**
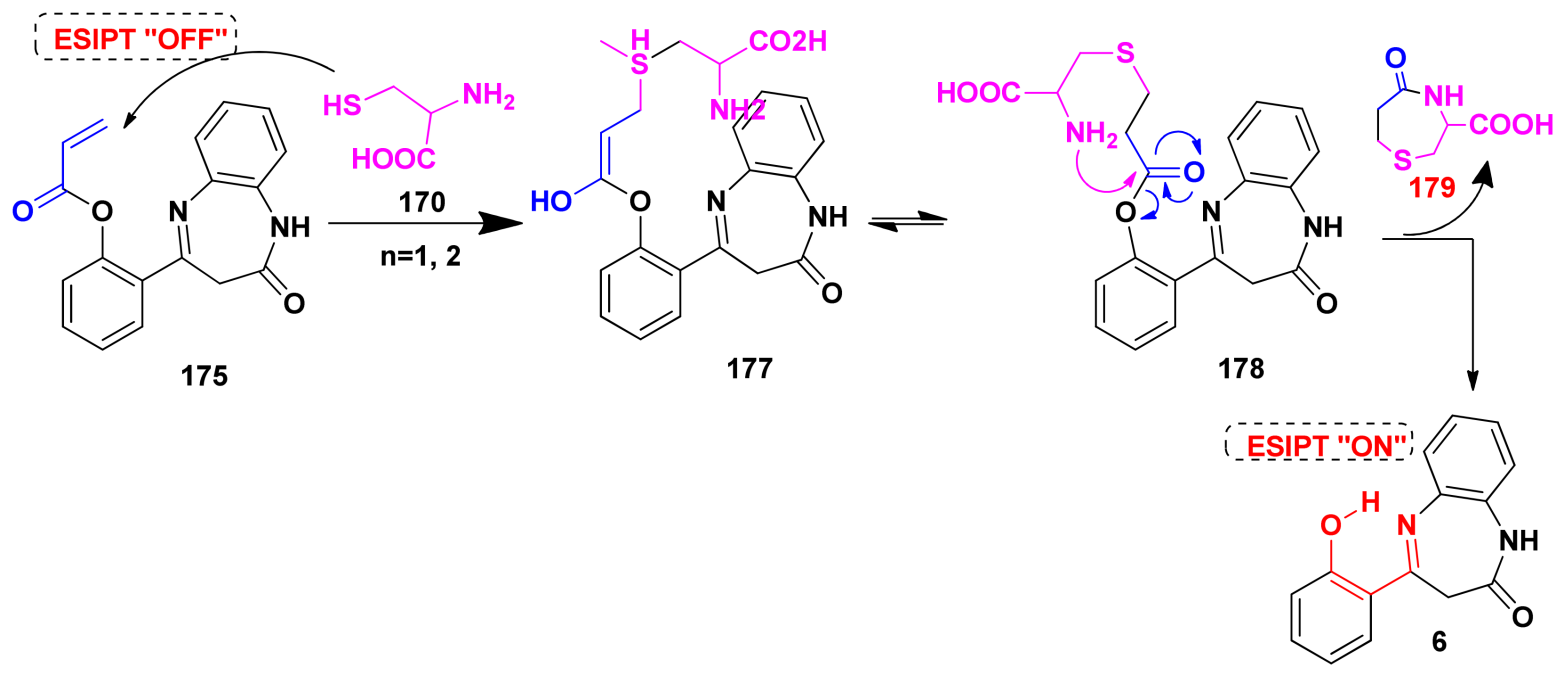
Mechanism of cysteine detection by probe **175**.

Following these encouraging results, the fluorophore was masked with an acrylate group to achieve selective detection of cysteine over other biological species. Hasan et al. [11] demonstrated that while a thiol–ether bond is formed through the conjugate addition of thiols to acrylates, the fluorescent molecule is released only during the subsequent intramolecular ester aminolysis step (Scheme 38).

**Scheme 39:**
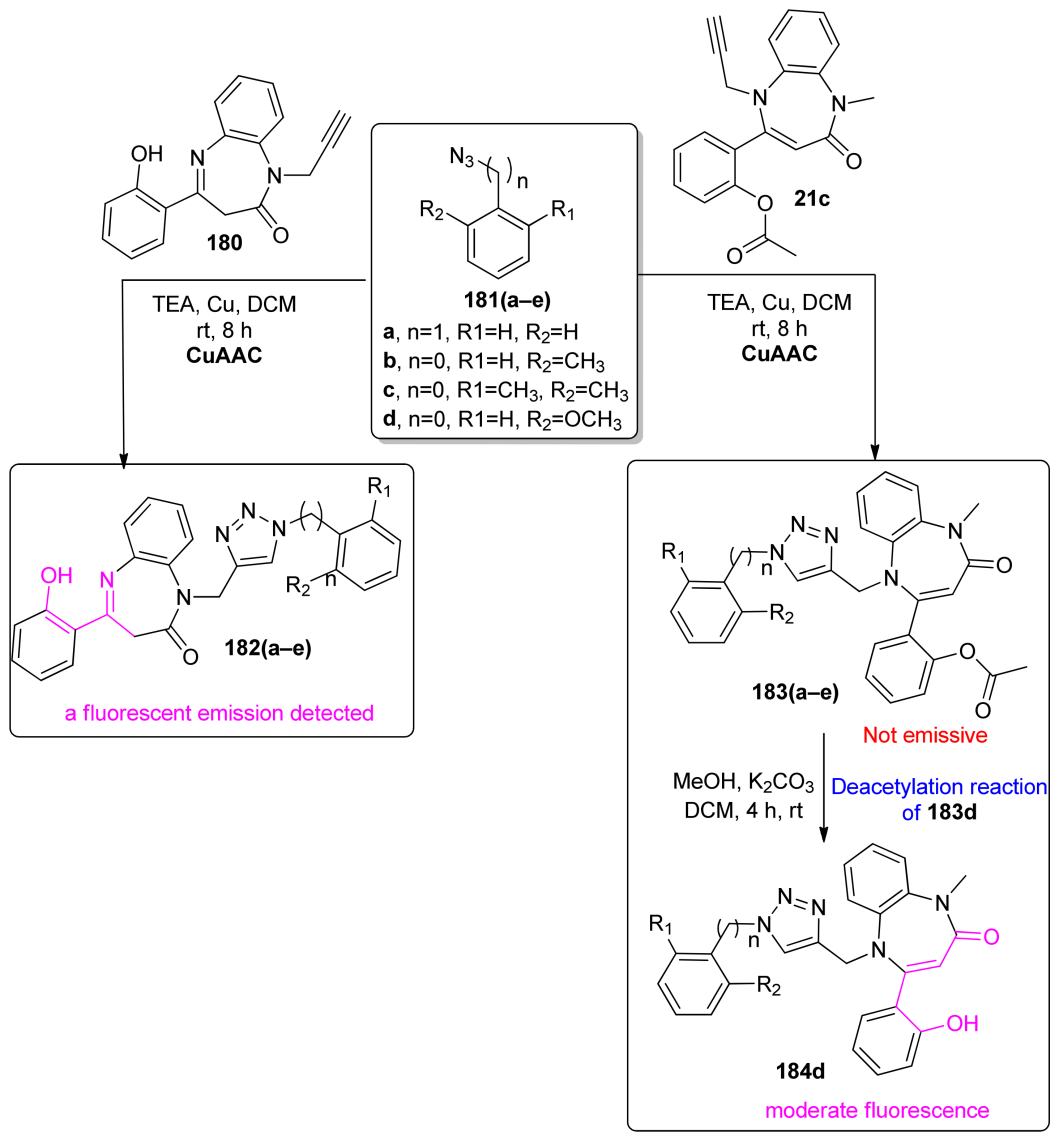
Photoluminescent studies of new 1,5-benzodiazepin-2-one derivatives.

Recently, Chiraz et al. [45] investigated the photophysical properties of triazolo-benzodiazepine hybrids using absorption and fluorescence spectroscopy (Scheme 39). The hydroxy-substituted derivatives **182a–e** exhibited fluorescence in the aggregated state, whereas the *o*-acetylated derivatives **183a–e** were non-emissive. Deprotection of the –OAc group in **183d** restored moderate fluorescence in product **184d**, confirming that ESIPT occurs only when the hydroxyl proton is free. Computational studies on **182e** and **184d** further clarified their fluorescence behaviour in aqueous mixtures.

**Scheme 40:**
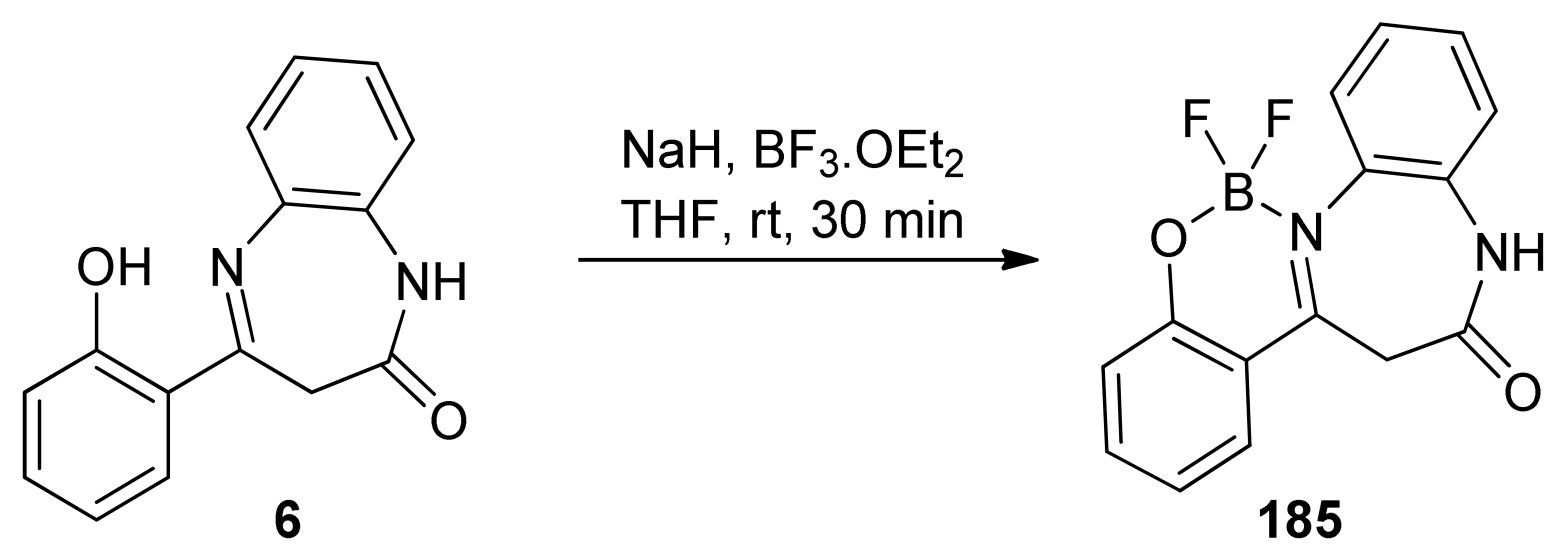
Example of fluorescent cyclic boranil based on 1,5-benzodiazepin-2-one scaffold.

In addition, it has been reported that 1,5-benzodiazepin-2-one exhibit fluorescence in the aggregated and solid states via an excited-state intramolecular proton transfer (ESIPT) process, whereas they are non-emissive in organic solvents. In these solvents, intramolecular motions disrupt the critical hydrogen bond between the phenolic OH and the imine nitrogen, thereby quenching the ESIPT mechanism. To address this limitation, Hasan and co-workers [12] developed a new family of fluorescent 1,5-benzodiazepin-2-one borate complexes (Scheme 40). Unlike their uncomplexed analogues, most of these complexes displayed strong emission in both solution and the solid state (426–596 nm). Substituent studies showed that halogenation at position 8 enhanced fluorescence, whereas electron-donating groups quenched it. A proof-of-concept study further demonstrated that the amide moiety could be functionalized, offering a handle for targeted modifications.

### 6.3. Corrosion Inhibitors

Corrosion is a major issue affecting numerous industrial sectors. Annually, an estimated one-quarter of global steel production—approximately 150 million tons—is lost to corrosion [82,83,84]. This phenomenon can manifest in various forms, from simple uniform corrosion to more complex patterns observed in harsh industrial environments. Depending on the sector, different mitigation strategies are employed, often chosen as a compromise between technical requirements and economic constraints. Among these, the use of inhibitors is considered one of the most economical and effective approaches to limit corrosive attack on metals [85,86,87].

**Figure 23:**
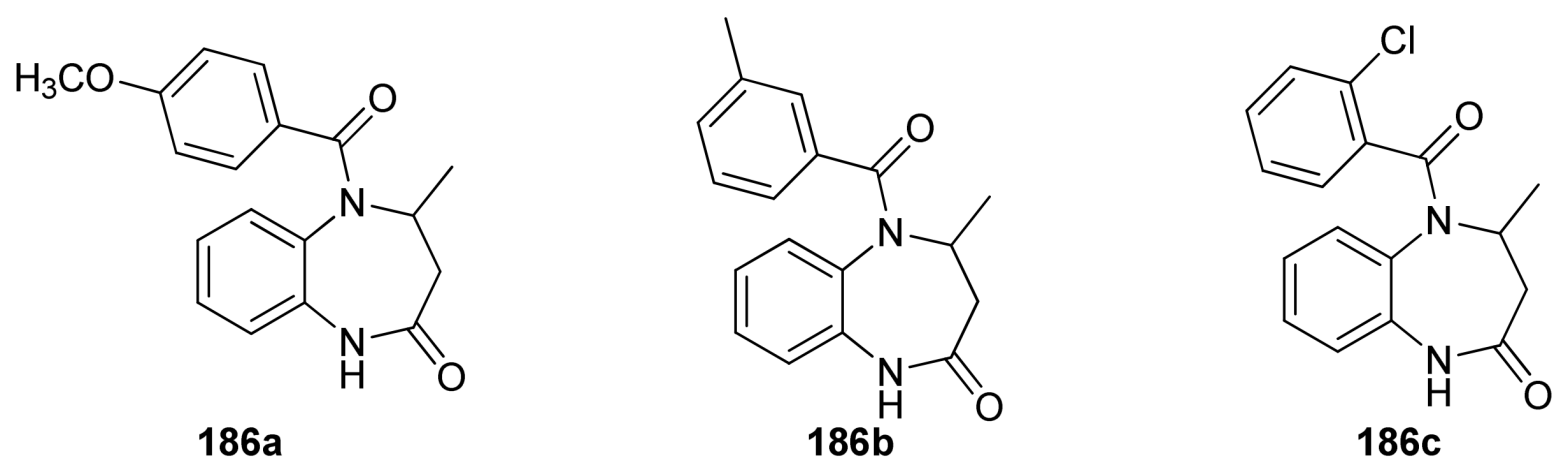
Molecular structures of 1,5-benzodiazepin-2-one derivatives evaluated as corrosion inhibitors for mild steel in acidic medium.

The most effective corrosion inhibitors are typically organic compounds containing heteroatoms (*O*, *N*, *S*, *P*) or multiple bonds, which adsorb onto the metal surface to form protective layers and reduce the corrosion rate [88,89,90]. Benzodiazepine derivatives, owing to their structural features, have been studied as potential inhibitors in acidic media [91,92,93]. Abdo and co-workers [94] evaluated three newly synthesized 1,5-benzodiazepin-2-ones bearing 4-methoxybenzoyl (**186a**), 3-methylbenzoyl (**186b**), and 2-chlorobenzoyl (**186c**) groups as corrosion inhibitors for mild steel in 1 M HCl (Figure 23). Density functional theory calculations identified the active sites responsible for adsorption. Both experimental and theoretical results confirmed the inhibition efficiency order: 4-methoxybenzoyl > 3-methylbenzoyl > 2-chlorobenzoyl.

**Figure 24:**
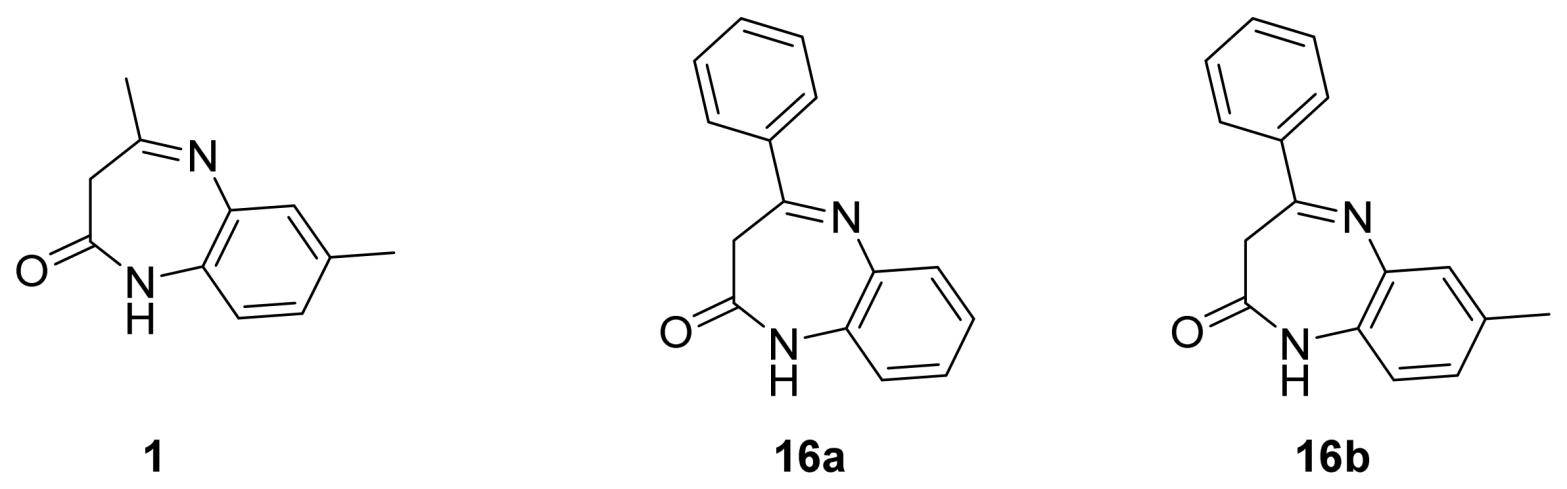
Chemical structures of the investigated 1,5-benzodiazepin-2-one derivatives.

In addition, Ibrahim et al. [95] studied three 1,5-benzodiazepin-2-one derivatives 4,7-dimethyl-1,5-benzodiazepin-2-one (**1**), 3-phenyl-1,5-benzodiazepin-2-one (**16a**), and 4-methyl-7-phenyl-1,5-benzodiazepin-2-one (**16b**)—as iron corrosion inhibitors in acidic medium using DFT and Monte Carlo simulations (Figure 24). The derivatives exhibited a strong tendency to displace pre-adsorbed aggressive species from the iron surface and to form protective films. All molecules adsorbed in a parallel orientation, providing better surface coverage. Among them, compound **16b** was predicted to act as a moderate inhibitor for Al and Cu but was less effective for Sn in acidic environments.

**Figure 25:**
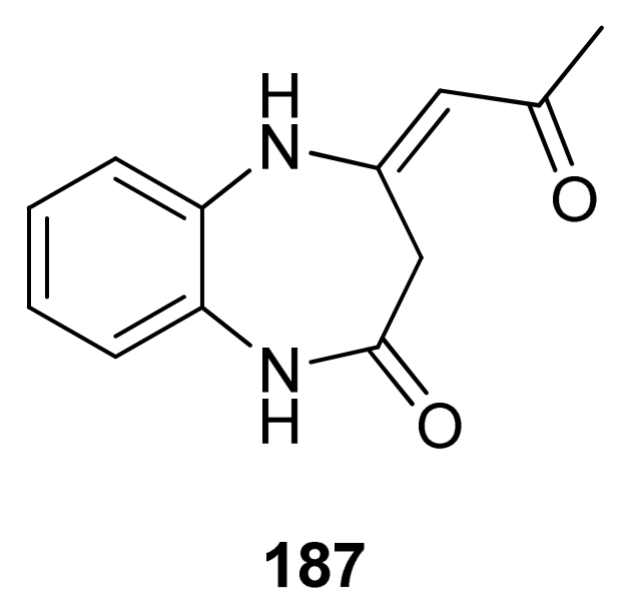
Chemical structure of 4-(2-oxo-propylidene)-1,5-benzodiazepin-2-one **187**.

In this context, Laabaissi et al. [96] investigated the corrosion inhibition performance of 4-(2-oxo-propylidene)-1,5-benzodiazepin-2-one **187** on carbon steel in 2 M phosphoric acid using potentiodynamic polarization and electrochemical impedance spectroscopy (Figure 25). The results indicated that the inhibition efficiency of 4-(2-oxo-propylidene)-1,5-benzodiazepin-2-one (OPBz) increased with higher concentrations but decreased at elevated temperatures. The same molecule was also studied by Ksama and co-workers [97], who demonstrated that (*Z*)-7-methyl-4-(2-oxopropylidene)-4,5dihydro-1*H*-1,5-benzodiazepin-2-one (BZ-Me) acts as an effective corrosion inhibitor for E24 steel in aggressive solutions using potentiodynamic polarization (PDP), electrochemical impedance spectroscopy (EIS), and scanning electron microscopy with energy-dispersive spectroscopy (SEM-EDS). EIS results indicated a significant reduction in the corrosion rate, while SEM-EDS confirmed the formation of a protective layer on the steel surface. These findings highlight the strong inhibitory effect of 1,5-benzodiazepin-2-one **187** in 1 M HCl and its potential for diverse industrial applications.

**Figure 26:**
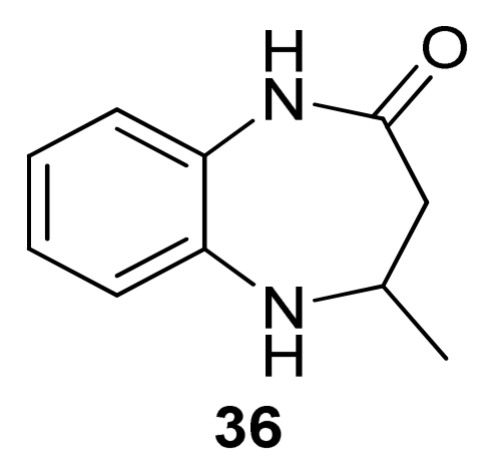
Structure of 4-methyl-2,3-dihydro-1*H*-1,5-benzodiazepin-2-one **36**.

Similarly, El Ghayati et al. [98] studied the effect of 4-methyl-2,3-dihydro-1*H*-1,5-benzodiazepin-2-one **36** on steel corrosion in an acidic medium (Figure 26). Increasing the concentration of compound **36** significantly reduced the corrosion current density, while the inhibition efficiency remained largely unchanged with rising temperature. The study combined potentiodynamic polarization, weight-loss measurements, and electrochemical impedance spectroscopy (EIS) with density functional theory calculations. Quantum chemical parameters were evaluated to correlate the experimental results with theoretical insights, providing a deeper understanding of the inhibition mechanism.

## 7. Conclusions and Perspectives

This comprehensive review has highlighted the remarkable versatility of the 1,5-benzodiazepin-2-one scaffold in both synthetic and application-oriented contexts. While numerous efficient synthetic strategies ranging from classical condensations to modern catalytic and microwave-assisted approaches enable access to diverse heterocyclic derivatives, practical limitations such as the need for protecting groups and the low solubility of certain *ESIPT* fluorophores should be acknowledged. The scaffold’s broad functionalization potential, especially at the *C*-3 and *N*-1 positions, continues to support the development of hybrid, fused, spiro, and contracted ring systems, significantly expanding chemical space. Beyond its established pharmacological activities, including anxiolytic, anticancer, antimicrobial, and analgesic effects, the framework shows promise in materials science, particularly for photoluminescent applications via *ESIPT* and as effective corrosion inhibitors. Future research directions could focus on overcoming current synthetic limitations, exploring electrochemical and flow-chemistry methodologies, and validating bioactive derivatives in vivo. Collectively, these strategies will guide the rational design of advanced 1,5-benzodiazepin-2-one-based structures with optimized properties for both biomedical and materials applications, unlocking their full potential.

## Figures and Tables

**Table 1:** Summary of the quantitative biological activities of the most benzodiazepin-2-one derivatives mentioned above.

Compounds	Biological Activities	Target/Model	Activity Value	Ref.
**12**	Anticonvulsant	Mice (PTZ-induced seizure model)	ED_50_ = 50 mg/kg	[71]
**6**	Hypnotic	Mice (hexobarbital-induced hypnosis model)	ED_50_ = 0.17 mg/kg	[71]
	cytotoxic	cell lines: A-549 MCF-7, C-6, T-47D, HCT-116	IC50 = 48.5 ± 0.38 μmol/L 34.2 ± 0.42 μmol/L 43.9 ± 0.13 μmol/L 40.6 ± 0.19 μmol/L 38.5 ± 0.12 μmol/L	[72]
**162a** and **162c**	Anticonvulsant	ScPTZ-induced seizures test	80% protection at 0.4 mg/kg	[7]
**163**	imaging agents of the metabotropic glutamate 2 receptor	human recombinant mGluR2 receptor	IC50 = 91 ± 30 nM	[73]
**16** Ana’s group	Neuroprotective	differentiated SH-SY5Y cells (diffSH-SY5Y)	LD50 = 494 M	[74]
**16b** Terence’s group	analgesic	Swiss mice	at 50 mg/kg: % inhibition: 51.39 ± 6.55%	[75]
**164**	anticancer	HER2 and HDAC1 proteins	IC_50_ = 0.023 nM and 0.041 nM, respectively	[76]
**165b**	Antiproliferative	HCT-116 and A549	GI50 = 0.37 μM (HCT-116) and 0.62 (A-549)	[77]
**79d**	antibacterial	*Staphylococcus aureus*/*Bacillus subtilis*/*Enterococcus faecalis*	MIC = 7.81 μg/mL (*B. subtilus* ATCC 6633)	[49]
**79b**	antifungal	*Candida albicans*/*Candida parapsilosi*/*Candida glabrata*	MIC = 12.8 μg/mL (*C. glabrata*/*C. albicans*)	
**76a–c**	antibacterial	against 6 Gram-positive bacteri/3 Gram-negative bacteri	Exp: (*S. epidermidis*(+), MIC = 32 g/mL	[44]
**166**	Antifungal	*Candida albicans* and the *Aspergillus flavus*	MIC in g/mL = 64 (*Aspergillus flavus*)/32 (*Candida albicans*)	
**78**	carbonic anhydrase inhibitors	against human carbonic anhydrase isoforms (hCA I, II, IV, VII, IX, XII)	especially against hCA II, hCA I, and hCA XII: Ki from 98 nM to 16 nM	[45]
**170a** and **170b**	Anti HIV-1	ELISA-based colorimetric assay	IC_50_ = 8.62 µM and 6.87 µM, respectively.	[79]
**171**	Anti- inflammatory	-	-	[14]
**172**	tumor-targeting for SPECT/CT imaging	A549 human lung cancer cells	Tumor uptake: 2.36 ± 0.26% IA/g (4 h), 1.52 ± 0.71% IA/g (24 h); blocked: 0.84 ± 0.33% IA/g (24 h); T/M = 15.72, T/B = 10.19	[80]
**17**	Anxiolytic agent	-	-	[13]

## Data Availability

No new data were created or analyzed in this study. Data sharing is not applicable to this article.
